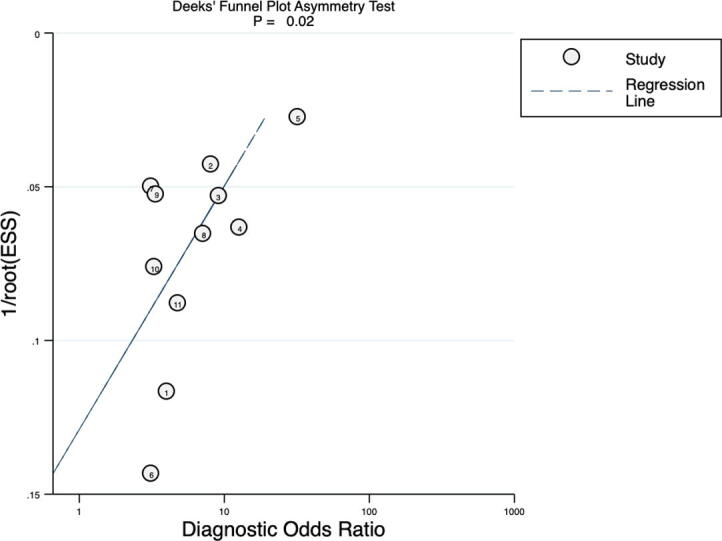# Molecular Biomarkers for the Detection of Clinically Significant Prostate Cancer: A Systematic Review and Meta-analysis

**DOI:** 10.1016/j.euros.2022.10.017

**Published:** 2022-11-10

**Authors:** Tasmania del Pino-Sedeño, Diego Infante-Ventura, Aythami de Armas Castellano, Pedro de Pablos-Rodríguez, Antonio Rueda-Domínguez, Pedro Serrano-Aguilar, María M. Trujillo-Martín

**Affiliations:** a Canary Islands Health Research Institute Foundation (FIISC), Tenerife, Spain; b The Spanish Network of Agencies for Health Technology Assessment and Services of the National Health System (RedETS), Madrid, Spain; c European University of the Canary Islands (UEC), Santa Cruz de Tenerife, Spain; d Network for Research on Chronicity, Primary Care, and Health Promotion (RICAPPS), Tenerife, Spain; e Department of Urology, Valencian Institute of Oncology Foundation, Valencia, Spain; f Doctoral School of University of Las Palmas de Gran Canaria, Las Palmas, Spain; g Research Institute of Biochemical and Health Sciences, Barcelona, Spain; h Medical Oncology Intercenter Unit, Regional and Virgen de la Victoria University Hospitals, IBIMA, Málaga, Spain; i Research Network on Health Services in Chronic Diseases (REDISSEC), Madrid, Spain; j Institute of Biomedical Technologies (ITB). University of La Laguna, Tenerife, Spain; k Evaluation Unit (SESCS), Canary Islands Health Service (SCS), Tenerife, Spain

**Keywords:** Prostatic neoplasms, Molecular markers, Clinically significant cancer, Systematic review, Meta-analysis

## Abstract

**Context:**

Prostate cancer (PCa) is the second most common type of cancer in men. Individualized risk stratification is crucial to adjust decision-making. A variety of molecular biomarkers have been developed in order to identify patients at risk of clinically significant PCa (csPCa) defined by the most common PCa risk stratification systems.

**Objective:**

The present study aims to examine the effectiveness (diagnostic accuracy) of blood or urine-based PCa biomarkers to identify patients at high risk of csPCa.

**Evidence acquisition:**

A systematic review of the literature was conducted. Medline and EMBASE were searched from inception to March 2021. Randomized or nonrandomized clinical trials, and cohort and case-control studies were eligible for inclusion. Risk of bias was assessed using the Quality Assessment of Diagnostic Accuracy Studies-2 (QUADAS-2) tool. Pooled estimates of sensitivity, specificity, and area under the curve were obtained.

**Evidence synthesis:**

Sixty-five studies (*N* = 34 287) were included. Not all studies included prostate-specific antigen-selected patients. The pooled data showed that the Prostate Health Index (PHI), with any cutoff point between 15 and 30, had sensitivity of 0.95–1.00 and specificity of 0.14–0.33 for csPCa detection. The pooled estimates for SelectMDx test sensitivity and specificity were 0.84 and 0.49, respectively.

**Conclusions:**

The PHI test has a high diagnostic accuracy rate for csPCa detection, and its incorporation in the diagnostic process could reduce unnecessary biopsies. However, there is a lack of evidence on patient-important outcomes and thus more research is needed.

**Patient summary:**

It has been possible to verify that the application of biomarkers could help detect prostate cancer (PCa) patients with a higher risk of poorer evolution. The Prostate Health Index shows an ability to identify 95–100 for every 100 patients suffering from clinically significant PCa who take the test, preventing unnecessary biopsies in 14–33% of men without PCa or insignificant PCa.

## Take Home Message

The Prostate Health Index test had high diagnostic accuracy for the detection of clinically significant prostate cancer. Its incorporation in the diagnostic process could reduce the necessary biopsies. There is a lack of evidence on the effect of this test on clinical outcomes.

## 1 Introduction

Prostate cancer (PCa) is a major health problem, with approximately 1.4 million cases diagnosed worldwide each year [1]. It is the second most common cancer in males after lung cancer worldwide [2], and its prevalence increases with each additional year of age [3]. The mean age of PCa onset is 65 yr and the majority of PCa patients are diagnosed from then onwards, with the age group of 70–75 yr having the highest incidence rate [4].

PCa often progresses slowly and has a prolonged preclinical phase. Therefore, many men with PCa die from causes other than PCa and without evidence of pathological manifestation [3].

Traditionally, diagnosis and staging of PCa have been based on prostate-specific antigen (PSA) level, digital rectal examination (DRE), and transrectal ultrasound guided prostate biopsy.

Serum PSA measurement is the reference standard for the early detection of PCa. However, PSA level does not exclusively increase in malignant pathology, as high levels can also be observed in benign prostatic pathologies such as benign prostatic hyperplasia, prostatitis, other urinary tract infections, and even acute urine retention. Moreover, PSA cannot discriminate between indolent PCa (iPCa) and aggressive tumors.

Most men with positive screening results (elevated PSA levels or abnormal DRE) who undergo prostate biopsy will not have PCa. Approximately two-thirds of men with an elevated PSA level can expect a false positive test result [5]. Moreover, biopsy procedures are related to complications such as pain, bleeding, and sepsis, and the related consequences on the utilization of health resources. However, the most serious harm of PCa screening may be overdiagnosis, which may result in subsequent overtreatment [6]. Consequently, strategies to differentiate iPCa from aggressive tumors are necessary [1]. Current European Urological Association guidelines recommend the use of risk stratification tools, such as risk calculators and magnetic resonance imaging (MRI), and biomarker tests for the prediction of a positive prostate biopsy as reflex tests after an elevated PSA level [7].

Recently, there has been an expansion in the availability of new molecular blood and urine test biomarkers that can be used to support prostate biopsy decisions, providing more individualized risks for PCa, distinguishing between clinically significant PCa (csPCa) and iPCa, or predicting the prognosis of patients already diagnosed [8]. However, no consensus has been reached on the use of these tests in routine clinical practice.

The objective of the present study is to examine the diagnostic accuracy of the biomarker tests in the identification of patients with csPCa.

## 2 Evidence acquisition

A systematic review (SR) of the literature was carried out following the Cochrane Collaboration methodology [9] with reporting in accordance with the Preferred Reporting Items for Systematic Reviews and Meta-analyses (PRISMA) guidelines [10]. The prespecified protocol for this review was registered in PROSPERO (registration number CRD42021240638).

### 2.1 Data sources and searches

The following electronic databases were searched (from 2010 to March 1, 2021): Medline (Ovid platform) and EMBASE (Elsevier interface). The search strategy included both controlled vocabulary and text-word terms related to PCa and molecular biomarker. Searches were limited to the English and Spanish languages. The complete search strategy is available in Supplementary Table 1. We also examined the reference lists of included articles and, through search in Google Scholar, articles that referenced the included studies.

### 2.2 Selection criteria and study selection

Studies were eligible for inclusion if they fulfilled the following criteria:

1. Design: randomized or nonrandomized clinical trials (RCTs or non-RCTs) were eligible for inclusion. In the absence of such designs, cohort and case-control studies that performed an evaluation of the diagnostic validity of the tests were considered.
2. Population: adult men (≥18 yr) with clinical factors that suggested csPCa comprised the study population. Studies with a heterogeneous group of patients (eg, patients with suspected PCa, either iPCa or csPCa) were included only if the results for patients meeting the inclusion criteria were reported separately*.*
3. Index tests: any blood or urine test based on biomarkers aimed at distinguishing csPCa from iPCa was performed.
4. Reference standard: it included alternative tests, biopsy, magnetic resonance, or usual care.
5. Target conditions: csPCa (Gleason score ≥7, International Society of Urological Pathology ≥2, and intermediate- or high-risk localized PCa using the D'Amico Classification System for PCa or according to the European Association of Urology).
6. Outcomes: studies that reported any of the following outcome measures were included: cancer-specific survival, metastasis, change in treatment decisions, and adverse effects. In diagnostic performance studies, measures of sensitivity, specificity, and area under the curve (AUC) with 95% confidence intervals (CIs) were also included, if available.
7. Language: only studies published in English or Spanish were included.
8. Publication type: only full original publications were considered.
9. Date of publication: due to the fact that biomarker-based tests have emerged in the last decade, only studies published from 2010 were considered.

Two reviewers **(**D.I.-V. and A.A.C.**)** screened retrieved references independently and in duplicate, starting with titles and abstracts. The full texts of all articles deemed potentially relevant were then screened to confirm eligibility. Disagreements between the reviewers were checked by a third reviewer (T.P.-S.).

### 2.3 Data extraction process and risk of bias assessment

Data extraction and risk of bias (RoB) assessment were also conducted independently and in duplicate. Discrepancies were discussed and, when no consensus was reached, a third reviewer was consulted. Data extracted include general information, study design, sample characteristics, test details (biomarker and cutoff point), reference standard, and results.

RoB was assessed using either the Cochrane Risk of Bias tools for RCT (RoB 2.0) [11], or the Quality Assessment of Diagnostic Accuracy Studies-2 (QUADAS-2) revised tool [12].

### 2.4 Assessment of publication bias

Potential publication bias was explored by constructing the Deeks asymmetry graphs and computing the Egger test [13], with the significance level set at 0.05, using metafunnel and metabias commands, respectively, in STATA version 16.

### 2.5 Data synthesis

We built 2 × 2 tables summarizing true positive (TP), false positive, true negative, and false negative (FN) values to calculate sensitivity and specificity for detecting csPCa. Review Manager (RevMan, version 5.4.1., 2020; The Nordic Cochrane Center, The Cochrane Collaboration, Copenhagen, Denmark) was used to show the sensitivity and specificity measurements at the study level. Pooled estimates with 95% CIs were performed by bivariant random-effect meta-analyses using the midas command in STATA version 16 [14]. A continuity correction was used in trials that reported zero cells in a 2 × 2 table (eg, when TP or FN is zero). Heterogeneity was assessed by visually analyzing forest plots and through the Higgins I^2^ statistic [15]. Several sources of heterogeneity were anticipated, including the type of diagnostic test, cutoff point, definition of csPCa, and ethnic origin. When reported in studies, the effect using a subgroup analysis was explored.

### 2.6 Certainty of evidence assessment

An assessment of the certainty of evidence per outcome was performed for tests included in the meta-analysis, based on the Grading of Recommendations Assessment, Development, and Evaluation (GRADE) approach. We developed evidence profiles and rated the overall certainty of evidence as high, moderate, low, or very low [16].

## 3 Evidence synthesis

**Fig. 1:**
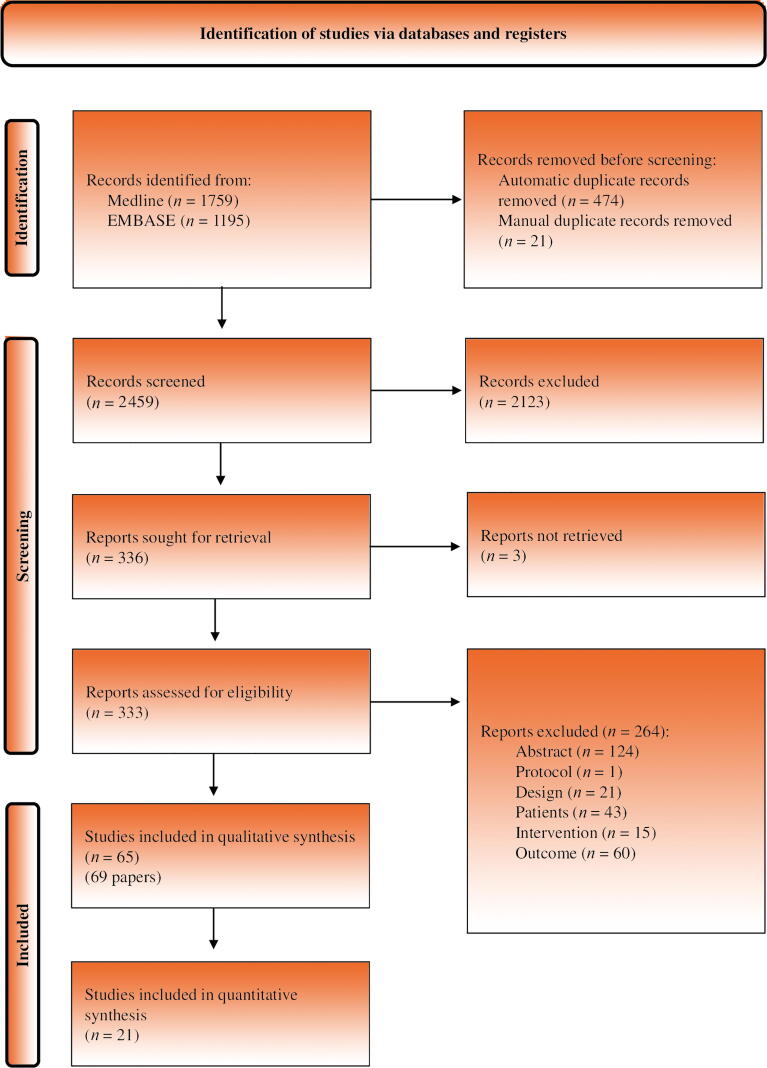
PRISMA flow chart detailing the screening process. PRISMA = Preferred Reporting Items for Systematic Reviews and Meta-analyses.

The results of the literature search and study selection process are shown in Figure 1. Our search identified 2954 references, of which 336 studies were selected for full text assessment. Three of these could not be retrieved [17], [18], [19]. Sixty-five studies [20], [21], [22], [23], [24], [25], [26], [27], [28], [29], [30], [31], [32], [33], [34], [35], [36], [37], [38], [39], [40], [41], [42], [43], [44], [45], [46], [47], [48], [49], [50], [51], [52], [53], [54], [55], [56], [57], [58], [59], [60], [61], [62], [63], [64], [65], [66], [67], [68], [69], [70], [71], [72], [73], [74], [75], [76], [77], [78], [79], [80], [81], [82], [83], [84], reported in 69 articles [20], [21], [22], [23], [24], [25], [26], [27], [28], [29], [30], [31], [32], [33], [34], [35], [36], [37], [38], [39], [40], [41], [42], [43], [44], [45], [46], [47], [48], [49], [50], [51], [52], [53], [54], [55], [56], [57], [58], [59], [60], [61], [62], [63], [64], [65], [66], [67], [68], [69], [70], [71], [72], [73], [74], [75], [76], [77], [78], [79], [80], [81], [82], [83], [84], [85], [86], [87], [88], finally fulfilled the pre-established selection criteria. The list of studies excluded at the full-text level and the reasons for exclusion are provided in Supplementary Table 2.

### 3.1 Characteristics of included studies

The main characteristics of the selected studies are summarized in Table 1, Table 2.

**Table 1:** Main characteristics of included studies

Study	Funded^a^	Design	Biomarker	Test	Biopsy	Outcome		
Country						SE SP	PPV NPV	AUC
Abrate (2015) [74] France, Germany, Italy, Spain, and UK	Y	Nested case-control	PHI	Beckman Coulter	≥12-cylinder TRUS guided	N	N	Y
Babajide (2021) [22] USA	N	Case-control	PHI	Beckman Coulter	NR	Y	Y	Y
Barisiene (2020) [63] Lithuania	Y	Prospective	PHI PHID	Beckman Coulter	NR	Y	Y	Y
Bertok (2020) [32] Austria	N	Retrospective	PHI	Beckman Coulter	TRUS guided or systematic	N	N	Y
Boegemann (2016) [75] Germany and France	N	Ambispective	PHI	Beckman Coulter	8-cylinder TRUS guided	N	N	Y
Busetto (2020) [57] Italy	N	Prospective	HOXC6 and DLX1	SelectMDx	10–12-cylinder TRUS guided	Y	Y	Y
Cao (2018) [23] USA	N	Retrospective	PCA3	Progensa PCA3	12 cylinder	Y	N	Y
Catalona (2011) [24] USA	Y	Case-control	PHI	Beckman Coulter	≥12 cylinder	Y	Y	Y
Chiu (2016) [43] China	Y	Prospective	PHI	Beckman Coulter	≥10 cylinder TRUS guided	N	Y	N
Chiu (2016) [54] China	Y	Prospective	PHI	Beckman Coulter	≥10 cylinder TRUS guided	N	Y	Y
Chiu (2019) [77] France, Germany, Singapore, China, and Taiwan	Y	Prospective	PHI	Beckman Coulter	TRUS guided	Y	Y	N
Choi (2020) [61] South Korea	NR	Retrospective	PHI	Beckman Coulter	Transrectal or transperineal	Y	Y	N
De La Calle (2015) [25] USA	N	Prospective	PHI	Beckman Coulter	TRUS guided	Y	Y	Y
Druskin (2018) [26] USA	N	Retrospective	PHID	Beckman Coulter	TRUS guided	Y	Y	Y
Falagario (2021) [27] USA	NR	Retrospective	Total PSA, free PSA, intact PSA, and HK2	4Kscore test	mpMRI fusion and 12-cylinder systematic	N	N	Y
Fan (2019) [73] Taiwan	N	Prospective	PHI	Beckman Coulter	12-cylinder bilateral TRUS guided	Y	Y	N
Filella (2014) [91] Spain	N	Ambispective	PHI	Beckman Coulter	NR	N	N	Y
Foj (2020) [46] Spain	N	Retrospective	PHI	Beckman Coulter	10-cylinder bilateral TRUS guided	N	N	Y
Foley (2016) [87] Ireland	N	Retrospective analysis	PHI	Beckman Coulter	12-cylinder TRUS guided	N	N	Y
Furuya (2017) [60] Japan	NR	Retrospective	PHI + MRI	Beckman Coulter	≥16-cylinder transperineal ultrasound guided	Y	Y	Y
Guazzoni (2011) [58] Italy	Y	Prospective	PHI	Beckman Coulter	10–12-cylinder TRUS guided	N	N	Y
Haese (2019) [78] The Netherlands, France, and Germany	N	Retrospective	HOXC6 and DLX1 + mpMRI	SelectMDx	10–12-cylinder TRUS guided	Y	Y	Y
Hansen (2013) [28] USA	Y	Retrospective	PCA3	Progensa PCA3	≥10-cylinder TRUS-TRUS guided	N	Y	Y
Hsieh (2020) [72] Taiwan	N	Prospective	PHI	Beckman Coulter	12-cylinder TRUS guided	Y	Y	Y
Kim (2020) [64] UK	Y	Prospective	PHI	Beckman Coulter	Transrectal or transperineal	Y	Y	N
Kotova (2020) [66] Russia	N	Prospective	PCA3	NR	NR	Y	Y	Y
Lazzeri (2013) [92] Italy, Germany, France, Spain, and UK	Y	Prospective	PHI	Beckman Coulter	≥12-cylinder TRUS guided	N	N	Y
Lazzeri (2016) [80] Italy, France, Spain, Germany, and UK	Y	Nested case-control	PHI	Beckman Coulter	≥12-cylinder TRUS guided	N	N	Y
Leyten (2015) [53] The Netherlands	Y	Retrospective	PCA3	Progensa PCA3	≥10-cylinder TRUS guided	N	N	Y
Loeb (2015) [29] USA	Y	Prospective	PHI	Beckman Coulter	≥10 cylinder	Y	Y	N
Loeb (2017) [30] USA	Y	Prospective	PHI	Beckman Coulter	≥6 cylinder	Y	Y	Y
McKiernan (2016) [31] USA	Y	Prospective	ERG and PCA3	ExoDx Prostate IntelliScore urine exosome assay	NR	Y	Y	Y
Mearini (2014) [59] Italy	NR	Prospective	PHI	Beckman Coulter	TRUS guided	N	N	Y
			PHID					
Morote (2016) [47] Spain	NR	Prospective	PHI	Beckman Coulter	≥12-cylinder TRUS guided	Y	Y	Y
Mortezavi (2021) [70] Sweden	N	Prospective	Clinical variables, total PSA, free PSA, HK2, MSMB, and HOXB13	Stockholm3 test	TRUS guided and mpMRI fusion and/or 12-cylinder systematic	N	N	Y
Na (2014) [76] China	Y	Prospective	PHI	Beckman Coulter	10-cylinder TRUS guided	N	N	Y
Na (2017) [65] China	N	Prospective	PHI	Beckman Coulter	10–14-cylinder TRUS guided	Y	Y	N
Nordström (2015) [93] Sweden	N	Case-control	PHI Total PSA, free PSA, intact PSA, and HK2	Beckman Coulter 4Kscore test	10–12-cylinder TRUS guided	N	N	Y
Nordström (2021) [69] Sweden	N	Retrospective analysis	Clinical variables, total PSA, free PSA, HK2, MSMB, HOXB13, and PHID	Stockholm3 test	10–12 cylinder	N	N	Y
Nygård (2016) [95] Norway	N	Prospective	PCA3	Progensa PCA3	Extended 10 cylinder	Y	Y	Y
O'Malley (2017) [33] USA	N	Prospective	PCA3 TMPRSS2 and ERG	Progensa PCA3 TMPRSS2:ERG	TRUS guided	N	N	Y
Park (2018) [62] South Korea	N	Prospective	PHI	Beckman Coulter	TRUS guided	N	N	Y
Punnen (2018) [34] USA	Y	Prospective	Total PSA, free PSA, intact PSA, and HK2	4Kscore test	≥10-cylinder TRUS guided	N	N	Y
Roumiguié (2020) [49] France	N	Retrospective	HOXC6 and DLX1	SelectMDx	TRUS guided and mpMRI fusion	Y	Y	Y
Ruffion (2013) [50] France	NR	Prospective	PCA3	Progensa PCA3	TRUS guided	N	N	Y
Ruffion (2014) [51] France	NR	Prospective	PCA3	Progensa PCA3	TRUS guided	Y	Y	N
Sanchís-Bonet (2018) [48] Spain	N	Prospective	PHI	Beckman Coulter	NR	N	N	Y
Sanda (2017) [35] USA	Y	Retrospective	PCA3	Progensa PCA3	12-cylinder TRUS guided	Y	Y	N
Schulze (2020) [20] Germany	N	Prospective	PHI PHID	Beckman Coulter	10–14 cylinder	Y	Y	N
Seisen (2015) [52] France	Y	Prospective	PHI PCA3	Beckman Coulter Progensa PCA3	≥12-cylinder TRUS guided	Y	Y	Y
Shore (2019) [36] USA	N	Retrospective	HOXC6 and DLX1	SelectMDx	10–12-cylinder TRUS guided or mpMRI fusion	Y	N	N
Steuber (2022) [21] Germany	Y	Prospective	Thrombospondin-1, cathepsin D, total PSA, free PSA, and patient age	Proclarix test	10–12-cylinder TRUS guided and mpMRI fusion	Y	Y	N
Tan (2017) [67] Singapore	N	Prospective	PHI	Beckman Coulter	≥12-cylinder TRUS guided	Y	Y	Y
Tomlins (2016) [37] USA	Y	Prospective	PCA3, T2:ERG, and serum PSA level TMPRSS2 and ERG	MyProstateScore TMPRSS2:ERG	TRUS guided	N	N	Y
Tosoian (2017) [94] USA	N	Prospective	PHI	Beckman Coulter	NR	N	N	Y
Tosoian (2017) [38] USA	N	Prospective	PHI PHID	Beckman Coulter	NR	Y	Y	Y
Tosoian (2021) [40] USA	N	Prospective	PCA3 PCA3, TMPRSS2, and serum PSA level	MyProstateScore	TRUS guided	Y	Y	N
Van Neste (2016) [55] The Netherlands		Prospective	PCA3	Prototype kit	10-cylinder TRUS guided	N	N	Y
Wang (2017) [82] China	N	Prospective	PCA3	Progensa PCA3	10–12-cylinder TRUS guided	N	N	Y
Wei (2014) [41] USA	Y	Prospective	PCA3	Progensa PCA3	TRUS guided	N	N	Y
Woo (2020) [81] USA and Spain	N	Prospective	PCA3 PCA3 and T2:ERG	NR	NR	N	N	Y
Wu (2019) [89] China	N	Prospective	PHI	Beckman Coulter	NR	N	N	Y
Wysock (2020) [42] USA	NR	Prospective	Total PSA, free PSA, intact PSA, and HK2 HOXC6 and DLX1	4Kscore test SelectMDx	TRUS guided and mpMRI fusion and/or 12-cylinder systematic	Y	Y	Y
Yu (2016) [83] China	Y	Prospective	PHI	Beckman Coulter	10-cylinder TRUS guided	N	N	Y
Zappala (2017) [44] USA	NR	Prospective	Total PSA, free PSA, intact PSA, and HK2	4Kscore test	≥10-cylinder TRUS guided	N	N	Y

AUC = area under the curve; DLX1 = distal-less homeobox 1; HK2 = human kallikrein 2; HOXC6 = homeobox C6; HOXC13 = homeobox C13; mpMRI = multiparametric MRI; MRI = magnetic resonance imaging; MSMB = microseminoprotein beta; N = no; NPV = negative predictive value; NR = not reported; PCA3 = prostate cancer antigen 3; PHI = Prostate Health Index; PHID = PHI density; PPV = positive predictive value; PSA = prostate-specific antigen; SE = sensitivity; SP = specificity; TRUS = transrectal ultrasound; Y = yes.

a Funded by industry.

**Table 2:** Selection criteria and clinical and sociodemographic characteristics of the participants in the included studies

Study	*N*	*N* PCa	Clinically significant PCa		Age			Inclusion criteria	Exclusion criteria	Ethnicity (%)	PSA (ng/ml)
			*N*	Definition	Mean/median	SD/IQR	Range				
Abrate (2015) [74]	142	65	44	Gleason ≥7	65.40	7.52^a^	NR	1. >45 yr 2. With or without suspect DRE 3. Obese (BMI ≥30)	1. Bacterial prostatitis (3 mo prior) or undergoing previous transurethral endoscopic surgery 2. Chronic renal failure, marked alterations in blood proteins (normal plasma range 6–8 g/dl), hemophiliacs, or those who previously received multiple transfusions	NR	6.80 (4.40–10.10)^b^
Babajide (2021) [22]	293	158	52	ISUP grade ≥2	NR	NR	40–79	NR	1. Background of PCa 2. Treated with 5-alpha reductase inhibitors 3. Suspicious DRE 4. PSA >10.00 ng/ml 5. Previous prostate biopsy	African Americans (100)	NR
Barisiene (2020) [63]	210	112	40 81	ISUP grade ≥2 Epstein classification	63	7.09	NR	1. >50 yr 2. Elevated PSA (2.50–10.00 ng/ml) 3. Negative DRE	1. Background of PCa 2. History of endoscopic or open prostate surgery 3. Previous prostate biopsy (3 mo) 4. Treated with 5-alpha reductase inhibitors (previous 3 mo) 5. Acute prostatitis or UTI	NR	4.32 ± 1.85^c^
Bertok (2020) [32]	140	70	42	Gleason ≥7	62.15	NR	NR	1. Elevated PSA 2. TRUS-guided prostate biopsy	NR	NR	2.00–10.00^d^
Boegemann (2016) [75]	769	347	111	Gleason ≥7	59	NR	39–65	1. Elevated PSA (1.60–8.00 ng/ml) 2. With or without suspect DRE	1. Acute or chronic prostatitis 2. UTI 3. Treated with 5-alpha reductase inhibitors (previous 6 mo) 4. Background of PCa	NR	4.60 (4.40–4.71)^b^
Busetto (2020) [57]	52	17	7	ISUP grade ≥2	64	8.70	44–79	1. Timed to biopsy by elevated PSA (>3 ng/ml) or suspicious DRE	1. Background of PCa or another neoplasm under active treatment 2. Medical treatment known to affect serum PSA levels and the prostate gland 3. Invasive treatment for BPH 4. Previous prostate biopsy	NR	6.80 ± 3.90 (1.00–19.90)^e^
Cao (2018) [23]	271	77	52	Gleason ≥7	63	NR	59–68	NR	1. Biopsy results not available	African Americans (14.4)	NR
Catalona (2011) [24]	892	430	139	Gleason ≥7	62.80	7.00	50–84	1. No history of PCa 2. Negative DRE 3. Elevated PSA (1.50–11.00 ng/ml) 4. Prostate biopsy of ≥6 casts within 6 mo after blood collection	1. PSA outside 2.00–10.00 ng/ml 2. Medical treatment known to affect serum PSA levels 3. Previous interventions such as transurethral resection of the prostate 4. Acute prostatitis 5. UTI 6. Blood collection or biopsy at inappropriate time interval 7. Previous androgen replacement therapy	Caucasians (81) African Americans (5) Other ethnicities (4) Unknown (10)	5.40 ± 1.90 (2.00–10.00)^e^
Chiu (2016) [43]	312	53	24	Gleason ≥7	68.10	6.20	51–82	1. Elevated PSA (10.00–20.00 ng/ml) and negative DRE 2. TRUS-guided prostate biopsy and prospective blood sample collection	NR	Asians	13.27 ± 2.71 (9.95–20.01)^e^
Chiu (2016) [54]	569	62	16	Gleason ≥7	66	NR	61–71	1. Elevated PSA (4.00–10.00 ng/ml) and negative DRE 2. With or without lower urinary tract symptoms 3. Before the prostate biopsy	1. Background of PCa 2. Any suspicious rectal finding 3. Withdrawal therapy or 5-alpha reductase inhibitors	Asians	6.73 (5.64–8.03)^b^
Chiu (2019) [77]	503 1149	262 151	115 66	Gleason ≥7 Gleason ≥7	63 65	NR NR	58–68 61–71	1. TRUS-guided prostate biopsy from 10 to 12 cylinders 2. Elevated PSA (2.00–20.00 ng/ml)	NR	Caucasians Asians	2.00–10.00^d^ 2.00–10.00^d^
Choi (2020) [61]	114	37	28	Gleason ≥7	62.81^a^	7.87 a	NR	1. Biopsied with previous PSA and PHI 2. Elevated PSA (2.50–10.00 ng/ml)	NR	NR	NR
De La Calle (2015) [25]	561	233	114	Gleason ≥7	62.10	8.30	38–87	1. Biopsy and blood draw completed	1. Background of PCa 2. Positive previous prostate biopsy	Caucasians (85.2) African Americans (10.2) Hispanic (4.5) Other ethnicities (2.7)	6.50 ± 12.20 (0.30–232.80)^e^
	395	205	122	Gleason ≥7	62.80	8.60	33–85			Caucasians (87.8) Afro-Americans (8.4) Hispanic (3.8) Other ethnicities (5.8)	5.90 ± 10.90 (0.30–208.80)^e^
Druskin (2018) [26]	241	NR	91	ISUP grade1 detected on >2 cylinders or >50% of any core ISUP grade ≥2	65	NR	59.3–70.8	1. Elevated PSA (<10.00 ng/ml) and negative DRE	1. Suspicious DRE	African Americans (11.6)	7.00 (4.90–10.20)^b^
			71	ISUP grade ≥2							
			38	ISUP grade ≥3							
Falagario (2021) [27]	256	153	94	ISUP grade ≥2	66	NR	60–70.9	NR	NR	NR	5.90 (4.20–8.50)^b^
Fan (2019) [73]	307	95	52	Gleason ≥7	66	NR	57.67–74.33	1. Timed to biopsy by PSA 4.00–10.00 ng/ml with or without a suspicious DRE or PSA <4 ng/ml and a suspicious DRE	1. Acute prostatitis or UTI 2.Treated with 5-alpha reductase inhibitors (previous 3 mo) 3. Previous prostate biopsy 4. Previous transurethral resection of the prostate	Asians	4.00–10.00^d^
Filella (2014) [91]	354	175	80	Gleason ≥7	68	NR	38–88	1. Elevated PSA or suspicious DRE	1. Medical treatment known to affect serum PSA levels 2. Acute prostatitis or UTI 3. Invasive treatment for BPH	NR (Spanish)	6.17 (1.92–36.90)^b^
Foj (2020) [46]	276	151	80	D’Amico classification: intermediate and high risk	66.65	7.29	NR	1. Timed to biopsy by elevated PSA or suspicious DRE	1. Medical treatment known to affect serum PSA levels 2. Chronic kidney failure 3. Acute prostatitis or UTI 4. Invasive treatment for BPH	NR (Spanish)	6.14 (0.50–36.90)^b^
Foley (2016) [87]	250	112	77	Gleason ≥7	63.87	NR	NR	1. Availability of a biobank serum sample prior to biopsy	1. <40 yr 2. Background of PCa	NR	6.40 (0.50–1400.00)^b^
Furuya (2017) [60]	50	33	21	Gleason ≥7	68.50	NR	53–82	1. Elevated PSA (2.00–10.0 ng/ml) 2. MRI before biopsy	1. Bacterial prostatitis (previous 3 mo) 2. Previous endoscopic prostate surgery 3. Treated with 5-alpha inhibitors, antiandrogens, or luteinizing hormone-releasing hormone analogs	NR	6.92 ± 1.69 (3.74–9.96)^e^
Guazzoni (2011) [58]	268	107	52	Gleason ≥7	63.30	8.20	NR	1. Elevated PSA (2.00–10.00 ng/ml) 2. Negative DRE 3. Informed consent	1. Acute or chronic bacterial prostatitis (previous 3 mo) 2. Invasive treatment for BPH 3. Medical treatment known to affect serum PSA levels	NR	5.70 (2.00–9.90)^b^
Haese (2019) [78]	1039 916	521 467	282 258	ISUP grade ≥2	64 65	NR NR	59–69 60–70	NR	1. Background of PCa 2. Medical treatment known to affect serum PSA levels (previous 6 mo) 3. Invasive treatment for BPH (previous 6 months)	NR	6.20 (4.60–9.40)^b^ 6.40 (4.50–9.20)^b^
Hansen (2013) [28]	692	318	137	Gleason ≥7	64	NR	58–69	1. Suspicious DRE 2. 10-cylinder prostate biopsy 3. Elevated PSA (2.50–10.00 ng/ml) 4. Informed consent	1. UTI 2. Medical treatment known to affect serum PSA levels 3. Background of PCa 4. Invasive treatment for BPH 5. Lack of information on prostate volume	NR	5.20 (4.30–7.20)^b^
Hsieh (2020) [72]	102	39	24	Grade group ≥2	65.50	NR	60–70	1. >40 yr 2. Scheduled to biopsy for suspected PCa due to elevated PSA (PSA >4.00 ng/ml) and/or suspicious DRE	1. Background of PCa 2. Bacterial prostatitis (previous 3 mo) 3. Treated with 5-alpha reductase inhibitors 4. Inability or unwillingness to sign the informed consent	Asians	7.78 (6.12–11.80)^b^
Kim (2020) [64]	545	349	258 174	ISUP grade ≥2 ISUP grade ≥3	NR	NR	NR	Elevated PSA	1. Previous prostate biopsy 2. Pelvic metal interfering with mpMRI quality 3. No biopsy was performed after mpMRI	NR	8.00 (6.00–13.00)^b^
Kotova (2020) [66]	128	61	33	Gleason ≥7	66	NR	44–88	1. Elevated PSA (≥2.00 ng/ml) 2. 40–90 yr	1. Previous treatment with antiandrogens 2. Bladder catheterization/cystostomy	NR	8 (2.06–76.80)^b^
Lazzeri (2013) [92]	646	264	139	Gleason ≥7	64.20	7.50	NR	1. >45 yr 2. Suspicious DRE 3. Elevated PSA (2.00–10.00 ng/ml)	1. Bacterial prostatitis (previous 3 mo) 2. Previous endoscopic surgery 3. Previous prostate biopsy 4. Treated with dutasteride or finasteride 5. Chronic renal failure, marked abnormalities in blood proteins (normal plasma range 6–8 g/dl), hemophiliacs, or those who previously received multiple transfusions	Caucasians (96)	5.80 (4.30–7.70)^b^
Lazzeri (2016) [80]	262	136	106	Gleason ≥7	67.30	8.10	NR	1. >45 yr 2. PSA 4.00–10.00 ng/ml 3. With or without suspect DRE 4. With or without previous negative biopsy	1. Bacterial prostatitis 2. Previous prostate endoscopic surgery 3. Treated with dutasteride or finasteride 4. Chronic renal failure 5. Marked alterations in blood proteins (normal plasma range: 6–8 g/d), hemophiliacs, or with multiple previous transfusions	NR	15.30 (11.90–22.50)^b^
Leyten (2015) [53]	358	157	93	Gleason ≥7	65	NR	44–86	NR	1. Background of PCa 2. Medical treatment known to affect serum PSA levels (previous 6 mo) 3. Prostate biopsies (previous 3 mo) 4. Invasive treatment for BPH (previous 6 mo)	NR	NR
Loeb (2015) [29]	658	324	160 109	Epstein classification Gleason ≥7	63	NR	50–84	1. >50 yr 2. PSA 2.00–10.00 ng/ml 3. Negative DRE	NR	Caucasians (81.2) African Americans (5.3) Other ethnicities (13.5)	NR
Loeb (2017) [30]	728	334	118	Gleason ≥7	62.80	6.90	NR	1. Elevated PSA (2.00–10.00 ng/ml) and negative DRE 2. Prostate biopsy with ≥6 casts <6 mo after blood collection	1. Prostate surgery 2. UTI 3. Medical treatment known to affect serum PSA levels (eg, 5-alpha reductase inhibitors)	Caucasians (83) African Americans (5) Other ethnicities (12)	5.40 ± 1.90 (2.00–1000)^e^
McKiernan (2016) [31]	255	120	78	Gleason ≥7	62	NR	50–79	1. >50 yr 2. Timed to biopsy (initial or repeated) by elevated PSA (2.00–20.00 ng/ml) or suspicious DRE	1. Invasive treatment for BPH (previous 6 mo) 2. Medical treatment known to affect serum PSA levels (previous 3–6 mo)	Caucasians (70) African Americans (19) Hispanic (6) Asians (2) Other ethnicities (2)	4.95 (2.00–10.00)^b^
	519	250	148		63	NR	50–90		1. History of invasive treatment for benign prostate disease in the previous 6 mo 2. Medical treatment known to affect serum PSA levels (previous 3–6 mo)	Caucasians (74) African Americans (17) Hispanic (6) Asians (3) Other ethnicities (1)	5.12 (2.00–10.00)^b^
Mearini (2014) [59]	275	86	26	Gleason ≥7	65.40	6.80	NR	1. Elevated PSA (2.00–10.00 ng/ml)	1. Acute or chronic prostatitis 2. Surgery or previous prostate biopsy 3. Medical treatment known to affect serum PSA levels	NR	4.50 (2.00–10.00)^b^
Morote (2016) [47]	183	68	45	Gleason ≥7 (any Gleason 4 pattern on biopsy) or cT3	67	NR	48–75	1. <75 yr 2. Elevated PSA (3.00–10.00 ng/ml) 3. Scheduled to biopsy (initial)	NR	NR (Spanish)	5.10 (3.00–10.00)^b^
Mortezavi (2021) [70]	532	291	194	Gleason ≥7	64	7	NR	1. 45–75 yr 2. No previous diagnosis of PCa 3. Elevated PSA or suspicious DRE	1. Serious diseases such as metastatic cancers, severe cardiovascular disease, or dementia 2. Contraindications to MRI	NR	6.10 (4.10–8.80)^b^
Na (2014) [76]	636	274	158	Gleason 4 + 3 and ≥7	68	NR	25–100	1. PSA >4.00 ng/ml 2. % ratio of fPSA <0.16 3. Density of PSA >0.15 4. Presence of prostate nodules detected by DRE or TRUS	NR	Asians	NR
Na (2017) [65]	1538	618	488	Gleason ≥7	66.95	8.89	NR	1. PSA >10.00 ng/ml 2. PSA >4.00 ng/ after 2–3 mo 3. %fPSA <0.16 when patients had a total level of total PSA >4.00 ng/ml 4. Suspicious lesions detected on DRE or TRUS with any PSA level	NR	Asians	11.42 (7.00–24.04)^b^
Nordström (2015) [93]	531	271	134	Gleason ≥7	NR	NR	NR	1. Elevated PSA (3.00–15.00 ng/ml)	NR	NR	NR
Nordström (2021) [69]	1544	509	213	ISUP grade ≥2	64.20	4.80	50–69	1. Elevated PSA (>3.00 ng/ml)	NR	NR	4.2 ± 2.3
Nygård (2016) [95]	124	70	49	Intermediate and high risk according to the European Association of Urology	65.10^a^	12.37^a^	NR	1. Elevated PSA (3.00–25.00 ng/ml) 2. ≤75 yr 3. No previous biopsies ≤5 yr 4. Susceptible to radical treatment	NR	NR	7.20 (8.30–9.90)^b^
O'Malley (2017) [33]	718	518	194	Gleason ≥7	63	NR	57–68	1. Elevated PSA and/or in progression, <15% free PSA 2. Family history of PCa 3. Previous atypical small acinar proliferation or high-grade intraepithelial neoplasia of the prostate or suspicious for DRE	1. Participación en un ensayo para enfermedad de la próstata 2. Biopsia de próstata (previous 6 mo) y exposición previa a la prueba de PCA3	African Americans (10) Non–African Americans (90)	5.10 (3.80–7.00)^b^
Park (2018) [62]	246	125	29	Gleason ≥7	69.60	8.70	NR	1. Elevated PSA (≥3.50 ng/ml) and negative DRE	1. Background of PCa or other urogenital cancers 2. Previous endoscopic surgery of the prostate, acute or chronic prostatitis (previous 3 mo), or untreated UTI 3. Previous prostate biopsy 4. Treated with dutasteride or finasteride 5. Chronic kidney failure, hemophiliacs, or those who previously received multiple transfusions	NR	7.80 (3.50–387.20)^b^
Punnen (2018) [34]	366	215	131	ISUP grade ≥2	NR	NR	NR	1. Prostate biopsy with ≥10 nuclei	1. Background of PCa 2. DRE within 96 h of phlebotomy 3. Invasive treatment of the prostate 4. Treated with 5-alpha reductase inhibitors (previous 3 mo)	African Americans (56) Caucasians (40) Other ethnicities (4)	NR
Roumiguié (2020) [49]	117	64	24	ISUP grade ≥2	65	63–67	NR	1. Elevated PSA or suspicious DRE	NR	NR	7.00 (6.50–8.00)^b^
Ruffion (2013) [50]	594	276	128	Gleason ≥7	63	NR	58–67	1. >55 yr 2. Informed consent	1. PSA ≥20.00 ng/ml 2. Previous prostate biopsy 3. Medical treatment known to affect serum PSA levels 4. Previous prostate surgery for BPH 5. <55 yr	French	5.90 (4.70–7.90)^b^
Ruffion (2014) [51]	595	274	125	Gleason ≥7	63	58-67	NR	1. Elevated PSA or suspicious DRE or family history of PCa	1. PSA >20.00 ng/ml 2. ≥T2b 3. Previous surgery 4. Treated with 5-alpha reductase inhibitors	NR	5.90 (4.70–7.90)^b^
Sanchís-Bonet (2018) [48]	197	85	44	Gleason ≥7	68	NR	62–71	1. Elevated PSA (2.00–20.00 ng/ml) or suspicious DRE 2. >45 yr	1. Treated with 5-alpha reductase inhibitors (previous 6 mo) 2. UTI or urinary tract manipulation (previous 3 mo)	NR (Spanish)	5.80 (4.40–7.80)^b^
Sanda (2017) [35]	516	254	156	Gleason ≥7	62	NR	33–85	1. Scheduled to biopsy for the first time 2. Informed consent 3. Posturinary samples after rectal examination (DRE) prior to biopsy	1. Background of PCa 2. Previous prostate biopsy 3. Previous prostatectomy 4. Other cancer diagnosis 5. Inability to provide a post-DRE urine sample	Caucasians (81) African Americans (10) Asians (4) Other ethnicities (5)	4.80 (0.30–460.40)^b^
	561	264	148		62		27–86			Caucasians (79) African Americans (14) Asians (2) Other ethnicities (5)	5.30 (0.20–274.90)^b^
Schulze (2020) [20]	122	76	50	Gleason ≥7	65.41	NR	41–81	1. Timed to biopsy due to elevated PSA or in progression or suspicious DRE	NR	NR	9.55 (1.91–82.90)^b^
Seisen (2015) [52]	138	62	39	Epstein classification	NR	NR	NR	1. Elevated PSA (4.00–20.00 ng/ml) or suspicious DRE	1. Treated with 5-alpha reductase inhibitors (previous 3 mo) 2. Invasive treatment for BPH and acute bacterial prostatitis (previous 3 mo) 3. Chronic kidney failure, marked abnormalities in blood proteins, hemophiliacs, or those who previously received multiple transfusions	NR	7.80 (0.70–19.80)^b^
Shore (2019) [36]	80	27	21	ISUP grade ≥2	67	62–72	NR	1. Scheduled to biopsy	NR	Caucasians (90) African Americans (9) Other ethnicities (1)	NR
Steuber (2022) [21]	362	NR	103	ISUP grade ≥2	64 a	7.79 a	NR	1. Elevated PSA (2.00–10.00 ng/ml) 2. Suspicious DRE (≥35 cm^3^)	1. Treated with 5-alpha reductase inhibitors 2. Prostatitis, cistitis u otra alteración en la próstata 3. Previous prostate biopsy	NR	6.12 (4.61–7.65)^b^
Tan (2017) [67]	157	30	19	Gleason ≥7	65.40	6.46	NR	1. 50–75 yr 2. Suspicious DRE 3. Elevated PSA (4.00–10.00 ng/ml)	1. Bacterial prostatitis 2. UTI not treated 3. Previous endoscopic prostate surgery 4. Background of PCa or other urogenital cancers 5. Previous prostate biopsy 6. Treated with dutasteride or finasteride	Asians (99) Other ethnicities (1)	6.71 ± 2.69^c^
Tomlins (2016) [37]	711	NR	NR	Gleason ≥7	NR	NR	NR	1. Scheduled for biopsy and urine evaluation T2: ERG and PCA3	1. Pretreatment for PCa 2. Surgical treatment of the prostate within 6 mo prior to urine collection 3. Prostate biopsy (previous 6 wk)	NR	NR
	1225	518	224		64	58–70		1. Scheduled for biopsy		Caucasians (73) Non-Caucasians (27)	4.70 (3.30–6.50)^b^
Tosoian (2017) [94]	135	75	46	ISUP grade ≥2	64.30	NR	58.90–70.10	1. Elevated PSA, PSA kinetics, and/or abnormal rectal examination	NR	African Americans (36)	NR
Tosoian (2017) [38]	118	47	35	Gleason ≥7	64.20	NR	58.90–71.20	1. Elevated PSA and PSA kinetics	1. Suspicious DRE	African Americans (10.2)	7.40 (4.90–10.50)^b^
Tosoian (2021) [40]	548	262	146	ISUP grade ≥2	62	NR	56–67	NR	NR	African Americans (14)	4.90 (3.70–6.80)^b^
	516	253	156		61		56–67			African Americans (10)	4.80 (3.60–6.30)^b^
	977	435	192		64		57–69			African Americans (7.5)	4.50 (3.10–6.00)^b^
Van Neste (2016) [55]	386	181	90	Gleason ≥7	64.90	60–70	NR	1. Timed to biopsy (initial or repeated) by elevated PSA (3.00 ng/ml), suspicious DRE, or family history of PCa	1. Background of PCa 2. Medical treatment known to affect serum PSA levels 3. Prostate biopsy (previous 3 mo) 4. Invasive treatment for BPH (previous 6 mo)	NR	7.30 (5.20–10.90)^b^
	519	212	109		64.70	60–70					7.40 (5.50–11.10)^b^
Wang (2017) [82]	430	165	NR	Gleason ≥7	66.29^a^	6.53^a^	NR	1. Elevated PSA (≥4.00 ng/ml) or suspicious DRE	1. Another cancer diagnosis 2. Recent instrumentation or catheterization of the urethra 3. Treated with finasteride or hormonal treatment	Asians	Con biopsia positiva y PSA 4.00–10.00 ng/ml: 7.90 (6.30–9.00)^b^ Con biopsia negativa y PSA 4.00–10.00 ng/ml: 7.12 (5.60–8.50)^b^ Con biopsia positiva y PSA >10.00 ng/ml: 28.80 (16.10–59.60)^b^ Con biopsia negativa y PSA >10.00 ng/ml: 13.50 (11.00–19.70)^b^
Wei (2014) [41]	562	264	148	Gleason ≥7	62	8	NR	1. Timed to biopsy (initial) 2. Elevated PSA or in progression, <15% free PSA3 3. Family history of PCa 4. Previous atypical small acinar proliferation or suspicious or high-grade DRE prostate intraepithelial neoplasia	1. Background of PCa 2. Participation in a trial for prostate disease 3. Previous prostate surgery 4. Previous prostate biopsy (previous 6 mo) 5. Previous PCA3	Caucasians (79) African Americans (14) Other ethnicities (7)	7.00 ± 15.00 c
	297	67	26		64	8				Caucasians (82) African Americans (11) Other ethnicities (7)	10.00 ± 10.00^c^
Woo (2020) [81]	52	20	13	Gleason ≥7	67.80	NR	51–83	1. Scheduled to biopsy by elevated PSA or suspicious DRE	NR	Caucasians	8.60 (1.50–39.20)^b^
	165	107	83		66.50		47–82			Caucasians (92.5)	7.90 (0.10–46.80)^b^
Wu (2019) [89]	635	272	207	Gleason ≥7	69	NR	61–76	NR	1. Lack of essential clinical data: age, PSA, %fPSA (free PSA divided by PSA), p2PSA or PV	Asians	13.30 (7.60–31.50)^b^
	1045	449	347		68		62–74		NR		11.70 (7.00–25.70)^b^
Wysock (2020) [42]	50	26	22	ISUP grade ≥2	63	52–74	NR	1. Elevated PSA	1. Previous biopsy	Caucasians (74) African Americans (14) Hispanic (4) Other ethnicities (8)	5.25 (3.80–8.13)^b^
Yu (2016) [83]	261	67	30	Gleason 4 + 3 and ≥8	67	NR	25–91	1. Elevated PSA (>4 ng/ml) 2. % ratio of fPSA <0.16 3. PSAD >0.15 4. Presence of prostate nodules detected by DRE or TRUS	NR	NR	10.67 (0.41–2006.25)^b^
Zappala (2017) [44]	1012	470	231	Gleason ≥7	66	NR	61–72	1. 10-cylinder TRUS-guided prostate biopsy	1. Background of PCa 2. DRE within 96 h prior to phlebotomy 3. Treated with 5-alpha reductase inhibitors (previous 6 mo) 4. Invasive urological procedures that can affect serum PSA levels (previous 6 mo)	Caucasians (87) African Americans (8.5) Hispanic (4) Other ethnicities (0.5)	NR

BMI = body mass index; BPH = benign prostatic hyperplasia; DRE = digital rectal examination; fPSA = free prostate-specific antigen; IQR = interquartile range; ISUP = International Society of Urological Pathology; mpMRI = multiparametric magnetic resonance imaging; MRI = magnetic resonance imaging; NR = not reported; p2PSA = (-2) pro–prostate-specific antigen; PCa = prostate cancer; PCA3 = prostate cancer antigen 3; PHI = Prostate Health Index; PSA = prostate-specific antigen; PSAD = PSA density; PV = prostate volume; T2: ERG = gene fusion between transmembrane serine protease 2 (TMPRSS2) and the transcription factor ERG; SD = standard deviation; TRUS = transrectal ultrasound; UTI = urinary tract infection.

a Own calculation.

b Median (interquartile range).

c Mean ± SD.

d Range.

e Mean ± SD (range).

Only diagnostic performance studies with an observational design were included: 43 prospective, 13 retrospective, two ambispective, two with a retrospective analysis of prospectively collected data, three case-control studies, and two nested case-control studies. Ten studies contained two [25], [31], [35], [37], [41], [55], [78], [81], [89] or three [40] different populations and data were treated as separate studies.

The diagnostic tests analyzed were Prostate Health Index (PHI), a mathematical combination of free and total PSA and the [-2]pro-PSA isoform (Beckman Coulter, Inc.), in 37 studies [20], [22], [24], [25], [29], [30], [32], [38], [43], [46], [47], [48], [52], [54], [58], [59], [60], [61], [62], [64], [65], [67], [72], [73], [74], [75], [76], [77], [80], [83], [87], [89], [90], [91], [92], [93], [94]; Progensa prostate cancer antigen 3 (PCA3; Gen-Probe Incorporated) in 12 studies [23], [28], [41], [50], [51], [52], [53], [55], [66], [81], [82], [95]; PHI density in five studies [20], [26], [38], [59], [90]; SelectMDx in five studies [36], [42], [49], [57], [78]; 4Kscore test (OPKO Health, Inc.) in four studies [27], [34], [42], [44]; MyProstateScore (MLabs) in three studies [37], [40], [81]; TMPRSS2:ERG in two studies [37], [40]; Stockholm3 in two studies [69], [70]; ExoDx Prostate IntelliScore in one study [31]; and Proclarix test in one study [21]. All of these compared the performance of the tests with prostate biopsy (Table 1).

Included studies involved 34 287 men, 14 792 (43.14%) diagnosed with PCa and 7905 (23.06%) with csPCa. Not all studies included PSA-selected patients [23], [25], [78], [84], [87], [27], [34], [35], [36], [37], [40], [53], [74]. The selection criteria and main characteristics of participants are summarized in Table 2.

### 3.2 RoB in included studies

**Fig. 2:**
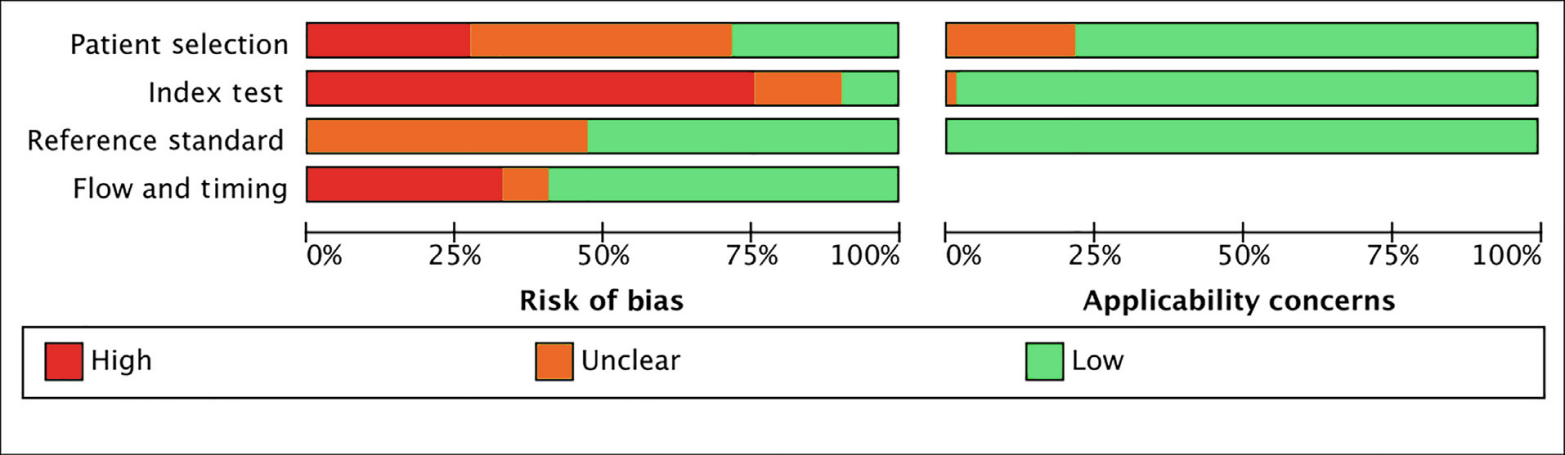

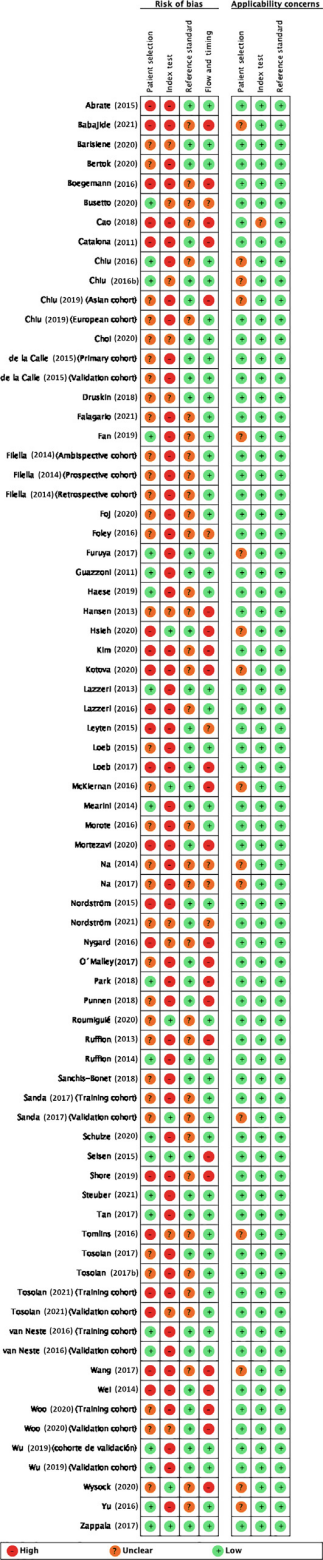
Risk of bias and applicability concerns (QUADAS-2 tool): (A) across studies and (B) within studies. QUADAS-2 = Quality Assessment of Diagnostic Accuracy Studies-2.

The RoB assessment is summarized in Figures 2A and 2B. Out of the 65 studies identified, only one was classified as having a low RoB in all domains. In the remaining studies, the most common methodological concerns involved the domains for the patient selection (19 studies at a high RoB), the index test domain (47 studies and the training cohorts in the studies by Sanda et al. [35], Tosoian et al. [40], and Woo et al. [81] at a high RoB), and the flow and timing (22 studies at a high RoB). The detailed judgments for each domain are available in Supplementary Table 3.

### 3.3 Quality of evidence

The overall quality of the evidence for PHI and SelectMDx was considered low (Supplementary Tables 4 and 5 provide the evidence profiles, respectively).

### 3.4 Synthesis of results

Diagnostic accuracy results of selected studies are listed in Supplementary Table 6.

Out of the 65 included studies, only 21 remained for a quantitative analysis for PHI and SelectMDx [36], [42], [49], [57], [78]. The results of all meta-analyses and subgroups analyses are available in Supplementary Table 7.

#### 3.4.1 Urine tests

##### 3.4.1.1 Progensa PCA3

Cutoff points ranged from 5 to 35. For the cutoff point 15, the test yielded sensitivity ranging between 93% and 99%, and specificity of 37%. The cutoff point 20 showed sensitivity between 89% and 99%, and specificity of 51%. Finally, at the cutoff point 35, the sensitivity ranged between 62% and 71%, while the specificity increased to 59–66%. The AUC ranged from 0.59 to 0.83.

##### 3.4.1.2 SelectMDx test

**Fig. 3:**
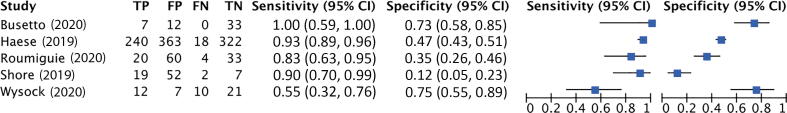

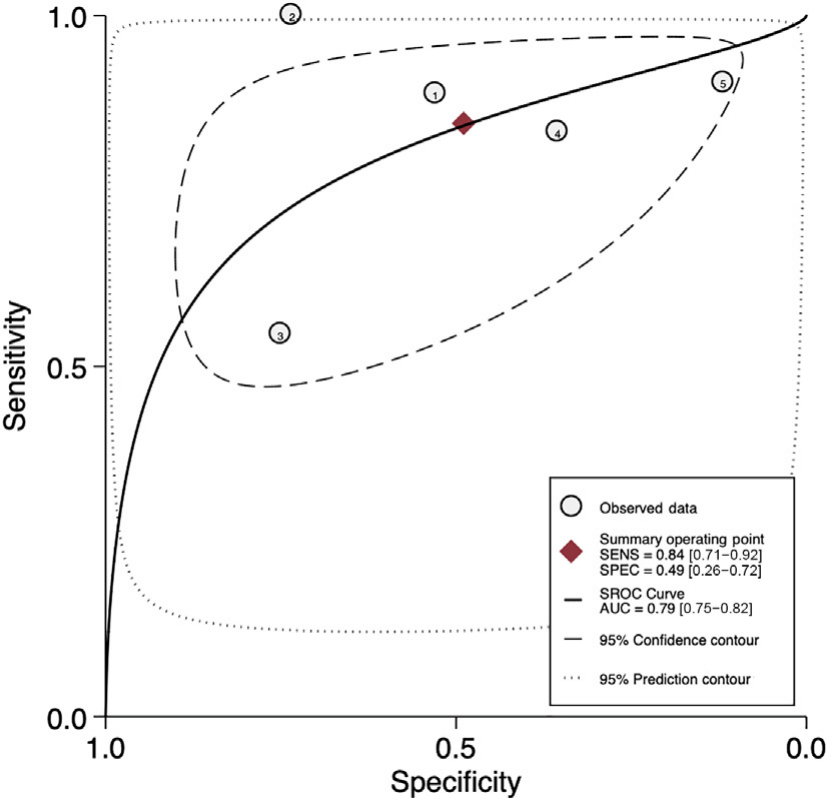
Accuracy of SelectMDx test for csPCa detection. (A) Forest plot of sensitivity and specificity. (B) Summary receiver operating characteristic (SROC) curve. AUC = area under the curve; CI = confidence interval; csPCa = clinically significant prostate cancer; FN = false negative; FP = false positive; SENS = sensitivity; SPEC = specificity; TN = true negative; TP = true positive.

Pooled sensitivity and specificity were 84% (95% CI: 71– 92%; I^2^ = 79.7%; *k* = 5; *n* = 1957) and 49% (95% CI: 26– 72%; I^2^ = 93.9%; *k* = 5; *n* = 1957), respectively (see Fig. 3A). Pooled AUC was 0.79 (95% CI: 0.75–0.82; *k* = 5; *n* = 1957). Figure 3B shows the hierarchic summary ROC plot with 95% CI area and summary point.

##### 3.4.1.3 TMPRSS2:ERG

Reported AUC ranged from 0.64 to 0.75. No data related to the sensitivity and specificity of TMPRSS2:ERG was reported.

##### 3.4.1.4 MyProstateScore

With a cutoff point of ≤10, sensitivity ranged between 96.6% and 97.4%, and specificity between 28.6% and 34.6%. For cutoff points >10, the sensitivity ranged from 95.5% to 96.7%, while the specificity remains between 29% and 33.3% [40]. Reported AUC ranged between 0.60 and 0.80 [37], [81].

##### 3.4.1.5 ExoDx Prostate IntelliScore

The sensitivity and specificity ranged from 91.89% to 97.44% and from 35.7% to 37.25%, respectively. The AUC ranged from 0.73 to 0.78 [31].

#### 3.4.2 Blood tests

##### 3.4.2.1 Prostate Health Index

Only 16 out of 37 selected studies on PHI were included in meta-analyses. Cutoff points ranged from 15 to 55. Subgroup analyses could be conducted by cutoff point and ethnic origin.

###### 3.4.2.1.1 Cutoff point 15–20

**Fig. 4:**
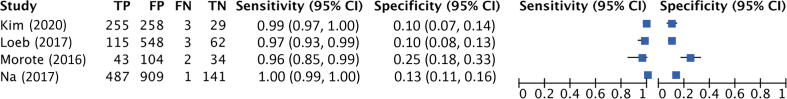

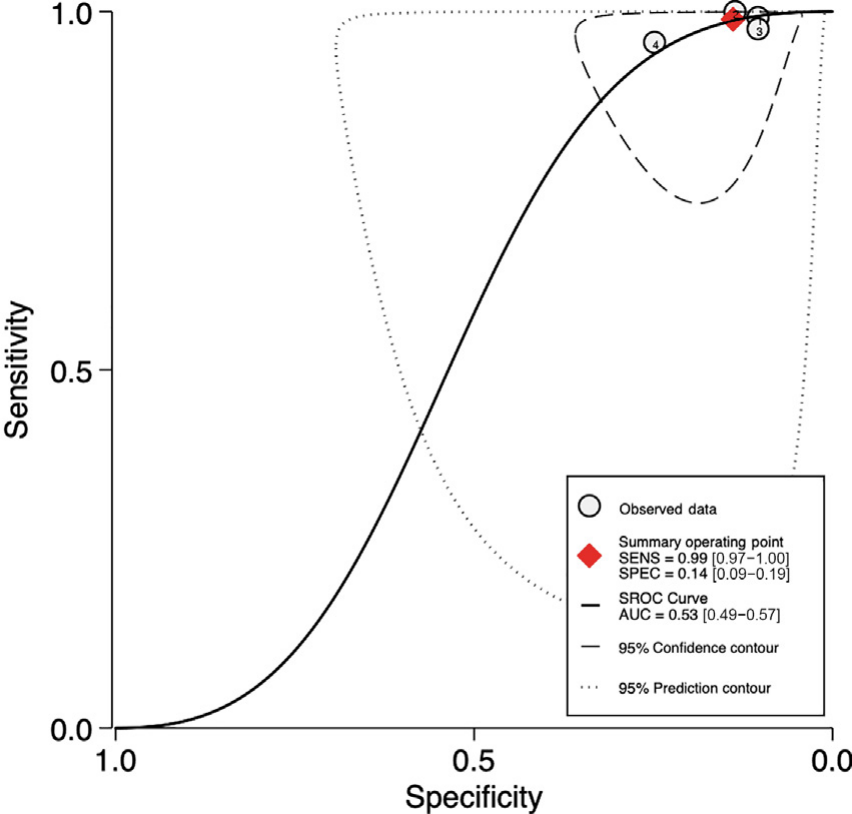

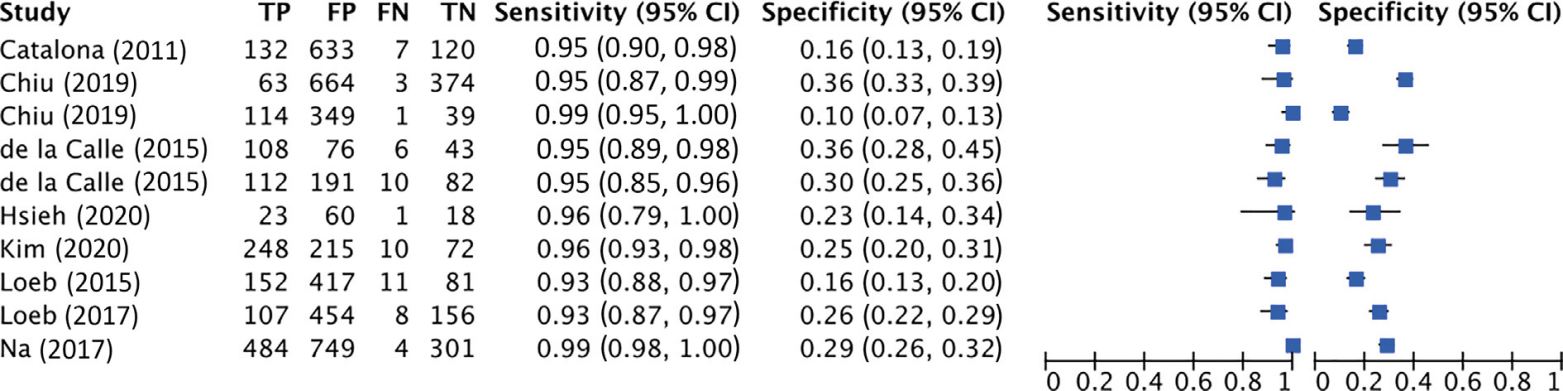

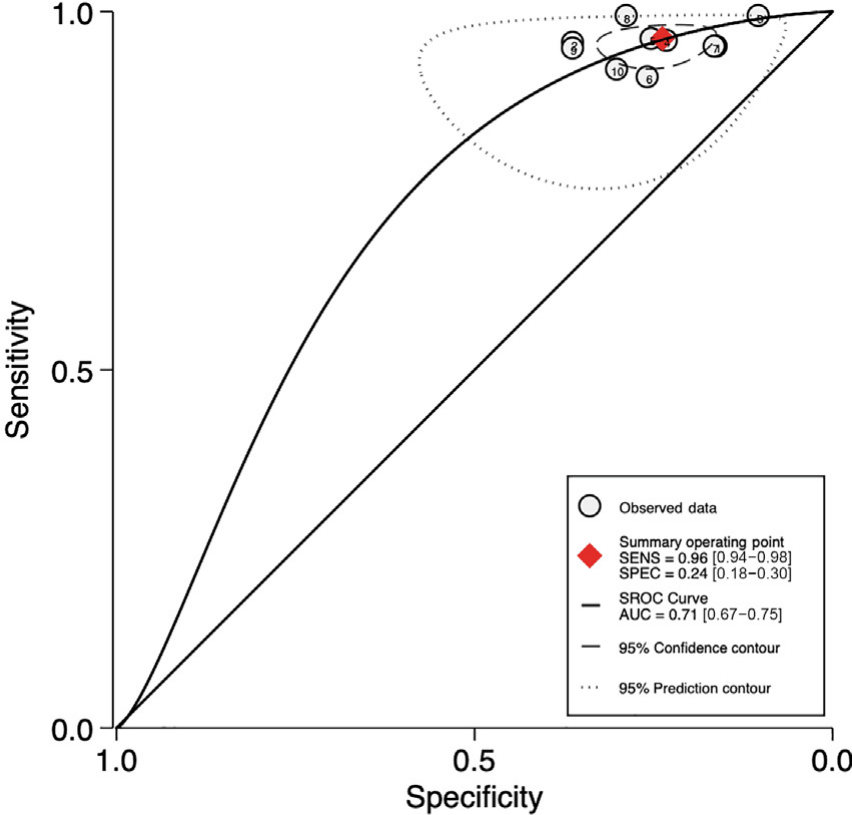

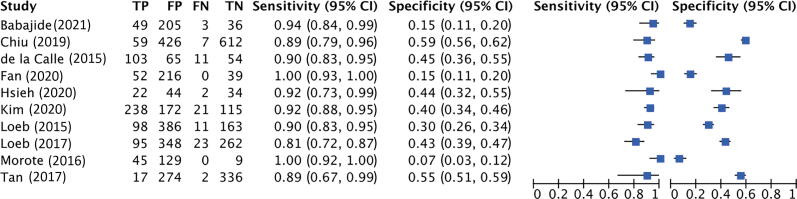

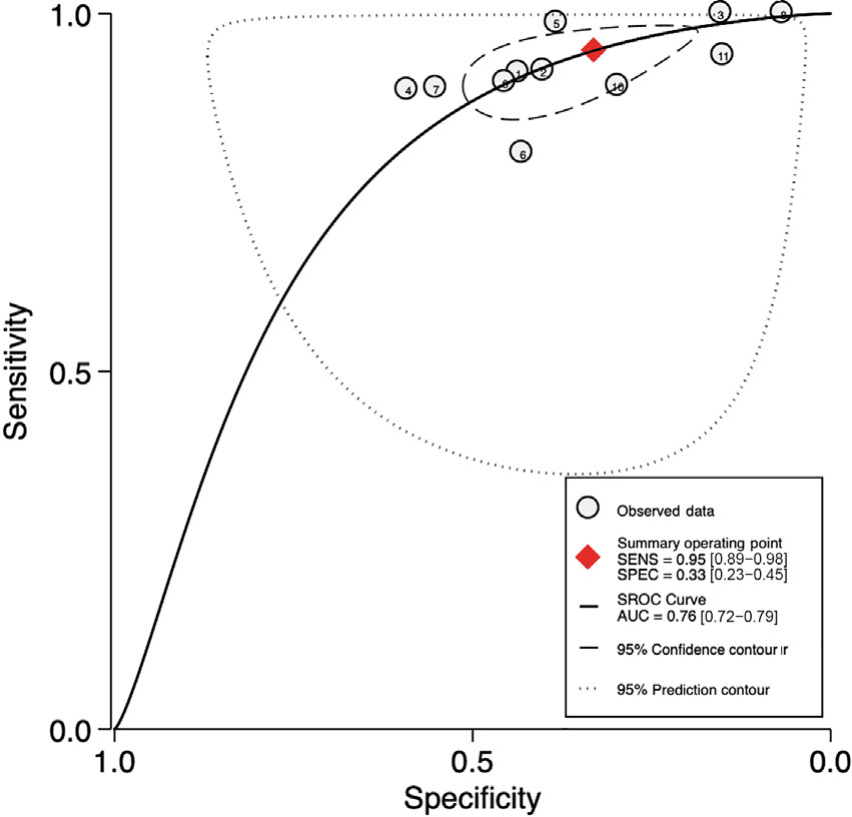
Accuracy of PHI test for csPCa detection. (A) Forest plot of sensitivity and specificity: cutoff point 15–20. (B) Summary receiver operating characteristic (SROC) curve: cutoff point 15–20. (C) Forest plot of sensitivity and specificity: cutoff point 20–25. (D) SROC curve: cutoff point 20–25. (E) Forest plot of sensitivity and specificity: cutoff point 25–30. (F) SROC curve: cutoff point 25–30. AUC = area under the curve; CI = confidence interval; csPCa = clinically significant prostate cancer; FN = false negative; FP = false positive; PHI = Prostate Health Index; SENS = sensitivity; SPEC = specificity; TN = true negative; TP = true positive.

Pooled sensitivity and specificity were 99% (95% CI: 97– 100%; I^2^ = 76.26%; *k* = 4; *n* = 2994) and 14% (95% CI: 9–19%; I^2^ = 87.03%; *k* = 4; *n* = 2994), respectively (Fig. 4A). Pooled AUC was 0.53 (95% CI: 0.49–0.57; *k* = 4; *n* = 2994; Fig. 4B).

PHI showed higher sensitivity and specificity in patients of Asian origin (sensitivity = 100%; 95% CI: 99–100%; specificity = 13%; 95% CI: 4–22%; *k* = 1; *n* = 1556) than in patients of European origin (sensitivity = 98%; 95% CI: 96–100%; specificity = 13%; 95% CI: 8–19%; *k* = 3; *n* = 1428).

###### 3.4.2.1.2 Cutoff point 20–25

Pooled sensitivity and specificity were 96% (95% CI: 94–98%; I^2^ = 73.26%; *k* = 7; *n* = 6698) and 24% (95% CI: 18–30%; I^2^ = 95.57%; *k* = 7; *n* = 6698), respectively (Fig. 4C). The AUC obtained was 0.71 (95% CI: 0.67–0.75; *k* = 7; *n* = 6698; Fig. 4D).

Again, higher accuracy was obtained in Asian patients (sensitivity = 99%; 95% CI: 97–100%; specificity = 30%; 95% CI: 20–39%; *k* = 3; *n* = 2744) than in European patients (sensitivity = 95%; 95% CI: 93–97%; specificity = 21%; 95% CI: 16–26%; *k* = 4; *n* = 3954).

###### 3.4.2.1.3 Cutoff point 25–30

Pooled sensitivity and specificity were 95% (95% CI: 89–98%; I^2^ = 85.72%; *k* = 9; *n* = 6321) and 33% (95% CI: 23–45%; I^2^ = 98.27%; *k* = 9; *n* = 6321), respectively (Fig. 4E). The derived AUC showed an accuracy of 0.76 (95% CI: 0.72–0.79; *k* = 9; *n* = 6321; Fig. 4F).

Sensitivity was higher in Asian patients (98%; 95% CI: 94–100%; *k* = 5; *n* = 3680) than in African-American (94%; 95% CI: 83–100%; *k* = 1; *n* = 293) and European (93%; 95% CI: 87–100%; *k* = 4; *n* = 2348) patients. Specificity was higher in Asian patients (41%; 95% CI: 25–58%; *k* = 5; *n* = 3680), followed by European (30%; 95% CI: 15–44%; *k* = 4; *n* = 2348) and African-American patients (15%; 95% CI: 5–34%; *k* = 1; *n* = 293) patients.

###### 3.4.2.1.4 Cutoff point 30–35

Pooled sensitivity and specificity were 87% (95% CI: 81–91%; I^2^ = 88.94%; *k* = 9; *n* = 5964) and 49% (95% CI: 41–58%; I^2^ = 95.53; *k* = 9; *n* = 5,964), respectively. Pooled AUC was 0.76 (95% CI: 0.72–0.80; *k* = 9; *n* = 5964).

Sensitivity was higher in Asian patients (91%; 95% CI: 84–97%; *k* = 4; *n* = 2794) and African Americans (91%; 95% CI: 78–100%; *k* = 1; *n* = 293) than in European patients (85%; 95% CI: 79–91%; *k* = 5; *n* = 2877). Specificity was higher in Asian patients (58%; 95% CI: 46–70%; *k* = 4; *n* = 2794), followed by European (49%; 95% CI: 41–57%; *k* = 5; *n* = 2877) and African-American (26%; 95% CI: 11–41%; *k* = 1; *n* = 293) patients.

###### 3.4.2.1.5 Cutoff point 35–40

Pooled sensitivity and specificity were 79% (95% CI: 66–88%; I^2^ = 83.13%; *k* = 5; *n* = 1164) and 56% (95% CI: 48–64%; I^2^ = 81.78; *k* = 5; *n* = 1164), respectively. The AUC obtained was 0.69 (95% CI: 0.64–0.72; *k* = 0; *n* = 5964). Despite the high level of heterogeneity between studies, no differences were observed between Asian and European patients (*p* = 0.14).

###### 3.4.2.1.6 Cutoff point 55

The sensitivity was 42% (95% CI: 32–53%; I^2^ = 73.61%; *k* = 3; *n* = 2028), while the specificity was 87% (95% CI: 72–95%; I^2^ = 98.28%; *k* = 3; *n* = 2028). Pooled AUC showed an accuracy of 0.60 (95% CI: 0.56–0.64; *k* = 3; *n* = 2028).

No subgroup analysis could be performed by ethnic origin.

##### 3.4.2.2 PHI density

Among the studies included for this test [20], [26], [38], [59], [90], the range for sensitivity was 90–97% and that for specificity was 32–39%.

##### 3.4.2.3 4Kscore test

Four studies provided data on this test [27], [34], [42], [44].

Assuming a risk of suffering csPCa of ≥7.5%, the sensitivity was 95.5% and the specificity was 32.1%, whereas when a risk of 12% was assumed, the sensitivity decreased to 90.1% and the specificity increased to 53.5%. The AUC ranged from 0.72 to 0.87.

##### 3.4.2.4 Stockholm3 test

Studies identified do not report sensitivity and specificity data [69], [70]. The AUC ranged from 0.77 to 0.86.

##### 3.4.2.5 Proclarix

The only included study obtained sensitivity and specificity of 91% and 22%, respectively [21].

### 3.5 Publication bias

No publication bias was identified, except in the PHI analysis with a cutoff point between 30 and 35 (*p* = 0.02). The results of the Egger tests and Deeks asymmetry graphs are available in Supplementary Table 7 and Supplementary Figure 1, respectively.

### 3.6 Discussion

The assessment of molecular biomarkers for the detection of csPCa is based on the data derived from 65 studies (*N* = 34 287), which evaluate their diagnostic accuracy in a population undergoing initial biopsy for suspected csPCa due to high PSA levels, family history, abnormal DRE, or altered multiparametric MRI. Quality of evidence for the tests included in the meta-analysis (PHI and SelectMDx) has been rated as low.

Included studies present high variability in terms of the assessed tests. Most (37) assessed the performance of the PHI [20], [22], [24], [25], [29], [30], [32], [38], [43], [46], [47], [48], [52], [54], [58], [59], [60], [61], [62], [64], [65], [67], [72], [73], [74], [75], [76], [77], [80], [83], [87], [89], [90], [91], [92], [93], [94], although only 16 of them could be included in the meta-analysis. Other blood tests assessed were PHI density [20], [26], [38], [59], [90], 4Kscore test [27], [34], [42], [44], Stockholm3 test [69], [70], and Proclarix test [21]. The diagnostic test based on the analysis of urine samples assessed by the highest number of studies was Progensa PCA3 [23], [28], [41], [50], [51], [52], [53], [55], [66], [81], [82], [95]. Other urine tests assessed were SelectMDx [36], [42], [49], [57], [78], MyProstateScore [33], [35], [37], [40], [81], TMPRSS2:ERG [37], [40], and ExoDx Prostate IntelliScore [31].

Approximately 77% of biopsies performed in men included in this SR did not yield a positive csPCa result. Furthermore, a 20% received a diagnosis of iPCa, placing them at risk of overdiagnosis, biopsy-related complications, and wasted health care resources, evidencing the need for better risk stratification.

Results of the assessed tests are measured on a continuous scale so that their behavior depends on where the cutoff point is set. However, information on established cutoff points was not provided in 25 of the included studies [22], [27], [32], [33], [34], [37], [38], [41], [46], [48], [55], [58], [59], [62], [69], [75], [76], [80], [81], [82], [87], [89], [91], [93], [94], and variability in terms of selected cutoffs is present among studies assessing the same test. Since the optimal 4Kscore, PCA3, and PHI cutoff points for the diagnosis of csPCa are not established, a comparison of diagnostic accuracy at different cutoff points was performed.

The results indicate that four analyzed tests (two urine tests and two blood tests) show an ability to identify ≥95% patients with csPCa: Progensa PCA3, with cutoff point 15; My-Prostate Score, using a cutoff point of >10; PHI, with any cutoff point between 15 and 30; and 4Kscore test, assuming a risk of csPCa of ≥7.5%. Using these tests and cutoff points, the ability to prevent unnecessary biopsies ranged between 14% and 37% [22], [24], [25], [28], [29], [30], [40], [42], [47], [51], [64], [65], [67], [72], [73], [77] which shows that theses could be useful as a noninvasive method of supporting the decision on whether or not the first prostate biopsy is necessary and, consequently, reducing the total number of unnecessary biopsies. However, it should be taken into account that only the results related to PHI are pooled effect estimates. The biomarker-based tests considered in this SR (particularly, PHI) would be included with triaging purposes in the diagnostic pathway for patients with a high clinical suspicion of csPCa but negative MRI results, in order to prevent unnecessary biopsies.

Of the nine SRs on biomarker-based tests for the management of PCa published to date to the best of our knowledge [62], [96], [97], [98], [99], [100], [101], [102], [103], only two focused on evaluating the use of these tests in discerning iPCa from csPCa and, consequently, in improving the decision-making process for first biopsies and treatment planning. Nevertheless, neither of them analyzes the available scientific evidence for all available tests, but rather for specific tests. Russo et al. [96] obtained for PHI and 4Kscore tests, sensitivity for the detection of csPCa of 93% and 87%, respectively, and specificity of 34% and 61%, respectively. Zappala et al. [101] evaluated the predictive precision of 4Kscore to discriminate between patients with and without csPCa, obtaining a pooled estimate for AUC of 0.80. Our results are consistent with these previous results.

One aspect to consider is the difference in test accuracy depending on the ethnic origin of patients. The evidence has shown possible improved performance of these tests in Asian populations, followed by Caucasian and African-American men. Confirmation of this finding would support the need for research on the best cutoff points based on patient ethnicity.

Available evidence exclusively consists of studies that evaluate the diagnostic validity of tests. However, using new tests with evidence of diagnostic utility does not directly imply improving decisions related to the diagnosis and treatment of PCa. Therefore, further research is needed to determine what effect the implementation of these tests would have on clinical decision making and patient-important health outcomes (eg, complications, recurrence-free survival, cancer survival, morbidity, and quality of life).

The main limitation of the present review is the methodological differences among studies, mainly the diversity or the lack of information about cutoff values. Moreover, a subgroup analysis to explore this issue could not always be performed. Another potential limitation is the possibility that some studies have not been included because those are not written in English or Spanish or because those are not indexed in the consulted databases. However, to the best of our knowledge, our SR is the most extensive review carried out to date on the effectiveness of the incorporation of tests, based on biomarkers in samples of blood or urine, for the identification of patients at high risk of csPCa. Methodologically, the SR benefits from rigorous methods following the fundamental principles of transparency and replicability; a comprehensive search, a peer selection, data extraction, and RoB assessment; and an assessment of the certainty of evidence on the basis of a structured and explicit approach.

## 4 Conclusions

Our findings indicate that PHI has high diagnostic accuracy for csPCa detection, and its incorporation in the diagnostic pathway could reduce unnecessary biopsies. However, there is a lack of evidence on the effects on patient consequences, supporting the need for well-conducted test-treatment RCTs in which investigators allocate patients to receive a PHI test or a control diagnostic approach (no test), and measure patient-important outcomes. Based on the pooled sensitivity estimate for SelectDMx, it is possible that the use of this test for the identification of patients with csPCa is not the best option. Finally, according to the limited available evidence, it is not possible to reach a clear conclusion on the other tests evaluated.

  ***Author contributions*:** Tasmania del Pino-Sedeño had full access to all the data in the study and takes responsibility for the integrity of the data and the accuracy of the data analysis.

*Study concept and design:* Tasmania del Pino-Sedeño.

*Acquisition of data*: Tasmania del Pino-Sedeño, Diego Infante-Ventura, Aythami de Armas Castellano.

*Analysis and interpretation of data*: Tasmania del Pino-Sedeño.

*Drafting of the manuscript*: Tasmania del Pino-Sedeño, Diego Infante-Ventura, Aythami de Armas Castellano, María M. Trujillo-Martín.

*Critical revision of the manuscript for important intellectual content*: Tasmania del Pino-Sedeño, Pedro de Pablos-Rodríguez, Antonio Rueda-Domínguez, Pedro Serrano-Aguilar, María M. Trujillo-Martín.

*Statistical analysis*: Tasmania del Pino-Sedeño.

*Obtaining funding*: Trujillo-Martín, Pedro Serrano-Aguilar.

*Administrative, technical, or material support*: Tasmania del Pino-Sedeño, Diego Infante-Ventura, Aythami de Armas Castellano.

*Supervision*: Tasmania del Pino-Sedeño, María M. Trujillo-Martín.

*Other*: None.

  ***Financial disclosures:*** Tasmania del Pino-Sedeño certifies that all conflicts of interest, including specific financial interests and relationships and affiliations relevant to the subject matter or materials discussed in the manuscript (eg, employment/affiliation, grants or funding, consultancies, honoraria, stock ownership or options, expert testimony, royalties, or patents filed, received, or pending), are the following: None.

  ***Funding/Support and role of the sponsor*:** The study was financed by the Ministry for Health of Spain in the framework of activities developed by the Spanish Network of Agencies for Health Technology Assessment and Services for the National Health System (RedETS).

  ***Acknowledgments:*** The authors would like to acknowledge Carlos Rodríguez for his help in the documentation tasks and Leticia Rodríguez for her support in the study search process. We are also grateful to Patrick Dennis for English language editing support with the final manuscript.

## Figures and Tables

**Supplementary figure:**