# Supplementary material for: Molecular Biomarkers for the Detection of Clinically Significant Prostate Cancer: A Systematic Review and Meta-analysis
Source: Eur Urol Open Sci. 2022 Nov 10;46:105–27. doi: 10.1016/j.euros.2022.10.017 (PMC9664479; doi:10.1016/j.euros.2022.10.017)
Supplement: Supplementary tables [file mmc1.docx]

Supplementary Table 1.

Search strategy

| **Medline** |
| --- |
| 1. exp Prostatic Neoplasms/ 2. (Prostat* adj5 (Neo?plasm$ or cancer or tumo?r or carcinom* or adenoma* or adenocarcin* or mass or masses or cyst* or oncolog* or sarcom* or malignan$*)).tw. 3. prostat*.hw. and exp Neoplasms/ 4. (suspected prostat* adj5 (Neo?plasm$ or cancer or tumo?r or carcinom* or adenoma* or adenocarcin* or mass or masses or cyst* or oncolog* or sarcom* or malignan$*)).tw. 5. 1 or 2 or 3 or 4 6. *Hematologic Tests/ 7. (SelectMDx or select MDx).tw. 8. h?ematologic test*.ti,ab. 9. (((PCA3 or PCA 3 or gene 3) adj5 (test* or score* or assay*)) or progensa).tw. 10. Prostarix*.tw. 11. (ExoDx Prostate or IntelliScore).tw. 12. (TMPRSS2 or TMPRSS2-ERG or Post-DRE Urine Test).tw. 13. (CELLSEARCH Circulating Tumor Cell* Test or CTC).tw. 14. ("PHI" or "prostat* health index").tw. 15. (Prostate Core Mitomic Test or PCMT).tw. 16. NADiA ProsVue.tw. 17. (Mi-Prostat* or MiPS or (mi adj5 score*)).tw. 18. (blood adj3 (test* or biomarker*)).ti,ab. 19. (urin* adj3 (test* or biomarker* or exosom*)).ti,ab. 20. (molecular adj3 (test* or biomarker*)).ti,ab. 21. urinar*.ti,ab. and Exosomes/ 22. 6 or 7 or 8 or 9 or 10 or 11 or 12 or 13 or 14 or 15 or 16 or 17 or 18 or 19 or 20 or 21 23. 5 and 22 24. limit 23 to yr="2010 -Current" 25. limit 24 to (english language or spanish) 26. limit 25 to humans |
| **EMBASE** |
| 1. 'prostate tumor'/exp 2. (prostat* NEAR/5 (neoplasm$ OR cancer OR tumor OR carcinom* OR adenoma* OR adenocarcin* OR mass OR masses OR cyst* OR oncolog* OR sarcom* OR malignan$*)):ti,ab 3. ('suspected prostat*' NEAR/5 (neo?plasm$ OR cancer OR tumo?r OR carcinom* OR adenoma* OR adenocarcin* OR mass OR masses OR cyst* OR oncolog* OR sarcom* OR malignan$*)):ti,ab,de 4. #1 OR #2 OR #3 5. 'blood examination' 6. 'hematologic test*':ti,ab 7. SelectMDx:ti,ab OR 'select mdx':ti,ab 8. ((pca3 OR 'pca 3' OR 'gene 3') NEAR/5 (test* OR score* OR assay*)):ti,ab 9. progensa:ti,ab,de 10. prostarix*:ti,ab,de 11. 'exodx prostate':ti,ab,de OR intelliscore:ti,ab,de 12. tmprss2:ti,ab,de OR 'tmprss2 erg':ti,ab,de OR 'post-dre urine test':ti,ab,de 13. 'cellsearch circulating tumor cell* test':ti,ab,de OR ctc:ti,ab,de 14. phi:ti,ab,de OR 'prostat* health index':ti,ab,de 15. 'prostate core mitomic test':ti,ab,de OR pcmt:ti,ab,de 16. 'nadia prosvue':ti,ab,de 17. 'mi-prostat*':ti,ab,de OR mips:ti,ab,de OR ((mi NEAR/5 score*):ti,ab,de) 18. (blood NEAR/2 (test* OR biomarker*)):ti,ab 19. (urin* NEAR/2 (test* OR biomarker* OR exosom*)):ti,ab 20. (molecular NEAR/2 (test* OR biomarker*)):ti,ab 21. 'urinary exosome'/exp 22. 'urinary exosome':ti,ab 23. #5 OR #6 OR #7 OR #8 OR #9 OR #10 OR #11 OR #12 OR #13 OR #14 OR #15 OR #16 OR #17 OR #18 OR #19 OR #20 OR #21 OR #22 24. #4 AND #23 25. 'clinically significant':ti,ab 26. 'high grade':ti,ab OR 'high-grade':ti,ab 27. 'cancer diagnosis' 28. 'cancer diagnosis':ti,ab OR 'cancer detection':ti,ab 29. #25 OR #26 OR #27 OR #28 30. #24 AND #29 31. #24 AND #29 AND [2010-2021]/py AND ([english]/lim OR [spanish]/lim) AND [humans]/lim |

Supplementary Table 2.

Excluded studies and reasons for exclusión

| **Design** |
| --- |
| 1. Gulati R, Morgan TM, A'mar T, Psutka SP, Tosoian JJ, Etzioni R. Overdiagnosis and Lives Saved by Reflex Testing Men With Intermediate Prostate-Specific Antigen Levels. J Natl Cancer Inst. 2020 Apr 1;112(4):384-390. doi: 10.1093/jnci/djz127. PMID: 31225597; PMCID: PMC7156927. |
| 1. Wu L, Shu X, Bao J, Guo X, Kote-Jarai Z, Haiman CA, Eeles RA, Zheng W; PRACTICAL, CRUK, BPC3, CAPS, PEGASUS Consortia. Analysis of Over 140,000 European Descendants Identifies Genetically Predicted Blood Protein Biomarkers Associated with Prostate Cancer Risk. Cancer Res. 2019 Sep 15;79(18):4592-4598. doi: 10.1158/0008-5472.CAN-18-3997. Epub 2019 Jul 23. PMID: 31337649; PMCID: PMC6744971. |
| 1. Bouttell J, Teoh J, Chiu PK, Chan KS, Ng CF, Heggie R, Hawkins N. Economic evaluation of the introduction of the Prostate Health Index as a rule-out test to avoid unnecessary biopsies in men with prostate specific antigen levels of 4-10 in Hong Kong. PLoS One. 2019 Apr 16;14(4):e0215279. doi: 10.1371/journal.pone.0215279. PMID: 30990840; PMCID: PMC6467402. |
| 1. Htoo KPP, Yamkamon V, Yainoy S, Suksrichavalit T, Viseshsindh W, Eiamphungporn W. Colorimetric detection of PCA3 in urine for prostate cancer diagnosis using thiol-labeled PCR primer and unmodified gold nanoparticles. Clin Chim Acta. 2019 Jan;488:40-49. doi: 10.1016/j.cca.2018.10.036. Epub 2018 Oct 30. PMID: 30389454. |
| 1. Govers TM, Hessels D, Vlaeminck-Guillem V, Schmitz-Dräger BJ, Stief CG, Martinez-Ballesteros C, Ferro M, Borque-Fernando A, Rubio-Briones J, Sedelaar JPM, van Criekinge W, Schalken JA. Cost-effectiveness of SelectMDx for prostate cancer in four European countries: a comparative modeling study. Prostate Cancer Prostatic Dis. 2019 Mar;22(1):101-109. doi: 10.1038/s41391-018-0076-3. Epub 2018 Aug 20. PMID: 30127462. |
| 1. McKiernan J, Donovan MJ, Margolis E, Partin A, Carter B, Brown G, Torkler P, Noerholm M, Skog J, Shore N, Andriole G, Thompson I, Carroll P. A Prospective Adaptive Utility Trial to Validate Performance of a Novel Urine Exosome Gene Expression Assay to Predict High-grade Prostate Cancer in Patients with Prostate-specific Antigen 2-10ng/ml at Initial Biopsy. Eur Urol. 2018 Dec;74(6):731-738. doi: 10.1016/j.eururo.2018.08.019. Epub 2018 Sep 17. PMID: 30237023. |
| 1. Govers TM, Caba L, Resnick MJ. Cost-Effectiveness of Urinary Biomarker Panel in Prostate Cancer Risk Assessment. J Urol. 2018 Dec;200(6):1221-1226. doi: 10.1016/j.juro.2018.07.034. Epub 2018 Aug 29. PMID: 30012363. |
| 1. Sathianathen NJ, Kuntz KM, Alarid-Escudero F, Lawrentschuk NL, Bolton DM, Murphy DG, Weight CJ, Konety BR. Incorporating Biomarkers into the Primary Prostate Biopsy Setting: A Cost-Effectiveness Analysis. J Urol. 2018 Dec;200(6):1215-1220. doi: 10.1016/j.juro.2018.06.016. Epub 2018 Jun 12. PMID: 29906434. |
| 1. Browne E, O'Malley K, Connolly S, O'Kelly F, Lundon D, Daly P, Galvin D, Hegarty N. The Hidden Burden of Outpatient Repeat PSA Testing in a Prospective Cohort. Ir Med J. 2017 May 10;110(5):564. PMID: 28737305. |
| 1. Dijkstra S, Govers TM, Hendriks RJ, Schalken JA, Van Criekinge W, Van Neste L, Grutters JPC, Sedelaar JPM, van Oort IM. Cost-effectiveness of a new urinary biomarker-based risk score compared to standard of care in prostate cancer diagnostics - a decision analytical model. BJU Int. 2017 Nov;120(5):659-665. doi: 10.1111/bju.13861. Epub 2017 Apr 29. PMID: 28370948. |
| 1. Heijnsdijk EA, Denham D, de Koning HJ. The Cost-Effectiveness of Prostate Cancer Detection with the Use of Prostate Health Index. Value Health. 2016 Mar-Apr;19(2):153-7. doi: 10.1016/j.jval.2015.12.002. Epub 2016 Jan 14. PMID: 27021748. |
| 1. Nicholson A, Mahon J, Boland A, Beale S, Dwan K, Fleeman N, Hockenhull J, Dundar Y. The clinical effectiveness and cost-effectiveness of the PROGENSA® prostate cancer antigen 3 assay and the Prostate Health Index in the diagnosis of prostate cancer: a systematic review and economic evaluation. Health Technol Assess. 2015 Oct;19(87):i-xxxi, 1-191. doi: 10.3310/hta19870. PMID: 26507078; PMCID: PMC4780983. |
| 1. Vlaeminck-Guillem V, Devonec M, Champetier D, Decaussi-Petrucci M, Paparel P, Perrin P, Ruffion A. Urinary PCA3 to predict prostate cancer in a cohort of 1015 patients. Prog Urol. 2015 Dec;25(16):1160-8, e1-8. English, French. doi: 10.1016/j.purol.2015.08.005. Epub 2015 Sep 12. PMID: 26376283. |
| 1. Nichol MB, Wu J, Huang J, Denham D, Frencher SK, Jacobsen SJ. Cost-effectiveness of Prostate Health Index for prostate cancer detection. BJU Int. 2012 Aug;110(3):353-62. doi: 10.1111/j.1464-410X.2011.10751.x. Epub 2011 Nov 11. PMID: 22077934. |
| 1. Kohaar I, Chen Y, Banerjee S, Borbiev T, Kuo HC, Ali A, Ravindranath L, Kagan J, Srivastava S, Dobi A, Sesterhenn IA, Rosner IL, Cullen J, Srivastava S, Petrovics G. A Urine Exosome Gene Expression Panel Distinguishes between Indolent and Aggressive Prostate Cancers at Biopsy. J Urol. 2021 Feb;205(2):420-425. doi: 10.1097/JU.0000000000001374. Epub 2020 Sep 18. PMID: 32945736. |
| 1. Teoh JY, Leung CH, Wang MH, Chiu PK, Yee CH, Ng CF, Wong MC. The cost-effectiveness of prostate health index for prostate cancer detection in Chinese men. Prostate Cancer Prostatic Dis. 2020 Dec;23(4):615-621. doi: 10.1038/s41391-020-0243-1. Epub 2020 Jun 30. PMID: 32606435. |
| 1. Huang D, Yang X, Wu Y, Lin X, Xu D, Na R, Xu J. Cost-Effectiveness Analysis of Prostate Health Index in Decision Making for Initial Prostate Biopsy. Front Oncol. 2020 Nov 24;10:565382. doi: 10.3389/fonc.2020.565382. PMID: 33330035; PMCID: PMC7732507. |
| 1. Chiu PK, Ng CF, Semjonow A, Vincendeau S, Houlgatte A, Lazzeri M, Guazzoni G, Stephan C, Haese A, Bruijne I, Teoh JY. A multi-centre evaluation of the role of Prostate Health Index (PHI) in regions with different prevalences of prostate cancer: A different reference range is needed for European and Asian. European Urology Supplements. 2018 Mar 1;17(2):e540-2. |
| 1. Vetterlein MW, Trinh QD, Chun FK. Novel noninvasive urine-based gene expression assay discriminates between low-and high-risk prostate cancer before biopsy. Transl Cancer Res. 2016 Aug 1;5(S2):S286-9. |
| 1. Tomlins SA. Urine PCA3 and TMPRSS2:ERG using cancer-specific markers to detect cancer. Eur Urol. 2014 Mar;65(3):543-5. doi: 10.1016/j.eururo.2012.12.001. Epub 2012 Dec 13. PMID: 23265385. |
| 1. Aubry W, Lieberthal R, Willis A, Bagley G, Willis SM 3rd, Layton A. Budget impact model: epigenetic assay can help avoid unnecessary repeated prostate biopsies and reduce healthcare spending. Am Health Drug Benefits. 2013 Jan;6(1):15-24. PMID: 24991343; PMCID: PMC4031702. |
| **Population** |
| 1. Johnson H, Guo J, Zhang X, Zhang H, Simoulis A, Wu AHB, Xia T, Li F, Tan W, Johnson A, Dizeyi N, Abrahamsson PA, Kenner L, Feng X, Zou C, Xiao K, Persson JL, Chen L. Development and validation of a 25-Gene Panel urine test for prostate cancer diagnosis and potential treatment follow-up. BMC Med. 2020 Dec 1;18(1):376. doi: 10.1186/s12916-020-01834-0. PMID: 33256740; PMCID: PMC7706045. |
| 1. Rubio-Briones J, Borque-Fernando A, Esteban LM, Mascarós JM, Ramírez-Backhaus M, Casanova J, Collado A, Mir C, Gómez-Ferrer A, Wong A, Aragón F, Calatrava A, López-Guerrero JA, Groskopf J, Schalken J, Van Criekinge W, Domínguez-Escrig J. Validation of a 2-gene mRNA urine test for the detection of ≥GG2 prostate cancer in an opportunistic screening population. Prostate. 2020 May;80(6):500-507. doi: 10.1002/pros.23964. Epub 2020 Feb 20. PMID: 32077525. |
| 1. Pepe P, Dibenedetto G, Pepe L, Pennisi M. Multiparametric MRI *Versus* SelectMDx Accuracy in the Diagnosis of Clinically Significant PCa in Men Enrolled in Active Surveillance. In Vivo. 2020 Jan-Feb;34(1):393-396. doi: 10.21873/invivo.11786. PMID: 31882504; PMCID: PMC6984119. |
| 1. Jeon J, Olkhov-Mitsel E, Xie H, Yao CQ, Zhao F, Jahangiri S, Cuizon C, Scarcello S, Jeyapala R, Watson JD, Fraser M, Ray J, Commisso K, Loblaw A, Fleshner NE, Bristow RG, Downes M, Vesprini D, Liu S, Bapat B, Boutros PC. Temporal Stability and Prognostic Biomarker Potential of the Prostate Cancer Urine miRNA Transcriptome. J Natl Cancer Inst. 2020 Mar 1;112(3):247-255. doi: 10.1093/jnci/djz112. PMID: 31161221; PMCID: PMC7073919. |
| 1. Frantzi M, Gomez Gomez E, Blanca Pedregosa A, Valero Rosa J, Latosinska A, Culig Z, Merseburger AS, Luque RM, Requena Tapia MJ, Mischak H, Carrasco Valiente J. CE-MS-based urinary biomarkers to distinguish non-significant from significant prostate cancer. Br J Cancer. 2019 Jun;120(12):1120-1128. doi: 10.1038/s41416-019-0472-z. Epub 2019 May 16. PMID: 31092909; PMCID: PMC6738044. |
| 1. Choi SY, Lim B, Kyung YS, Kim Y, Kim BM, Jeon BH, Park JC, Sohn YW, Lee JH, Uh JH, Jang S, Kim CS. Circulating Tumor Cell Counts in Patients With Localized Prostate Cancer Including Those Under Active Surveillance. In Vivo. 2019 Sep-Oct;33(5):1615-1620. doi: 10.21873/invivo.11645. PMID: 31471413; PMCID: PMC6755012. |
| 1. Ibrahim NH, Abdellateif MS, Thabet G, Kassem SH, El-Salam MA, El-Leithy AA, Selim MM. Combining PHI and miRNAs as Biomarkers in Prostate Cancer Diagnosis and Prognosis. Clin Lab. 2019 Jul 1;65(7). doi: 10.7754/Clin.Lab.2019.181213. PMID: 31307177. |
| 1. Fredsøe J, Rasmussen AKI, Laursen EB, Cai Y, Howard KA, Pedersen BG, Borre M, Mouritzen P, Ørntoft T, Sørensen KD. Independent Validation of a Diagnostic Noninvasive 3-MicroRNA Ratio Model (*uCaP*) for Prostate Cancer in Cell-Free Urine. Clin Chem. 2019 Apr;65(4):540-548. doi: 10.1373/clinchem.2018.296681. Epub 2019 Feb 6. PMID: 30728149. |
| 1. Ghorbanmehr N, Gharbi S, Korsching E, Tavallaei M, Einollahi B, Mowla SJ. miR-21-5p, miR-141-3p, and miR-205-5p levels in urine-promising biomarkers for the identification of prostate and bladder cancer. Prostate. 2019 Jan;79(1):88-95. doi: 10.1002/pros.23714. Epub 2018 Sep 7. PMID: 30194772. |
| 1. Möller A, Olsson H, Grönberg H, Eklund M, Aly M, Nordström T. The Stockholm3 blood-test predicts clinically-significant cancer on biopsy: independent validation in a multi-center community cohort. Prostate Cancer Prostatic Dis. 2019 Mar;22(1):137-142. doi: 10.1038/s41391-018-0082-5. Epub 2018 Aug 31. PMID: 30171228. |
| 1. Cheng YT, Chiang CH, Pu YS, Liu SP, Lu YC, Chang YK, Chang HC, Huang KH, Lee YJ, Chow PM, Hung SC, Huang CY. The application of p2PSA% and prostate health index in prostate cancer detection: A prospective cohort in a Tertiary Medical Center. J Formos Med Assoc. 2019 Jan;118(1 Pt 2):260-267. doi: 10.1016/j.jfma.2018.05.001. Epub 2018 May 18. PMID: 29779925. |
| 1. Zhao F, Olkhov-Mitsel E, Kamdar S, Jeyapala R, Garcia J, Hurst R, Hanna MY, Mills R, Tuzova AV, O'Reilly E, Kelly S, Cooper C; Movember Urine Biomarker Consortium, Brewer D, Perry AS, Clark J, Fleshner N, Bapat B. A urine-based DNA methylation assay, ProCUrE, to identify clinically significant prostate cancer. Clin Epigenetics. 2018 Nov 23;10(1):147. doi: 10.1186/s13148-018-0575-z. PMID: 30470249; PMCID: PMC6260648. |
| 1. Markou A, Lazaridou M, Paraskevopoulos P, Chen S, Świerczewska M, Budna J, Kuske A, Gorges TM, Joosse SA, Kroneis T, Zabel M, Sedlmayr P, Alix-Panabières C, Pantel K, Lianidou ES. Multiplex Gene Expression Profiling of In Vivo Isolated Circulating Tumor Cells in High-Risk Prostate Cancer Patients. Clin Chem. 2018 Feb;64(2):297-306. doi: 10.1373/clinchem.2017.275503. Epub 2017 Nov 9. PMID: 29122836. |
| 1. Hegde JV, Veruttipong D, Said JW, Reiter RE, Steinberg ML, King CR, Kishan AU. Prostate Cancer Antigen 3 Score Does Not Predict for Adverse Pathologic Features at Radical Prostatectomy or for Progression-free Survival in Clinically Localized, Intermediate- and High-risk Prostate Cancer. Urology. 2017 Sep;107:171-177. doi: 10.1016/j.urology.2017.05.028. Epub 2017 May 25. PMID: 28552819; PMCID: PMC6986738. |
| 1. Ferrer-Batallé M, Llop E, Ramírez M, Aleixandre RN, Saez M, Comet J, de Llorens R, Peracaula R. Comparative Study of Blood-Based Biomarkers, α2,3-Sialic Acid PSA and PHI, for High-Risk Prostate Cancer Detection. Int J Mol Sci. 2017 Apr 17;18(4):845. doi: 10.3390/ijms18040845. PMID: 28420168; PMCID: PMC5412429. |
| 1. Rubio-Briones J, Casanova J, Martínez F, Domínguez-Escrig JL, Fernández-Serra A, Dumont R, Ramírez-Backhaus M, Gómez-Ferrer A, Collado A, Rubio L, Molina A, Vanaclocha M, Sala D, Lopez-Guerrero JA. PCA3 as a second-line biomarker in a prospective controlled randomized opportunistic prostate cancer screening programme. Actas Urol Esp. 2017 Jun;41(5):300-308. English, Spanish. doi: 10.1016/j.acuro.2016.10.008. Epub 2017 Mar 23. PMID: 28342633. |
| 1. Gorges TM, Riethdorf S, von Ahsen O, Nastał Y P, Röck K, Boede M, Peine S, Kuske A, Schmid E, Kneip C, König F, Rudolph M, Pantel K. Heterogeneous PSMA expression on circulating tumor cells: a potential basis for stratification and monitoring of PSMA-directed therapies in prostate cancer. Oncotarget. 2016 Jun 7;7(23):34930-41. doi: 10.18632/oncotarget.9004. PMID: 27145459; PMCID: PMC5085200. |
| 1. Mengual L, Lozano JJ, Ingelmo-Torres M, Izquierdo L, Musquera M, Ribal MJ, Alcaraz A. Using gene expression from urine sediment to diagnose prostate cancer: development of a new multiplex mRNA urine test and validation of current biomarkers. BMC Cancer. 2016 Feb 9;16:76. doi: 10.1186/s12885-016-2127-2. PMID: 26856686; PMCID: PMC4746764. |
| 1. Schwen ZR, Tosoian JJ, Sokoll LJ, Mangold L, Humphreys E, Schaeffer EM, Partin AW, Ross AE. Prostate Health Index (PHI) Predicts High-stage Pathology in African American Men. Urology. 2016 Apr;90:136-40. doi: 10.1016/j.urology.2015.12.004. Epub 2015 Dec 10. PMID: 26688190. |
| 1. Zheng K, Dou Y, He L, Li H, Zhang Z, Chen Y, Ye A, Liu W, Kong L. Improved sensitivity and specificity for prostate cancer diagnosis based on the urine PCA3/PSA ratio acquired by sequence‑specific RNA capture. Oncol Rep. 2015 Nov;34(5):2439-44. doi: 10.3892/or.2015.4266. Epub 2015 Sep 9. PMID: 26351770. |
| 1. Jatkoe TA, Karnes RJ, Freedland SJ, Wang Y, Le A, Baden J. A urine-based methylation signature for risk stratification within low-risk prostate cancer. Br J Cancer. 2015 Mar 3;112(5):802-8. doi: 10.1038/bjc.2015.7. Epub 2015 Feb 19. PMID: 25695483; PMCID: PMC4453961. |
| 1. Ferro M, Lucarelli G, Bruzzese D, Perdonà S, Mazzarella C, Perruolo G, Marino A, Cosimato V, Giorgio E, Tagliamonte V, Bottero D, De Cobelli O, Terracciano D. Improving the prediction of pathologic outcomes in patients undergoing radical prostatectomy: the value of prostate cancer antigen 3 (PCA3), prostate health index (phi) and sarcosine. Anticancer Res. 2015 Feb;35(2):1017-23. PMID: 25667489. |
| 1. Stephan C, Wilkosz J, Różański W, Ecke TH, Lein M, Bryś M, Krześlak A, Chwatko G, Jung K. Urinary thiosulfate as failed prostate cancer biomarker - an exemplary multicenter re-evaluation study. Clin Chem Lab Med. 2015 Feb;53(3):477-83. doi: 10.1515/cclm-2014-0729. PMID: 25274944. |
| 1. Marszałł MP, Sroka W, Adamowski M, Słupski P, Jarzemski P, Siódmiak J, Odrowąż-Sypniewska G. Engrailed-2 protein as a potential urinary prostate cancer biomarker: a comparison study before and after digital rectal examination. Eur J Cancer Prev. 2015 Jan;24(1):51-6. doi: 10.1097/CEJ.0000000000000046. PMID: 25003607. |
| 1. Ma W, Diep K, Fritsche HA, Shore N, Albitar M. Diagnostic and prognostic scoring system for prostate cancer using urine and plasma biomarkers. Genet Test Mol Biomarkers. 2014 Mar;18(3):156-63. doi: 10.1089/gtmb.2013.0424. Epub 2014 Feb 10. PMID: 24512523. |
| 1. Cornu JN, Cancel-Tassin G, Egrot C, Gaffory C, Haab F, Cussenot O. Urine TMPRSS2:ERG fusion transcript integrated with PCA3 score, genotyping, and biological features are correlated to the results of prostatic biopsies in men at risk of prostate cancer. Prostate. 2013 Feb 15;73(3):242-9. doi: 10.1002/pros.22563. Epub 2012 Jul 20. PMID: 22821767. |
| 1. Lughezzani G, Lazzeri M, Larcher A, Lista G, Scattoni V, Cestari A, Buffi NM, Bini V, Guazzoni G. Development and internal validation of a Prostate Health Index based nomogram for predicting prostate cancer at extended biopsy. J Urol. 2012 Oct;188(4):1144-50. doi: 10.1016/j.juro.2012.06.025. Epub 2012 Aug 15. PMID: 22901589. |
| 1. van Poppel H, Haese A, Graefen M, de la Taille A, Irani J, de Reijke T, Remzi M, Marberger M. The relationship between Prostate CAncer gene 3 (PCA3) and prostate cancer significance. BJU Int. 2012 Feb;109(3):360-6. doi: 10.1111/j.1464-410X.2011.10377.x. Epub 2011 Aug 26. PMID: 21883822. |
| 1. Rubio-Briones J, Fernández-Serra A, Ramírez M, Rubio L, Collado A, Casanova J, Gómez-Ferrer A, Ricós JV, Monrós JL, Dumont R, Ortiz B, Iborra I, García-Casado Z, Solsona E, López-Guerrero JA. Resultados del uso expandido del PCA3 score en una población española con sospecha de cáncer de próstata [Outcomes of expanded use of PCA3 testing in a Spanish population with clinical suspicion of prostate cancer]. Actas Urol Esp. 2011 Nov-Dec;35(10):589-96. Spanish. doi: 10.1016/j.acuro.2011.04.001. Epub 2011 Jun 22. PMID: 21700365. |
| 1. Ochiai A, Okihara K, Kamoi K, Iwata T, Kawauchi A, Miki T, Fors Z. Prostate cancer gene 3 urine assay for prostate cancer in Japanese men undergoing prostate biopsy. Int J Urol. 2011 Mar;18(3):200-5. doi: 10.1111/j.1442-2042.2010.02711.x. PMID: 21332814. |
| 1. Roobol MJ, Schröder FH, van Leenders GL, Hessels D, van den Bergh RC, Wolters T, van Leeuwen PJ. Performance of prostate cancer antigen 3 (PCA3) and prostate-specific antigen in Prescreened men: reproducibility and detection characteristics for prostate cancer patients with high PCA3 scores (≥ 100). Eur Urol. 2010 Dec;58(6):893-9. doi: 10.1016/j.eururo.2010.09.030. Epub 2010 Sep 26. PMID: 20933321. |
| 1. Ploussard G, Haese A, Van Poppel H, Marberger M, Stenzl A, Mulders PF, Huland H, Bastien L, Abbou CC, Remzi M, Tinzl M, Feyerabend S, Stillebroer AB, Van Gils MP, Schalken JA, de La Taille A. The prostate cancer gene 3 (PCA3) urine test in men with previous negative biopsies: does free-to-total prostate-specific antigen ratio influence the performance of the PCA3 score in predicting positive biopsies? BJU Int. 2010 Oct;106(8):1143-7. doi: 10.1111/j.1464-410X.2010.09286.x. PMID: 20230386. |
| 1. Hessels D, van Gils MP, van Hooij O, Jannink SA, Witjes JA, Verhaegh GW, Schalken JA. Predictive value of PCA3 in urinary sediments in determining clinico-pathological characteristics of prostate cancer. Prostate. 2010 Jan 1;70(1):10-6. doi: 10.1002/pros.21032. PMID: 19708043. |
| 1. Stephan C, Jung K, Lein M, Rochow H, Friedersdorff F, Maxeiner A. PHI density prospectively improves prostate cancer detection. World J Urol. 2021 Jan 20. doi: 10.1007/s00345-020-03585-2. Epub ahead of print. PMID: 33471165. |
| 1. McKiernan J, Noerholm M, Tadigotla V, Kumar S, Torkler P, Sant G, Alter J, Donovan MJ, Skog J. A urine-based Exosomal gene expression test stratifies risk of high-grade prostate Cancer in men with prior negative prostate biopsy undergoing repeat biopsy. BMC Urol. 2020 Sep 1;20(1):138. doi: 10.1186/s12894-020-00712-4. PMID: 32873277; PMCID: PMC7466797. |
| 1. Stejskal J, Adamcová V, Záleský M, Novák V, Čapoun O, Fiala V, Dolejšová O, Sedláčková H, Veselý Š, Zachoval R. The predictive value of the prostate health index vs. multiparametric magnetic resonance imaging for prostate cancer diagnosis in prostate biopsy. World J Urol. 2021 Jun;39(6):1889-1895. doi: 10.1007/s00345-020-03397-4. Epub 2020 Aug 6. PMID: 32761380. |
| 1. Shore ND, Pieczonka CM, Henderson RJ, Bailen JL, Saltzstein DR, Concepcion RS, Beebe-Dimmer JL, Ruterbusch JJ, Levin RA, Wissmueller S, Le TH, Gillatt D, Chan DW, Campbell DH, Walsh BJ. Development and evaluation of the MiCheck test for aggressive prostate cancer. Urol Oncol. 2020 Aug;38(8):683.e11-683.e18. doi: 10.1016/j.urolonc.2020.03.010. Epub 2020 Apr 16. PMID: 32305266. |
| 1. Poulsen MH, Feddersen S, Albitar M, Poulsen CA, Lund M, Pedersen TB, Mortensen MA, Lund L. A prospective study of a urine and plasma biomarker test for the prediction of gleason ≥3 + 4 prostate cancer in a mixed cohort. Scand J Urol. 2020 Aug;54(4):323-327. doi: 10.1080/21681805.2020.1782979. Epub 2020 Jul 1. PMID: 32608296. |
| 1. Ried K, Tamanna T, Matthews S, Eng P, Sali A. New Screening Test Improves Detection of Prostate Cancer Using Circulating Tumor Cells and Prostate-Specific Markers. Front Oncol. 2020 Apr 23;10:582. doi: 10.3389/fonc.2020.00582. PMID: 32391268; PMCID: PMC7192049. |
| 1. Wu ZY, Yang C, Luo J, Deng SL, Wu B, Chen M. Establishment of reference intervals for serum [-2]proPSA (p2PSA), %p2PSA and prostate health index in healthy men. Onco Targets Ther. 2019 Aug 13;12:6453-6460. doi: 10.2147/OTT.S212340. PMID: 31616156; PMCID: PMC6698779. |
| 1. Gilani S, Shakery M, Shoureshi P, Salimi H, Maleki H, Alavi A, Khodadadi F. How is the association between urinary prostate cancer antigen 3 (PCA3) levels and Gleason scores in patients suspicious of prostate cancer? Int J Physiol Pathophysiol Pharmacol. 2019 Dec 15;11(6):283-288. PMID: 31993103; PMCID: PMC6971503. |
| 1. Alshalalfa M, Verhaegh GW, Gibb EA, Santiago-Jiménez M, Erho N, Jordan J, Yousefi K, Lam LLC, Kolisnik T, Chelissery J, Seiler R, Ross AE, Karnes RJ, Schaeffer EM, Lotan TT, Den RB, Freedland SJ, Davicioni E, Klein EA, Schalken JA. Low PCA3 expression is a marker of poor differentiation in localized prostate tumors: exploratory analysis from 12,076 patients. Oncotarget. 2017 Feb 7;8(31):50804-50813. doi: 10.18632/oncotarget.15133. PMID: 28881605; PMCID: PMC5584206. |
| 1. Stephan C, Vincendeau S, Houlgatte A, Miller K, Semjonow A. 2133 [-2] proPSA and Prostate Health Index (PHI) improve detection of prostate cancer at initial and repeated biopsies in young men (< 60 year old) preferentially detecting clinically significant cancer. The Journal of Urology. 2013 Apr;189(4S):e873-4. |
| **Intervention** |
| 1. Wang WW, Sorokin I, Aleksic I, Fisher H, Kaufman RP Jr, Winer A, McNeill B, Gupta R, Tilki D, Fleshner N, Klotz L, DiRienzo AG, Tenniswood M. Expression of Small Noncoding RNAs in Urinary Exosomes Classifies Prostate Cancer into Indolent and Aggressive Disease. J Urol. 2020 Sep;204(3):466-475. doi: 10.1097/JU.0000000000001020. Epub 2020 Mar 19. PMID: 32191585. |
| 1. Connell SP, O'Reilly E, Tuzova A, Webb M, Hurst R, Mills R, Zhao F, Bapat B, Cooper CS, Perry AS, Clark J, Brewer DS. Development of a multivariable risk model integrating urinary cell DNA methylation and cell-free RNA data for the detection of significant prostate cancer. Prostate. 2020 May;80(7):547-558. doi: 10.1002/pros.23968. Epub 2020 Mar 9. PMID: 32153047; PMCID: PMC7383590. |
| 1. Larsen LK, Jakobsen JS, Abdul-Al A, Guldberg P. Noninvasive Detection of High Grade Prostate Cancer by DNA Methylation Analysis of Urine Cells Captured by Microfiltration. J Urol. 2018 Oct;200(4):749-757. doi: 10.1016/j.juro.2018.04.067. Epub 2018 Apr 24. PMID: 29702096. |
| 1. Gnanapragasam VJ, Burling K, George A, Stearn S, Warren A, Barrett T, Koo B, Gallagher FA, Doble A, Kastner C, Parker RA. The Prostate Health Index adds predictive value to multi-parametric MRI in detecting significant prostate cancers in a repeat biopsy population. Sci Rep. 2016 Oct 17;6:35364. doi: 10.1038/srep35364. PMID: 27748407; PMCID: PMC5066204. |
| 1. Stephan C, Lein M, Matalon J, Kilic E, Zhao Z, Busch J, Jung K. Serum Vitamin D is Not Helpful for Predicting Prostate Cancer Aggressiveness Compared with the Prostate Health Index. J Urol. 2016 Sep;196(3):709-14. doi: 10.1016/j.juro.2016.03.009. Epub 2016 Mar 11. PMID: 26976204. |
| 1. Donovan MJ, Noerholm M, Bentink S, Belzer S, Skog J, O'Neill V, Cochran JS, Brown GA. A molecular signature of PCA3 and ERG exosomal RNA from non-DRE urine is predictive of initial prostate biopsy result. Prostate Cancer Prostatic Dis. 2015 Dec;18(4):370-5. doi: 10.1038/pcan.2015.40. Epub 2015 Sep 8. PMID: 26345389. |
| 1. Zhang W, Ren SC, Shi XL, Liu YW, Zhu YS, Jing TL, Wang FB, Chen R, Xu CL, Wang HQ, Wang HF, Wang Y, Liu B, Li YM, Fang ZY, Guo F, Lu X, Shen D, Gao X, Hou JG, Sun YH. A novel urinary long non-coding RNA transcript improves diagnostic accuracy in patients undergoing prostate biopsy. Prostate. 2015 May;75(6):653-61. doi: 10.1002/pros.22949. Epub 2015 Jan 18. PMID: 25597901. |
| 1. Guo J, Liu D, Zhang X, Johnson H, Feng X, Zhang H, Wu AHB, Chen L, Fang J, Xiao Z, Xiao K, Persson JL, Zou C. Establishing a Urine-Based Biomarker Assay for Prostate Cancer Risk Stratification. Front Cell Dev Biol. 2020 Dec 10;8:597961. doi: 10.3389/fcell.2020.597961. PMID: 33363151; PMCID: PMC7758396. |
| 1. Montoya Perez I, Jambor I, Pahikkala T, Airola A, Merisaari H, Saunavaara J, Alinezhad S, Väänänen RM, Tallgrén T, Verho J, Kiviniemi A, Ettala O, Knaapila J, Syvänen KT, Kallajoki M, Vainio P, Aronen HJ, Pettersson K, Boström PJ, Taimen P. Prostate Cancer Risk Stratification in Men With a Clinical Suspicion of Prostate Cancer Using a Unique Biparametric MRI and Expression of 11 Genes in Apparently Benign Tissue: Evaluation Using Machine-Learning Techniques. J Magn Reson Imaging. 2020 May;51(5):1540-1553. doi: 10.1002/jmri.26945. Epub 2019 Oct 6. PMID: 31588660. |
| 1. Bhakdi SC, Suriyaphol P, Thaicharoen P, Grote STK, Komoltri C, Chaiyaprasithi B, Charnkaew K. Accuracy of Tumour-Associated Circulating Endothelial Cells as a Screening Biomarker for Clinically Significant Prostate Cancer. Cancers (Basel). 2019 Jul 27;11(8):1064. doi: 10.3390/cancers11081064. PMID: 31357651; PMCID: PMC6721410. |
| 1. Ankerst DP, Goros M, Tomlins SA, Patil D, Feng Z, Wei JT, Sanda MG, Gelfond J, Thompson IM, Leach RJ, Liss MA. Incorporation of Urinary Prostate Cancer Antigen 3 and TMPRSS2:ERG into Prostate Cancer Prevention Trial Risk Calculator. Eur Urol Focus. 2019 Jan;5(1):54-61. doi: 10.1016/j.euf.2018.01.010. Epub 2018 Feb 13. PMID: 29422418; PMCID: PMC6077104. |
| 1. O'Reilly E, Tuzova AV, Walsh AL, Russell NM, O'Brien O, Kelly S, Dhomhnallain ON, DeBarra L, Dale CM, Brugman R, Clarke G, Schmidt O, O'Meachair S, Patil D, Pellegrini KL, Fleshner N, Garcia J, Zhao F, Finn S, Mills R, Hanna MY, Hurst R, McEvoy E, Gallagher WM, Manecksha RP, Cooper CS, Brewer DS, Bapat B, Sanda MG, Clark J, Perry AS. epiCaPture: A Urine DNA Methylation Test for Early Detection of Aggressive Prostate Cancer. JCO Precis Oncol. 2019;2019:PO.18.00134. doi: 10.1200/PO.18.00134. Epub 2019 Jan 14. PMID: 30801051; PMCID: PMC6383793. |
| 1. Gu CY, Huang YQ, Han CT, Zhu Y, Bo D, Meng J, Qin XJ, Ye DW. Clinical significance of urine prostatic exosomal protein in the diagnosis of prostate cancer. Am J Cancer Res. 2019 May 1;9(5):1074-1078. PMID: 31218113; PMCID: PMC6556599. |
| 1. Roberts MJ, Richards RS, Chow CW, Doi SA, Schirra HJ, Buck M, Samaratunga H, Perry-Keene J, Payton D, Yaxley J, Lavin MF, Gardiner RA. Prostate-based biofluids for the detection of prostate cancer: A comparative study of the diagnostic performance of cell-sourced RNA biomarkers. Prostate Int. 2016 Sep;4(3):97-102. doi: 10.1016/j.prnil.2016.04.002. Epub 2016 May 5. PMID: 27689066; PMCID: PMC5031901. |
| 1. Albitar M, Ma W, Lund L, Albitar F, Diep K, Fritsche HA, Shore N. Predicting Prostate Biopsy Results Using a Panel of Plasma and Urine Biomarkers Combined in a Scoring System. J Cancer. 2016 Feb 2;7(3):297-303. doi: 10.7150/jca.12771. PMID: 26918043; PMCID: PMC4747884. |
| **Protocol** |
| 1. Johnston E, Pye H, Bonet-Carne E, Panagiotaki E, Patel D, Galazi M, Heavey S, Carmona L, Freeman A, Trevisan G, Allen C, Kirkham A, Burling K, Stevens N, Hawkes D, Emberton M, Moore C, Ahmed HU, Atkinson D, Rodriguez-Justo M, Ng T, Alexander D, Whitaker H, Punwani S. INNOVATE: A prospective cohort study combining serum and urinary biomarkers with novel diffusion-weighted magnetic resonance imaging for the prediction and characterization of prostate cancer. BMC Cancer. 2016 Oct 21;16(1):816. doi: 10.1186/s12885-016-2856-2. PMID: 27769214; PMCID: PMC5073433. |
| **Abstract** |
| 1. Schwen ZR, Mamawala M, Tosoian JJ, Druskin SC, Ross AE, Sokoll LJ, Epstein JI, Carter HB, Gorin MA, Pavlovich CP. Prostate Health Index and multiparametric magnetic resonance imaging to predict prostate cancer grade reclassification in active surveillance. BJU Int. 2020 Sep;126(3):373-378. doi: 10.1111/bju.15101. Epub 2020 Jun 2. PMID: 32367635. |
| 1. Lopes Vendrami C, McCarthy RJ, Chatterjee A, Casalino D, Schaeffer EM, Catalona WJ, Miller FH. The Utility of Prostate Specific Antigen Density, Prostate Health Index, and Prostate Health Index Density in Predicting Positive Prostate Biopsy Outcome is Dependent on the Prostate Biopsy Methods. Urology. 2019 Jul;129:153-159. doi: 10.1016/j.urology.2019.03.018. Epub 2019 Mar 27. PMID: 30926382; PMCID: PMC6592745. |
| 1. Busetto GM, Del Giudice F, Maggi M, De Marco F, Porreca A, Sperduti I, Magliocca FM, Salciccia S, Chung BI, De Berardinis E, Sciarra A. Prospective assessment of two-gene urinary test with multiparametric magnetic resonance imaging of the prostate for men undergoing primary prostate biopsy. World J Urol. 2021 Jun;39(6):1869-1877. doi: 10.1007/s00345-020-03359-w. Epub 2020 Jul 17. PMID: 32681273. |
| 1. Chiu PK, Teoh JY, Cho CC, Lam YT, Hong YL, Wong SY, Chu WC, Sung JJ, Roobol MJ, Ng CF. Prostate cancer screening program with PSA, Prostate Health Index and MRI in a prospective cohort of Hong Kong Chinese men–an interim result. European Urology Open Science. 2020 Jul 1;19:e162. |
| 1. Vlaeminck V, Rebillard X, Lamy PJ, Potiron E, Colin P, Irani J, Roumiguié M, Vincendeau S, Cussenot O, Colombel M, De La Taille A. Clinical value of the integration of Prostate Health Index (PHI) in multiparametric MRI-based diagnostic strategies to detect clinically-significant prostate cancer. Results from the French prospective multicenter PHI-1 study. European Urology Open Science. 2020 Jul 1;19:e523-4. |
| 1. Guzman EF, Esteban MD, Hermosa PC, Figuero CR, Malo RV, Mediavilla EA, Formoso NG, Barseló ER, Blanco EH, Díez GV, Juanatey FC. Optimization in screening and diagnosis of Prostate Cancer (PCa): Use of Prostate Health Index (PHI) and mMRI in biopsy-naive patients. European Urology Open Science. 2020 Jul 1;19:e163-4. |
| 1. Noerholm M, Torkler P, Donovan MJ, Skog J, Mckiernan JM. Use of a urine exosome RNA test to assess risk of high-grade prostate cancer in men with a prior negative biopsy. European Urology Open Science. 2020 Jul 1;19:e169. |
| 1. Song WH, Baek SR, Nam JK, Park SW, Lee SD. Clinical implication of prostate health index density in patients with Prostate Image Reporting and Data System 3 lesion. European Urology Open Science. 2020 Jul 1;19:e513. |
| 1. Kohaar I, Banerjee S, Chen Y, Ali A, Kagan J, Srivastava S, Cullen J, Rosner I, Srivastava S, Petrovics G. Urine-based gene expression panels with focus on prostate cancer in African Americans. In cancer epidemiology biomarkers & prevention 2020 Jun 1 (Vol. 29, No. 6). 615 Chestnut st, 17th floor, Philadelphia, pa 19106-4404 USA: Amer Assoc Cancer Research. |
| 1. Nolte A, Wayne* G, Nagoda E, Wong V, Cedeno J, Perez A, Nieder A. MP27-08 Urinary exosome test and mp-mri for prostate cancer screening: balancing costs and benefits. The Journal of Urology. 2020 Apr;203(Supplement 4):e412-3. |
| 1. Richgels* J, Wackerbarth J, Helfand B, Glaser A. PD48-07 Clinical use of prostate health index (phi) for detection of prostate cancer in 3,318 patients. The Journal of Urology. 2020 Apr;203(Supplement 4):e996-7. |
| 1. Hendriks* R, van der Leest M, Israël B, Hannink G, Setiasti AY, Cornel E, Hulsbergen-van de Kaa C, Klaver S, Sedelaar M, Van Criekinge W, de Jong H. PD53-04 The SelectMDx urinary-biomarker test: role in the detection of high-grade prostate cancer and in combination with multi-parametric magnetic resonance imaging in a contemporary prospective cohort of biopsy-naïve men. The Journal of Urology. 2020 Apr;203(Supplement 4):e1097-8. |
| 1. Fernández* E, Domínguez M, Calleja P, Ramos E, Varea R, Alonso E, Herrero E, Velilla G, Campos F, Ballestero R, Azueta A. PD53-08 Prostate Health Index (PHI): Usefulness in biopsy-naïve patients. The Journal of Urology. 2020 Apr;203(Supplement 4):e1099-100. |
| 1. Pan* P, Fan YH, Lin TP, Chen WR, Huang TH, Wei TC, Huang IS, Lin CC, Huang EY, Chung HJ, Huang WJ. PD53-12 The Prostate Health Index improves predictive value in patients with pi-rads 3 lesions. The Journal of Urology. 2020 Apr;203(Supplement 4):e1101-2. |
| 1. Cani AK, Hu K, Liu CJ, Siddiqui J, Zheng Y, Han S, Nallandhighal S, Hovelson DH, Xiao L, Pham T, Eyrich NW, Zheng H, Vince R Jr, Tosoian JJ, Palapattu GS, Morgan TM, Wei JT, Udager AM, Chinnaiyan AM, Tomlins SA, Salami SS. Development of a Whole-urine, Multiplexed, Next-generation RNA-sequencing Assay for Early Detection of Aggressive Prostate Cancer. Eur Urol Oncol. 2021 Mar 31:S2588-9311(21)00046-8. doi: 10.1016/j.euo.2021.03.002. Epub ahead of print. PMID: 33812851. |
| 1. De La Calle C, Fasulo V, Cowan J, Herlemann A, Chu C, Gadzinski A, et al. Cooperberg MR, Shinohara K, Carroll P. Clinical utility of biomarkers 4K score, SelectMDx and ExoDx with MRI for the detection of high-grade prostate cancer. J Urol. 2020:203 |
| 1. Becher E, Wysock JS, Persily J, Loeb S, Lepor H. Concordance and Performance of 4Kscore and SelectMDx for Informing Decision to Perform Prostate Biopsy and Detection of Prostate Cancer. Urology. 2020 Jul;141:119-124. doi: 10.1016/j.urology.2020.02.032. Epub 2020 Apr 12. PMID: 32294481. |
| 1. Lantz A, Falagario UG, Jambor I, Martini A, Ratnani P, Wagaskar V, Treacy PJ, Veccia A, Bravi CA, Bashorun HO, Phillip D, Lewis S, Haines K, Cormio L, Carrieri G, Tewari A. Using biomarkers in patients with positive multiparametric magnetic resonance imaging: 4Kscore predicts the presence of cancer outside the index lesion. Int J Urol. 2021 Jan;28(1):47-52. doi: 10.1111/iju.14385. Epub 2020 Sep 27. PMID: 32985040. |
| 1. Oune N, Nguyentat MT, Sasani A, Bhatter P, Chahine C, Kohanteb PA, Glavis-Bloom J, Uchio E, Houshyar R. Radiogenomic model for detecting prostate cancer. Injournal of investigative medicine 2020 jan 1 (vol. 68, pp. A46-a46). British Med Assoc House, Tavistock Square, London Wc1h 9jr, England: BMJ Publishing Group. |
| 1. Garrido MM, Bernardino RM, Ribeiro RM, Pereira MH, Pinheiro LC, Guimarães JT. Prostate health index improves prostate cancer detection. En: 11th National Scientifc Congress SPML. Clinical Chemistry and Laboratory Medicine (CCLM*)*, vol. 58, no. 1, 2020, pp. eA1-eA77. doi:10.1515/cclm-2019-1173. |
| 1. Fasulo V, Lazzeri M, Corbetta M, Paciotti M, Maffei D, Soldà G, Domanico L, Frego N, Regis F, Diana P, Asselta R. TMPRSS2: ERG expression in prostate cancer—Imaging and clinicopathological correlations. |
| 1. McKiernan JM, Tutrone RF, Donovan MJ, Torkler P, Noerholm M, Alter J, Skog J. Urine exosome gene expression assay net benefit analysis in a large pooled cohort. |
| 1. Lee KM, Jones JA, Efimova O, DuVall SL, Lynch JA. The use of DNA promoter methylation testing for prostate cancer diagnosis in the Department of Veterans Affairs. |
| 1. Li L, Tao W, He Y, He T, Li Q, Wu Z, Deng W, Zhang L. A urine exosomal circRNA classifier for detection of high-grade prostate cancer at initial biopsy: A multicenter, retrospective study. |
| 1. Fairey AS, Paproski RJ, Pink D, Sosnowski DL, Vasquez C, Donnelly B, Hyndman ME, Aprikian AG, Beatty P, Lewis JD. Clinical analysis of the extracellular vesicle-fingerprint score blood test to refine the prediction of clinically significant prostate cancer and avoid prostate biopsy |
| 1. Busetto G, Del Giudice F, Maggi M, De Marco F, De Berardinis E, Sciarra A. Implementation of Select-MDx urinary test in the pre-biopsy setting for prostate cancer detection: Preliminary results from a prospective single-center pilot-study. European Urology Supplements. 2019 Oct 1;18(9):e3342. |
| 1. Woo J, Santasusagna S, Banks J, Dominguez-Andres A, Yadav K, Pippa R, Carceles-Cordon M, Lallas CD, Luyao G, Rodriguez-Bravo V, Tewari A. Urine extracellular vesicle GATA2 mRNA alone and in a multigene test predicts initial prostate biopsy result. Annals of Oncology. 2019 Oct 1;30:v349. |
| 1. Akdogan N, Aridogan IA, Izol V, Deger M, Gokalp F, Bayazit Y, Tansug MZ. Use of the prostate health index in the detection of prostate cancer at all PSA levels (use of prostate health index in prostate cancer). Int J Clin Pract. 2021 Apr;75(4):e13922. doi: 10.1111/ijcp.13922. Epub 2020 Dec 20. PMID: 33300224. |
| 1. Leow J, Yeow Y, Tan T. Utility of MRI fusion-targeted transrectal prostate biopsy and prostate health index in detection of clinically significant prostate cancer. In International Journal of Urology 2019 Aug 1 (Vol. 26, pp. 84-85). 111 River St, Hoboken 07030-5774, NJ USA: Wiley. |
| 1. Li TR, Hsieh PF. Prostate Health Index (PHI) for cancer detection: its correlation with gleason score, and pathological variables after radical prostatectomy in Taiwanese cohort. In International Journal of Urology 2019 Aug 1 (Vol. 26, pp. 180-180). 111 River St, Hoboken 07030-5774, NJ USA: Wiley. |
| 1. Filella X, Foj L, Augé JM, Molina R. Prostate Health Index density in prostate cancer detection. En: 45th Annual Congress of the International Society of Oncology and Biomarkers (ISOBM): Abstract book. Tumor biol.2019 Jul 3; 41(3):1337-1347. doi: 10.1177/1010428319842942. |
| 1. Cani AK, Hu K, Liu CJ, Siddiqui J, Zheng Y, Han S, Nallandhighal S, Hovelson DH, Xiao L, Pham T, Eyrich NW, Zheng H, Vince R Jr, Tosoian JJ, Palapattu GS, Morgan TM, Wei JT, Udager AM, Chinnaiyan AM, Tomlins SA, Salami SS. Development of a Whole-urine, Multiplexed, Next-generation RNA-sequencing Assay for Early Detection of Aggressive Prostate Cancer. Eur Urol Oncol. 2021 Mar 31:S2588-9311(21)00046-8. doi: 10.1016/j.euo.2021.03.002. Epub ahead of print. PMID: 33812851. |
| 1. Thandapani K, Chau TW, Hui WQ, Lincoln T, Chiong E. Is it necessary to biopsy indeterminate lesions on multiparametric MRI (mpMRI) scan of prostate?. InBJU INTERNATIONAL 2019 Apr 1 (Vol. 123, pp. 34-34). 111 River St, Hoboken 07030-5774, NJ USA: Wiley. |
| 1. Pan PH, Lin TP, Fan YH, Wei TC, Huang YS, Lin CC, et al. Prostate health index for prostate cancer detection in patient underwent magnetic resonance imaging transrectal ultrasound fusion prostate biopsy. J Urol. 2019 May 4; 201(4):e653–e654. doi:10.1097/01.JU.0000556342.55323.6f. |
| 1. Whelan* P, Tan WP, Poirier J, Deane L. MP28-17 The addition of biomarkers to PSA and mpMRI may improve prostate cancer detection rate. The Journal of Urology. 2019 Apr;201(Supplement 4):e410-1. |
| 1. Cheng YT, Chiu ST, Chiang CH, Chen CH, Pu YS, Hong JH, et al. Prostate health index density significantly improves the accuracy of predicting clinically significant prostate cancer in men undergoing prostate biopsy. J Urol. 2019 May 4; 201(4):e411. doi:10.1097/01.JU.0000557534.75965.1d. |
| 1. Haese A, Brawer M, Hessels D, Steyaert S, Trooskens G, Vlaeminck-Guillem V, et al. A 2-gene mRNA urine test for detection of high-grade prostate cancer prior to initial prostate biopsy. European Urology Supplements. 2019 Mar 1;18(1):e133-4. |
| 1. Gnanapragasam VJ, Barret T, Starling L, George A, Burling K, Saeb-Parsy K, Kastner C, Lamb B, Kim L. Use of the PHI assay as a first line triaging test in an image-guided prostate cancer diagnostic pathway. The PHI in Refining MRI (PRIM) study. European Urology Supplements. 2019 Mar 1;18(1):e129. |
| 1. Niknafs Y, Tosoian J, Morgan T, Tomlins S, Chinnaiyan AM. Identification of clinically significant prostate cancer using a novel multiplexed urine biomarker panel. European Urology Supplements. 2019 Mar 1;18(1):e135. |
| 1. Noerholm M, McKiernan J, Donovan MJ, Partin A, Carter B, Brown G, Margolis E, Torkler P, Skog J, Shore N, Andriole G. Pooled analysis of> 1000 patients enrolled in two independent prospective validation studies show consistent performance of a urine exosome gene expression assay to rule-out benign and low-grade prostate cancer at initial biopsy. European Urology Supplements. 2019 Mar 1;18(1):e132. |
| 1. Law MC, Lo KK, Wong KH, Kwok SC, Liu PL. Use of prostate health index (phi) before repeated transrectal ultrasound biopsy in Chinese population: a prospective study. In BJU International 2019 Mar 1 (Vol. 123, pp. 7-8). 111 River St, Hoboken 07030-5774, NJ USA: Wiley. |
| 1. Donovan MJ, Noerholm M, Torkler P, Skog J, Margolis E, Brown GA, McKiernan JM. Performance of a validated urine exosome gene expression test (EPI) in men within the USPSTF suggested age-group of 55 to 69 years at initial biopsy with a PSA 2-10 ng/ml. |
| 1. Woo J, Santasusagna S, Leiby B, Yadav KK, Dominguez-Andres A, Pippa R, Wang KR, Carceles-Cordon M, Fields S, Weil R, Stolovitzky GA. GATA2 exosomal mRNA: A novel urine biomarker for the diagnosis of clinically significant prostate cancer. |
| 1. Hoyer G, Crawford ED, Arangua P, Stanton W, La Rosa FG, Poage W, Lucia MS, van Bokhoven A, Werahera PN. SelectMDx versus Prostate Health Index in the identification of high-grade prostate cancer. |
| 1. Govers T, Resnick MJ, Trooskens G, Van Criekinge W, Schalken JA. Cost-effectiveness of a two-gene urine biomarker assay in MRI strategies for the initial detection of prostate cancer. |
| 1. Tilki D, Hessels D, Trooskens G, Mulders S, Brawer M, Van Criekinge W, Groskopf J, Haese A. Validation of a two-gene mRNA urine test for detection of high-grade prostate cancer in German men. |
| 1. Porzycki P, Ciszkowicz E. Initial experience with SelectMDx test in patients considered for prostate biopsy. European Urology Supplements. 2019 May 1;18(2):e2316. |
| 1. Barisiene M, Stanciute D, Jurkeviciene J, Zelvys A, Ulys A, Jankevicius F. Whether p2PSA,% p2PSA and Prostate Health Index improves clinically significant prostate cancer detection in men with PSA range 2-10 ng/ml. European Urology Supplements. 2019 May 1;18(3):e2437. |
| 1. Sanchis-Bonet A, Ortega-Polledo L, Garcia-Loarte EE, Rodriguez-Elena L, Bajo-Chueca A, Roman-Curto I, Sanchez-Chapado M. Utility of Prostate Health Index and Prostate Health Index Density in predicting detection of clinically significant prostate cancer in a cohort of patients with PSA in the grey zone and normal digital rectal examination. European Urology Supplements. 2019 Nov 1;18(11):e3420. |
| 1. Teoh JY, Leung CH, Wang MH, Chiu PK, Yee CH, Ng CF, Wong MC. The cost-effectiveness of prostate health index for prostate cancer detection in Chinese men. Prostate Cancer and Prostatic Diseases. 2020 Dec;23(4):615-21. |
| 1. Barisiene M, Stanciute D, Bakavicius A, Jurkeviciene J, Zelvys A, Ulys A, Jankevicius F. Diagnostic accuracy of [-2] proPSA,% p2PSA and Prostate Health Index for prostate cancer detection. |
| 1. Woon D, Cáceres JOH, Vangala S, Goldberg H, Chandrasekar T, Klaassen Z, et al. 4Kscore and PCA3 tests concordance in predicting positive biopsy for clinically significant prostate cancer. Can Urol Assoc J.2018 Jun; 12 (6Suppl2): S51-S136. |
| 1. Pchejetski D, Shah TT, Alshaker H, Ramadass A, Hunter E, Akoulitchev A, Winkler M. MP35-13 White blood cells from prostate cancer patients carry distinct chromosome conformations. The Journal of Urology. 2018 Apr;199(4S):e452-. |
| 1. McKiernan J, Donovan M, Margolis E, Partin A, Brown G, Torkler P, Noerholm M, Bentis C, Skog J, Shore N, Carroll P. MP46-05 Development of a clinical implementation plan (carepath) for a novel urine exosome gene expression assay as part of a two-cohort, adaptive decision impact utility trial. The Journal of Urology. 2018 Apr;199(4S):e607-. |
| 1. McKiernan J, Donovan MJ, Margolis E, Partin A, Carter B, Brown G, Torkler P, Noerholm M, Skog J, Shore N, Andriole G. A prospective adaptive utility trial to validate performance of a novel urine exosome gene expression assay to predict high-grade prostate cancer in patients with prostate-specific antigen 2–10 ng/ml at initial biopsy. European urology. 2018 Dec 1;74(6):731-8. |
| 1. Trooskens G, Yantisetiasti A, Hendriks R, Van Oort I, Mulders P, Hulsbergen-van de Kaa C, Van Criekinge W, Schalken J. MP46-11 Evaluation of two urinary rna biomarker tests with an epigenetic dna assay for the identification of men with high-grade prostate cancer. The Journal of Urology. 2018 Apr;199(4S):e610-. |
| 1. Sanda M, Huang E, Patil D, Sokoll L, Chan D, Scherr D, Siddiqui J, Wei J. MP40-13 Optimized clinical algorithm to combine testing of serum phi and urinary PCA3 to improve prostate cancer detection by reducing unnecesary biopsy. The Journal of Urology. 2018 Apr;199(4S):e524-. |
| 1. Hessels D, Govers TM, Vlaeminck-Guillem V, Schmitz-Dräger BJ, Stief CG, Martinez-Ballesteros C, Ferro M, Borque-Fernando A, Rubio-Briones J, Sedelaar JM, van Criekinge W. Cost-effectiveness of SelectMDx for prostate cancer in four European countries: a comparative modeling study. Prostate cancer and prostatic diseases. 2019 Mar;22(1):101-9. |
| 1. Lee M, Siddiqui J, Wei JT, Palapattu GS, Chinnaiyan AM, Tomlins SA, Salami SS. MP35-19 Association of urinary PCA3 and TMPRSS2:ERG risk groups with detection of prostate cancer at repeat biopsy in men with an initial negative prostate biopsy. The Journal of Urology. 2018 Apr;199(4S):e455-. |
| 1. Pang ST, Chang YH, Lin PH, Chang Y, Watson D, Segurado O, Lu SH, Lai JC, Shao HJ, Chang SE, Hsieh B. ProsPective clinical study of Prostate cancer (Pca) rule-out Blood test for Psa Gray Zone Patients usinG sensitive circulatinG tumor cell assay. Age. 2018 Feb 20;7(1):0-049. |
| 1. Dolejsova O, Fuchsova R, Topolcan O, Kucera R, Hora M, Svobodova H, et al. Can PHI better separate Gleason score 6 tumors and facilitate decision for right management for patients with prostate cancer? Tumor biol. 2017 Dec 22; 9-10. doi: 10.1177/1010428317742551. |
| 1. Dragomir A, Bonnevier E, Kassouf W, Aprikian A, Palenius E, Nazha S. Integrating Novel Screening Methods For Prostate Cancer, Cost-Utility Interventions. Value in Health. 2017 Oct 1;20(9):A589-90. |
| 1. Cheng YT, Chiang CH, Lu YC, Pu YS, Huang CY. The application of prostate health index in Tawanese prostate cancer detection-a prospective validation. In International Journal of Urology 2017 Aug 1 (Vol. 24, pp. 11-11). 111 River St, Hoboken 07030-5774, NJ USA: Wiley. |
| 1. Partin A, Torkler P, Noerholm M, Skog J, Donovan M. Interim results of a novel ‘adaptive’registration-utility trial assessing the performance of exoDX®(prostate) Intelliscore (epi); a non-dre urine exosome gene expression assay to predict high grade disease on initial biopsy.: PNFLBA-15. The Journal of Urology. 2017 Apr;197(4). |
| 1. Donovan M, Torkler P, Skog J, Noerholm M, Carroll P. Performance of a validated urine exosome gene expression assay to predict high-grade prostate cancer utilizing the International Society of Urological Pathology (ISUP) 2014 grading system. J. Clin. Oncol. 2017 Feb 20;35:49. |
| 1. Higgins CE, Neybold P, Barnes CR, Mathur V, Dong Y, Reeve M, et al. Validation of serum-based 4Kscore Test for detection of high grade prostate cancer. Clinical Chemistry. 2017; 63 (1): S5. |
| 1. Fiala V, Čapoun O, Sobotka R, Pešl M, Soukup V, Bernard O, Zámečník L, Dvořáček J, Hanuš T. The use of the prostate health index in daily clinical practice. European Urology Supplements. 2016 Dec 1;15(11):e1366. |
| 1. Sivkov A, Efremov G, Mihaylenko D, Grigoryeva M, Kaprin A, Apolikhin O. PCA3 and TMPRSS2-ERG for prostate cancer diagnosis. European Urology Supplements. 2016;13(15):e1561. |
| 1. Qian K. Integrating phi into prostate cancer detection. Malays J Pathol. 2016; 38 (2):187. |
| 1. PUN T, MAN C, CHU S, YEUNG V, YU T. The application of prostate health index in the patient selection for prostatic biopsies in a Hong Kong centre: C-OPRO-8631. International Journal of Urology. 2016 Jul;23. |
| 1. Lughezzani G;Lazzeri M;Hurle R;Buffi NM;Casale P;Fiorini G, et al. Clinical utility of PHI (Prostate Health Index) in men with tpsa> 10 ng/ml. results from a multicentric european study. |
| 1. Hendriks R, Dijkstra S, Cornel E, Jannink S, de Jong H, Hessels D, et al. Multicenter validation study of a molecular urine test to predict high-grade prostate cancer. J Urol. 2016 May 6; 195 (4S): e9-e10. doi:10.1016/j.juro.2016.02.1874. |
| 1. Glaser A, Cooper P, Roehl K, Catalona W. Comparison of prostate health index (PHI) and multiparametric magnetic resonance imaging (MRI) in detection of clinically-significant prostate cancer. J Urol. 2016 May 6; 195 (4S): e162-e163. doi: 10.1016/j.juro.2016.02.2570. |
| 1. Shi T, Gao Y, Qian WJ, Leach R, Thompson I, Quek SI, Ellis W, Vitello E, Liu A. S&T-10 Prostate cancer diagnosis by multiple secreted protein biomarkers in voided urine. The Journal of Urology. 2016 Apr;195(4S):e312-. |
| 1. Van Neste L, Trooskens G, Hendriks RJ, Schalken J, Van Criekinge W. MP53-04 Identification of high-grade prostate cancer using urine-based molecular biomarkers combined with clinical risk factors. The Journal of Urology. 2016 Apr;195(4S):e698. |
| 1. Donovan M, Bentink S, Noerholm M, O'Neill V, Skog J. 389 Performance of a validated urine-exosome gene signature on initial biopsy in the 4-10ng/ml PSA gray zone: Assessment of avoided biopsies and impact of an adjusted cut-point. European Urology Supplements. 2016;3(15):e389. |
| 1. Donovan M, Bentink S, Noerholm M, O'Neill V, Skog J. Extended analysis of a validated urine-exosome signature to predict high grade prostate cancer on initial biopsy: Performance across multiple sub-groups. |
| 1. Feibus AH, Sartor AO, Moparty K, Kattan MW, Chagin KM, Ledet EM, Levy J, Lee B, Thomas R, Silberstein JL. Utility of PCA3 and TMPRSS2: ERG urinary biomarkers in African American men undergoing prostate biopsy. |
| 1. Richard R, Gingras ME, Savoie M, Breault G, Fournier S, Daigle M, et al., A novel urinary EV-based prostate cancer assay that correctly predicts cancer grade as identified in the biopsy sample. J Extracell Vesicles. 2016; 5: 38. doi: 10.3402/jev.v5.31552. |
| 1. Gauhaar V, Tan GLL, Goh YSB. Prospective evaluation of the utility of% P2psa and the Prostate Health Index (PHI), in predicting prostate cancer in initial prostate biopsies of Singaporean men, with total PSA between 4.0 and 10 ng/ml. In BJU International 2015 Mar 1 (Vol. 115, pp. 3-3). 111 River St, Hoboken 07030-5774, NJ USA: Wiley. |
| 1. Nickens K, Ali A, Scoggin T, Tan SH, Ravindranath L, McLeod D, Tacha D, Sesterhenn I, Dobi A, Srivastava S, Petrovics G. Evaluation of a multiplex biomarker assay for the detection of prostate cancer cells in urine. |
| 1. De Luca S, Passera R, Bollito E, Vatrano S, Sottile A, Amparore D, Cattaneo G, Ottaviano G, Checcucci E, Bertolo R, Porpiglia F. Prostate Cancer Gene 3 Score, Prostate Health Index and percentage-free prostate-specific antigen: which is the best in differentiating histological inflammation from prostate cancer and other non-neoplastic alterations of the prostate at initial biopsy?. In Anticancer Research 2015 Jun 1 (Vol. 35, No. 6, pp. 3723-3724). Editorial Office 1^st^ Km Kapandritiou-Kalamou Rd Kapandriti, Po Box 22, Athens 19014, Greece: Int Inst Anticancer Research. |
| 1. Donovan MJ, Noerholm M, Bentink S, Belzer S, Skog J, Brown GA, Cochran JS, O’Neill V. Interim performance of a non-DRE urine exosome gene signature to predict Gleason≥ 7 prostate cancer on initial prostate needle biopsy from patients enrolled in a prospective observational trial. J. Clin. Oncol.. 2015 May 20;33(Suppl 15):5064. |
| 1. Rubio-Briones J, Casanova J, Dumont R, Rubio L, Fernandez-Serra A, Casanova-Salas I, Domínguez-Escrig J, Ramírez-Backhaus M, Collado A, Gómez-Ferrer A, Iborra I. 315 prospective randomized controlled study of the role of PSA and PCA3 testing in a sequential manner in an opportunistic screening program for early diagnosis of prostate cancer. European Urology Open Science. 2015 Apr 1;14(2):e315. |
| 1. Wei J, Siddiqui J, Siddiqui R, Chinnaiyan AM, Kunju LP, Mehra R, Snyder D, Tomlins SA. PD44-07 Effectiveness of serum PSA, urine PCA3 and urine TMPRSS2:ERG fusion as a novel urinary biomarker panel in clinical practice. The Journal of Urology. 2015 Apr;193(4S):e899-900. |
| 1. Sanda M, Feng Z, Wei J, Howard D, Rubin M, Groskopf J, Sokoll L, Chan D, Regan M, Patil D, Salami S. PI-08 Combining urine PCA3 and TMPRSS2:ERG tests to refine prostate cancer detection–validation study and health economic analysis. The Journal of Urology. 2015 Apr;193(4S):e982-3. |
| 1. Donovan MJ, Noerholm M, Bentink S, Belzer S, Skog J, Brown GA, Cochran JS, O'Neill V. A first catch, non-DRE urine exosome gene signature to predict Gleason 7 prostate cancer on an initial prostate needle biopsy. In Genitourinary Cancers Symposium. Orlando, FL 2015 Mar 1. |
| 1. SANT G. The 4Kscore test–a new blood biomarker for aggressive prostate cancer. Bju International. 2015 Feb;115:1-2. |
| 1. Skog J, Donovan MJ, Noerholm M, Bentink S, Belzer S, Brown GA, et al., A urine exosome gene signature predictive of aggressive prostate cancer (Gleason = 7). J Extracell Vesicles. 2015; 4(1): 190. |
| 1. Albitar M, Ma W, Albitar FS, Diep K, Fritsche HA, Shore ND. Using a combination of plasma and urine biomarkers along with serum PSA in predicting prostate cancer and screening for high-risk cancer. |
| 1. Fukabori Y, Yamazaki H, Yanagibayashi A, Yashi M, Abe H, Betsunoh H, Masuda A, Yuki H, Arai K, Kamai T. FGF7 as a candidate urine biomarker for prostate cancer detection. |
| 1. Nordström T, Vickers AJ, Lilja HG, Grönberg H, Eklund M. 339 A head to head comparison between a 4-kallikrein panel and a [-2] proPSA derivative model. European Urology Supplements. 2014 Apr 1;13(1):e339. |
| 1. Abrate A, Lazzeri M, Buffi N, Haese A, De La Taille A, McNicholas T, Palou Redorta J, Lughezzani G, Gadda GM, Lista G, Kinzikeeva E. Accuracy of p2PSA and derivatives (% p2PSA and PHI) in predicting prostate cancer in obese men from a multicenter European study. |
| 1. Leyten GH, Hessels D, Jannink SA, Smit FP, De Jong H, Melchers WJ, Cornel EB, De Reijke TM, Vergunst H, Kil P, Knipscheer BC. 351 QUATTRO, a four gene prognostic biomarker panel for prostate cancer. European Urology Supplements. 2014 Apr 1;13(1):e351. |
| 1. Ohigashi T, Yamashita H, Koshida T, Arakawa T, Mizuno R, Nakashima J. The efficacy of pca3 for predicting significant prostate cancer before biopsy. Urology. 2013; 82(3): S278-S279. |
| 1. Stephan C, Semjonow A, Schulze-Forster K, Cammann H, Hu X, Miller K, Jung K, Friedersdorff F. 859 Parallel measurement of urinary PCA3 and TMPRSS2: ERG with serum [− 2] proPSA based Phi for prostate cancer detection. European Urology Supplements. 2013;1(12):e859. |
| 1. De Luca S, Passera R, Milillo A, Coda R, Squeo MS, Randone DF. Chronic prostatitis and high-grade prostate intraepithelial neoplasia (HG-PIN) do not influence urinary PCA3 score. In Anticancer Research 2013 May 1 (Vol. 33, No. 5, pp. 2255-2256). Editorial Office 1^st^ Km Kapandritiou-Kalamou Rd Kapandriti, Po Box 22, Athens 19014, Greece: Int Inst Anticancer Research. |
| 1. Ferro M, Marino A, Gargiulo L, Cosimato V, Mazzarella C, Perruolo G, Bruzzese D, Terracciano D, Cimmino A, Scafuro C, Mastella F. Increasing prostate cancer detection by a model including prostate health index (PHI) and prostate cancer antigen 3 (PCA3). In Anticancer Research. 2013 May 1 (Vol. 33, No. 5, pp. 2275-2276). Editorial Office 1^st^ Km Kapandritiou-Kalamou Rd Kapandriti, Po Box 22, Athens 19014, Greece: Int Inst Anticancer Research. |
| 1. Semjonow A. Added value of new tests for prostate cancer detection. Biochim Clin. 2013; 37: S28-S29. |
| 1. Fukabori Y, Yamazaki H, Kambara T, Arai K, Kamai T. 2050 FGFR2IIIB, PSGR AND EZH2 as candidate urine biomarkers for prostate cancer detection. The Journal of Urology. 2013 Apr;189(4S):e841-. |
| 1. Rubio-Briones J, Casanova J, Dumont R, Rubio L, Fernandez-Serra A, Casanova-Salas I, Domínguez-Escrig J, Ramírez-Backhaus M, Collado A, Gómez-Ferrer A, Iborra I. Optimizing prostate cancer screening; prospective randomized controlled study of the role of PSA and PCA3 testing in a sequential manner in an opportunistic screening program. Actas Urológicas Españolas (English Edition). 2014 May 1;38(4):217-23. |
| 1. McNicholas T, Lazzeri M, Haese A, De La Taille A, Palou J, Lughezzani G, Scattoni V, Lista G, Larcher A, Cestari A, Buffi N. 137 PHI (Prostate Health Index) and% p2PSA for prediction of prostate cancer in men younger than 60 years of age. A nested-case control study from PROpsa Multicentric European Study (PROMEtheuS project). |
| 1. Apolikhin OI, Sivkov AV, Severin SE, Keshishev NG. Panel of Molecular Markers for Prostate Cancer Diagnosis. Annals of Oncology. 2014 Sep 1;25:iv78. |
| 1. Zhang W, Wang Z, Gupta M. Diagnostic and prognostic value of urinary PCA3 for prostate cancer: Comparison with, total and free PSA, and with PSA velocity. Clin Chem. 2012; 58(10): A101. |
| 1. Groskopf J, Ward J, Hertzman B, Bailen J, Franco N, Williams T, Koziol I, Henderson J, Bidair M, Gittelman M. PCA3 Molecular Urine Assay Pivotal US Clinical Study Confirms Utility for Predicting Repeat Biopsy Outcome. In Urology 2012 Sep 1 (Vol. 80, No. 3, pp. S30-S31). 360 Park Ave. South, New York, NY 10010-1710 USA: Elsevier Science INC. |
| 1. Fukabori Y, Yamazaki H, Kambara T, Yanagibayashi A, Arai K, Kamai T. FGFR2IIIb as a Candidate Urine Biomarker for Prostate Cancer Detection. In Urology. 2012 Sep 1 (Vol. 80, No. 3, pp. S281-S281). 360 Park Ave. South, New York, NY 10010-1710 USA: Elsevier Science INC. |
| 1. Chi-Fai N, KF CP, Ny L, Hc L, WM LK, Eddie S, Simon H. Role of prostate health index (phi) for prostate cancer detection in patients with serum PSA level between 4 to 10 ng/ml receiving first transrectal ultrasound guided biopsy: OP2504P-02. International Journal of Urology. 2012 Aug;19. |
| 1. Busetto GM, Salciccia S, Sciarra A, Giovannone R, Panebianco V, De Berardinis E, Gentile V, Di Silverio F. Multiparametric magnetic resonance imaging of the prostate and PCA3 urinary test: is time to re-evaluate prostate biopsy?. In Anticancer Research 2012 May 1 (Vol. 32, No. 5, pp. 1867-1867). Editorial Office 1^st^ Km Kapandritiou-Kalamou Rd Kapandriti, Po Box 22, Athens 19014, Greece: Int Inst Anticancer Research. |
| 1. Wei JT, Sanda MG, Thompson IM, Partin A, Feng Z, Sokoll L, Groskopf J, Brown E, Lotan Y, Kibel A, Lin D. The NCI Early Detection Research Network (EDRN) urinary PCA3 validation trial. |
| 1. Nepple K, Strope S, Kibel A, Sandhu G, Wiegand L, Kymes S. 135 Cost-analysis of PCA3 versus PSA in the detection of prostate cancer in men with a prior negative biopsy. The Journal of Urology. 2012 Apr;187(4S):e55-6. |
| 1. Chevli KK, Duff M, Yu C, Capuder B, Elshafei A, Malczewski S, Kattan M, Jones JS. 1208 Urinary PCA3 as a predictor for prostate cancer in a cohort of 1928 men undergoing initial prostate biopsy. The Journal of Urology. 2012 Apr;187(4S):e489. |
| 1. Tombal B, ANDRIOLE G, Groskopf J, Smets L, Stoevelaar H. PCA3 surpasses Best Clinical Judgment in selecting men requiring a repeat prostate biopsy: application of a RAND Decision Model to the REDUCE Trial Placebo Cohort: 03. Bju International. 2012 Apr;109. |
| 1. Tombal B, Andriole GL, Smets L, Stoevelaar H. Impact of PCA3 on the number of repeat prostate biopsies and sensitivity to detect high-grade cancer in the placebo cohort of the REDUCE study. Journal of Clinical Oncology. 2012 Feb 10;30(5_suppl):111-. |
| 1. Heijnsdijk EA, Huang JT, Denham D, De Koning HJ. 260 The cost-effectiveness of prostate cancer detection using Beckman Coulter Prostate Health Index. European Urology Supplements. 2012 Feb 1;11(1):e260. |
| 1. Di Silverio F, Salciccia S, Busetto GM, Panebianco V, Lisi D, Alfarone A, Parente U, Gentilucci A, Cattarino S, Innocenzi M, Giovannone R. Is prostate biopsy still necessary. Anticancer Res. 2011 May 1;31:1929-30. |
| 1. Tombal B, Andriole GL, Smets L, Stoevelaar H. 262 PCA3 can reduce repeat prostate biopsies with maintained sensitivity of detecting high-grade cancer: Application of an expert recommendations model to the placebo cohort of the REDUCE study. European Urology Supplements. 2012 Feb 1;11(1):e262. |
| 1. Lista G, Lughezzani G, Lazzeri M, Scattoni V, Nava L, Centemero A, Losa A, Fabbri F, Bini V, Rigatti P, Guazzoni G. Isoform [-2] proPSA (p2PSA) and its derivates,% p2PSA and PHI values differ between BPH, ASAP, HG-PIN and PCa in a set of contemporary men undergoing prostate biopsy. |
| 1. Šimunović D, Galić J. Repeated biopsy: limitations of prognostic factors in everyday clinical practice. European Urology Supplements. 2011 Oct 1. |
| 1. Kalogeropoulos T, Velaeti S, Dimitriadis E, Fliatouras C, Karaolides T, Klapsas V, Fasoulis L, Koutsiaris E, Arvanitakis T, Pappas A, Pandis N. S28 Study of multiple biomaRkeRS in uRine SampleS aS pRediCtive toolS foR pRoState CanCeR diagnoSiS-lateSt ReSultS. European Urology Supplements. 2011 Oct 1;10(9):583. |
| 1. Sokoll LJ, Marks L, Sanda M, Wei J, Klee G, Bangma C, et al. Value of the prostate health index (PHI)1 for prostate cancer detection in men undergoing first or repeat biopsy. A multi-center prospective clinical study. Clinical Chemistry. 2011; 57(10): A6 |
| 1. Segaran S, McMeekin F, Speakman M. The prostate cancer gene 3 (PCa3) urine test: Improving prediction of initial biopsy and management. Urology. 2011 Sep 1;78(3): S307-S308. doi: 10.1016/j.urology.2011.07.956 |
| 1. Szalay I, Bordás N, Pajor L, Farkas G, Salgó L. A new way in the early detection of prostate cancer: the [-2] pro prostate specific antigen. Clinical Chemistry and Laboratory Medicine. 2011;49(Suppl.):S276. |
| 1. Vincendeau S, Ramirez J, Durand X, Deligne E, Houlgatte A. 986 The Beckman Coulter Prostate Health Index (PHI) improves diagnostic accuracy in prostate cancer detection. European Urology Supplements. 2010 Apr 1;9(2):309. |
| 1. Lan W, Horn MJ, Truong W, LaSalle M, Ditrolio J. Enhancement of prostate cancer diagnosis through distribution analysis of a biomarker for apoptosis within subcellular fractions of prostate biopsy cores. |
| **Outcome** |
| 1. Kopecký J, Navláčilová V, Janoutová J, Janout V. Epidemiological study on more accurate diagnosis of prostate cancer. Cent Eur J Public Health. 2020 Mar;28(1):65-69. doi: 10.21101/cejph.a5720. PMID: 32228820. |
| 1. Ito K, Yokomizo A, Tokunaga S, Arai G, Sugimoto M, Akakura K, Hasumi H, Sakai H, Ouraji A, Oki R, Kashiwagi E, Kobori Y, Hirama H, Kitoh H, Uemura H, Hakariya T, Suzuki K; Members of PROPHET. Diagnostic Impacts of Clinical Laboratory Based p2PSA Indexes on any Grade, Gleason Grade Group 2 or Greater, or 3 or Greater Prostate Cancer and Prostate Specific Antigen below 10 ng/ml. J Urol. 2020 Jan;203(1):83-91. doi: 10.1097/JU.0000000000000495. Epub 2019 Aug 20. PMID: 31430244. |
| 1. Jagarlamudi KK, Zupan M, Kumer K, Fabjan T, Hlebič G, Eriksson S, Osredkar J, Smrkolj T. The combination of AroCell TK 210 ELISA with Prostate Health Index or prostate-specific antigen density can improve the ability to differentiate prostate cancer from noncancerous conditions. Prostate. 2019 Jun;79(8):856-863. doi: 10.1002/pros.23791. Epub 2019 Mar 19. PMID: 30889628. |
| 1. Rodon N, Trias I, Verdú M, Calvo M, Banus JM, Puig X. Correlation of mRNA-PCA3 urine levels with the new grading system in prostate cancer. Rev Esp Patol. 2019 Jan-Mar;52(1):20-26. doi: 10.1016/j.patol.2018.04.003. Epub 2018 Jun 18. PMID: 30583827. |
| 1. Wang J, Koo KM, Wang Y, Trau M. "Mix-to-Go" Silver Colloidal Strategy for Prostate Cancer Molecular Profiling and Risk Prediction. Anal Chem. 2018 Nov 6;90(21):12698-12705. doi: 10.1021/acs.analchem.8b02959. Epub 2018 Oct 12. PMID: 30260630. |
| 1. Hsieh PF, Chang CH, Yang CR, Huang CP, Chen WC, Yeh CC, Chou EC, Chen KL, Lien CS, Chen GH, Hsiao PJ, Chang YH, Wu HC. Prostate Health Index (PHI) improves prostate cancer detection at initial biopsy in Taiwanese men with PSA 4-10 ng/ml. Kaohsiung J Med Sci. 2018 Aug;34(8):461-466. doi: 10.1016/j.kjms.2018.02.007. Epub 2018 Apr 4. PMID: 30041764. |
| 1. Maccini MA, Westfall NJ, Van Bokhoven A, Lucia MS, Poage W, Maroni PD, Wilson SS, Glodé LM, Arangua P, Newmark J, Steiner M, Werahera PN, Crawford ED. The effect of digital rectal exam on the 4Kscore for aggressive prostate cancer. Prostate. 2018 May;78(7):506-511. doi: 10.1002/pros.23495. Epub 2018 Feb 19. PMID: 29460452; PMCID: PMC6237549. |
| 1. Sanchis-Bonet A, Barrionuevo-González M, Bajo-Chueca A, Morales-Palacios N, Sanchez-Chapado M. Does [-2]Pro-Prostate Specific Antigen Meet the Criteria to Justify Its Inclusion in the Clinical Decision-Making Process? Urol Int. 2018;100(2):146-154. doi: 10.1159/000481439. Epub 2018 Jan 18. PMID: 29346783. |
| 1. White J, Shenoy BV, Tutrone RF, Karsh LI, Saltzstein DR, Harmon WJ, Broyles DL, Roddy TE, Lofaro LR, Paoli CJ, Denham D, Reynolds MA. Clinical utility of the Prostate Health Index (phi) for biopsy decision management in a large group urology practice setting. Prostate Cancer Prostatic Dis. 2018 Apr;21(1):78-84. doi: 10.1038/s41391-017-0008-7. Epub 2017 Nov 20. PMID: 29158509; PMCID: PMC5895603. |
| 1. Tosoian JJ, Patel HD, Mamawala M, Landis P, Wolf S, Elliott DJ, Epstein JI, Carter HB, Ross AE, Sokoll LJ, Pavlovich CP. Longitudinal assessment of urinary PCA3 for predicting prostate cancer grade reclassification in favorable-risk men during active surveillance. Prostate Cancer Prostatic Dis. 2017 Sep;20(3):339-342. doi: 10.1038/pcan.2017.16. Epub 2017 Apr 18. PMID: 28417979; PMCID: PMC5555773. |
| 1. Vukovic I, Djordjevic D, Bojanic N, Babic U, Soldatovic I. Predictive value of [-2]propsa (p2psa) and its derivatives for the prostate cancer detection in the 2.0 to 10.0ng/ml PSA range. Int Braz J Urol. 2017 Jan-Feb;43(1):48-56. doi: 10.1590/S1677-5538.IBJU.2016.0256. PMID: 28124526; PMCID: PMC5293383. |
| 1. Okcelik S, Soydan H, Ates F, Berber U, Saygin H, Sonmez G, Karademir K. Evaluation of PCA3 and multiparametric MRI's: collective benefits before deciding initial prostate biopsy for patients with PSA level between 3-10ng/ml. Int Braz J Urol. 2016 May-Jun;42(3):449-55. doi: 10.1590/S1677-5538.IBJU.2015.0155. PMID: 27286106; PMCID: PMC4920560. |
| 1. Feibus AH, Sartor O, Moparty K, Chagin K, Kattan MW, Ledet E, Levy J, Lee B, Thomas R, Silberstein JL. Clinical Use of PCA3 and TMPRSS2:ERG Urinary Biomarkers in African-American Men Undergoing Prostate Biopsy. J Urol. 2016 Oct;196(4):1053-60. doi: 10.1016/j.juro.2016.04.075. Epub 2016 Apr 29. PMID: 27140073. |
| 1. Hendriks RJ, Dijkstra S, Jannink SA, Steffens MG, van Oort IM, Mulders PF, Schalken JA. Comparative analysis of prostate cancer specific biomarkers PCA3 and ERG in whole urine, urinary sediments and exosomes. Clin Chem Lab Med. 2016 Mar;54(3):483-92. doi: 10.1515/cclm-2015-0599. PMID: 26630694. |
| 1. Kaufmann S, Bedke J, Gatidis S, Hennenlotter J, Kramer U, Notohamiprodjo M, Nikolaou K, Stenzl A, Kruck S. Prostate cancer gene 3 (PCA3) is of additional predictive value in patients with PI-RADS grade III (intermediate) lesions in the MR-guided re-biopsy setting for prostate cancer. World J Urol. 2016 Apr;34(4):509-15. doi: 10.1007/s00345-015-1655-8. Epub 2015 Aug 13. PMID: 26267808. |
| 1. Zhu Y, Han CT, Zhang GM, Liu F, Ding Q, Xu JF, Vidal AC, Freedland SJ, Ng CF, Ye DW. Development and external validation of a prostate health index-based nomogram for predicting prostate cancer. Sci Rep. 2015 Oct 16;5:15341. doi: 10.1038/srep15341. PMID: 26471350; PMCID: PMC4607975. |
| 1. Yang S, Pinney SM, Mallick P, Ho SM, Bracken B, Wu T. Impact of Oxidative Stress Biomarkers and Carboxymethyllysine (an Advanced Glycation End Product) on Prostate Cancer: A Prospective Study. Clin Genitourin Cancer. 2015 Oct;13(5):e347-51. doi: 10.1016/j.clgc.2015.04.004. Epub 2015 Apr 18. PMID: 25972296; PMCID: PMC4564335. |
| 1. Nickens KP, Ali A, Scoggin T, Tan SH, Ravindranath L, McLeod DG, Dobi A, Tacha D, Sesterhenn IA, Srivastava S, Petrovics G. Prostate cancer marker panel with single cell sensitivity in urine. Prostate. 2015 Jun 15;75(9):969-75. doi: 10.1002/pros.22981. Epub 2015 Mar 23. PMID: 25808739; PMCID: PMC4424114. |
| 1. Nygård Y, Haukaas SA, Eide GE, Halvorsen OJ, Gravdal K, Frugård J, Akslen LA, Beisland C. Prostate cancer antigen-3 (PCA3) and PCA3-based nomograms in the diagnosis of prostate cancer: an external validation of Hansen's nomogram on a Norwegian cohort. Scand J Urol. 2015 Feb;49(1):8-15. doi: 10.3109/21681805.2014.949841. Epub 2014 Aug 20. PMID: 25141128. |
| 1. Delongchamps NB, Younes P, Denjean L, Zerbib M, Bories PN. TMPRSS2-ERG fusion transcripts expression in patients referred for prostate biopsy: combining detection in urine and needle rinse material. World J Urol. 2015 Jun;33(6):807-11. doi: 10.1007/s00345-014-1359-5. Epub 2014 Jul 5. PMID: 24997128. |
| 1. Fossati N, Lazzeri M, Haese A, McNicholas T, de la Taille A, Buffi NM, Lughezzani G, Gadda GM, Lista G, Larcher A, Abrate A, Mistretta F, Bini V, Redorta JP, Graefen M, Guazzoni G. Clinical performance of serum isoform [-2]proPSA (p2PSA), and its derivatives %p2PSA and the Prostate Health Index, in men aged <60 years: results from a multicentric European study. BJU Int. 2015 Jun;115(6):913-20. doi: 10.1111/bju.12718. Epub 2015 Mar 26. PMID: 24589357. |
| 1. Wang F, Ren S, Chen R, Lu J, Shi X, Zhu Y, Zhang W, Jing T, Zhang C, Shen J, Xu C, Wang H, Wang H, Wang Y, Liu B, Li Y, Fang Z, Guo F, Qiao M, Wu C, Wei Q, Xu D, Shen D, Lu X, Gao X, Hou J, Sun Y. Development and prospective multicenter evaluation of the long noncoding RNA MALAT-1 as a diagnostic urinary biomarker for prostate cancer. Oncotarget. 2014 Nov 30;5(22):11091-102. doi: 10.18632/oncotarget.2691. PMID: 25526029; PMCID: PMC4294360. |
| 1. De Luca S, Passera R, Bollito E, Manfredi M, Scarpa RM, Sottile A, Randone DF, Porpiglia F. Comparison of prostate cancer gene 3 score, prostate health index and percentage free prostate-specific antigen for differentiating histological inflammation from prostate cancer and other non-neoplastic alterations of the prostate at initial biopsy. Anticancer Res. 2014 Dec;34(12):7159-65. PMID: 25503144. |
| 1. Filella X, Foj L, Alcover J, Augé JM, Molina R, Jiménez W. The influence of prostate volume in prostate health index performance in patients with total PSA lower than 10 μg/L. Clin Chim Acta. 2014 Sep 25;436:303-7. doi: 10.1016/j.cca.2014.06.019. Epub 2014 Jun 27. PMID: 24978824. |
| 1. Foj L, Milà M, Mengual L, Luque P, Alcaraz A, Jiménez W, Filella X. Real-time PCR PCA3 assay is a useful test measured in urine to improve prostate cancer detection. Clin Chim Acta. 2014 Aug 5;435:53-8. doi: 10.1016/j.cca.2014.04.025. Epub 2014 May 5. PMID: 24803095. |
| 1. Friedersdorff F, Manus P, Miller K, Lein M, Jung K, Stephan C. Serum testosterone improves the accuracy of Prostate Health Index for the detection of prostate cancer. Clin Biochem. 2014 Jul;47(10-11):916-20. doi: 10.1016/j.clinbiochem.2014.02.002. Epub 2014 Feb 12. PMID: 24530341. |
| 1. Lughezzani G, Lazzeri M, Haese A, McNicholas T, de la Taille A, Buffi NM, Fossati N, Lista G, Larcher A, Abrate A, Mistretta A, Bini V, Palou Redorta J, Graefen M, Guazzoni G. Multicenter European external validation of a prostate health index-based nomogram for predicting prostate cancer at extended biopsy. Eur Urol. 2014 Nov;66(5):906-12. doi: 10.1016/j.eururo.2013.12.005. Epub 2013 Dec 16. PMID: 24361258. |
| 1. Rubio-Briones J, Casanova J, Dumont R, Rubio L, Fernandez-Serra A, Casanova-Salas I, Domínguez-Escrig J, Ramírez-Backhaus M, Collado A, Gómez-Ferrer A, Iborra I, Monrós JL, Ricós JV, Solsona E, Salas D, Martínez F, Lopez-Guerrero JA. Optimizing prostate cancer screening; prospective randomized controlled study of the role of PSA and PCA3 testing in a sequential manner in an opportunistic screening program. Actas Urol Esp. 2014 May;38(4):217-23. English, Spanish. doi: 10.1016/j.acuro.2013.09.007. Epub 2013 Oct 27. PMID: 24169211. |
| 1. Ng CF, Chiu PK, Lam NY, Lam HC, Lee KW, Hou SS. The Prostate Health Index in predicting initial prostate biopsy outcomes in Asian men with prostate-specific antigen levels of 4-10 ng/ml. Int Urol Nephrol. 2014 Apr;46(4):711-7. doi: 10.1007/s11255-013-0582-0. Epub 2013 Oct 18. PMID: 24136184. |
| 1. Chan SW, Nguyen PN, Violette P, Brimo F, Taguchi Y, Aprikian A, Chen JZ. Early detection of clinically significant prostate cancer at diagnosis: a prospective study using a novel panel of TMPRSS2:ETS fusion gene markers. Cancer Med. 2013 Feb;2(1):63-75. doi: 10.1002/cam4.49. Epub 2013 Feb 3. PMID: 24133629; PMCID: PMC3797559. |
| 1. Ferro M, Bruzzese D, Perdonà S, Marino A, Mazzarella C, Perruolo G, D'Esposito V, Cosimato V, Buonerba C, Di Lorenzo G, Musi G, De Cobelli O, Chun FK, Terracciano D. Prostate Health Index (Phi) and Prostate Cancer Antigen 3 (PCA3) significantly improve prostate cancer detection at initial biopsy in a total PSA range of 2-10 ng/ml. PLoS One. 2013 Jul 4;8(7):e67687. doi: 10.1371/journal.pone.0067687. PMID: 23861782; PMCID: PMC3701535. |
| 1. Lazzeri M, Haese A, Abrate A, de la Taille A, Redorta JP, McNicholas T, Lughezzani G, Lista G, Larcher A, Bini V, Cestari A, Buffi N, Graefen M, Bosset O, Le Corvoisier P, Breda A, de la Torre P, Fowler L, Roux J, Guazzoni G. Clinical performance of serum prostate-specific antigen isoform [-2]proPSA (p2PSA) and its derivatives, %p2PSA and the prostate health index (PHI), in men with a family history of prostate cancer: results from a multicentre European study, the PROMEtheuS project. BJU Int. 2013 Aug;112(3):313-21. doi: 10.1111/bju.12217. PMID: 23826841. |
| 1. Ochiai A, Okihara K, Kamoi K, Oikawa T, Shimazui T, Murayama S, Tomita K, Umekawa T, Uemura H, Miki T. Clinical utility of the prostate cancer gene 3 (PCA3) urine assay in Japanese men undergoing prostate biopsy. BJU Int. 2013 May;111(6):928-33. doi: 10.1111/j.1464-410X.2012.11683.x. Epub 2013 Jan 18. PMID: 23331404. |
| 1. Dimitriadis E, Kalogeropoulos T, Velaeti S, Sotiriou S, Vassiliou E, Fasoulis L, Klapsas V, Synesiou M, Apostolaki A, Trangas T, Pandis N. Study of genetic and epigenetic alterations in urine samples as diagnostic markers for prostate cancer. Anticancer Res. 2013 Jan;33(1):191-7. PMID: 23267145. |
| 1. Stephan C, Jung K, Semjonow A, Schulze-Forster K, Cammann H, Hu X, Meyer HA, Bögemann M, Miller K, Friedersdorff F. Comparative assessment of urinary prostate cancer antigen 3 and TMPRSS2:ERG gene fusion with the serum [-2]proprostate-specific antigen-based prostate health index for detection of prostate cancer. Clin Chem. 2013 Jan;59(1):280-8. doi: 10.1373/clinchem.2012.195560. Epub 2012 Dec 4. PMID: 23213079. |
| 1. Loeb S, Sokoll LJ, Broyles DL, Bangma CH, van Schaik RH, Klee GG, Wei JT, Sanda MG, Partin AW, Slawin KM, Marks LS, Mizrahi IA, Shin SS, Cruz AB, Chan DW, Roberts WL, Catalona WJ. Prospective multicenter evaluation of the Beckman Coulter Prostate Health Index using WHO calibration. J Urol. 2013 May;189(5):1702-6. doi: 10.1016/j.juro.2012.11.149. Epub 2012 Nov 30. PMID: 23206426; PMCID: PMC4273580. |
| 1. Ito K, Miyakubo M, Sekine Y, Koike H, Matsui H, Shibata Y, Suzuki K. Diagnostic significance of [-2]pro-PSA and prostate dimension-adjusted PSA-related indices in men with total PSA in the 2.0-10.0 ng/ml range. World J Urol. 2013 Apr;31(2):305-11. doi: 10.1007/s00345-012-0927-9. Epub 2012 Aug 18. PMID: 22903772. |
| 1. Perdonà S, Bruzzese D, Ferro M, Autorino R, Marino A, Mazzarella C, Perruolo G, Longo M, Spinelli R, Di Lorenzo G, Oliva A, De Sio M, Damiano R, Altieri V, Terracciano D. Prostate health index (phi) and prostate cancer antigen 3 (PCA3) significantly improve diagnostic accuracy in patients undergoing prostate biopsy. Prostate. 2013 Feb 15;73(3):227-35. doi: 10.1002/pros.22561. Epub 2012 Jul 20. PMID: 22821756. |
| 1. Ramos CG, Valdevenito R, Vergara I, Anabalon P, Sanchez C, Fulla J. PCA3 sensitivity and specificity for prostate cancer detection in patients with abnormal PSA and/or suspicious digital rectal examination. First Latin American experience. Urol Oncol. 2013 Nov;31(8):1522-6. doi: 10.1016/j.urolonc.2012.05.002. Epub 2012 Jun 9. PMID: 22687565. |
| 1. Katafigiotis I, Tyritzis SI, Stravodimos KG, Alamanis C, Pavlakis K, Vlahou A, Makridakis M, Katafigioti A, Garbis SD, Constantinides CA. Zinc α2-glycoprotein as a potential novel urine biomarker for the early diagnosis of prostate cancer. BJU Int. 2012 Dec;110(11 Pt B):E688-93. doi: 10.1111/j.1464-410X.2012.11501.x. Epub 2012 Sep 28. PMID: 23020913. |
| 1. Crawford ED, Rove KO, Trabulsi EJ, Qian J, Drewnowska KP, Kaminetsky JC, Huisman TK, Bilowus ML, Freedman SJ, Glover WL Jr, Bostwick DG. Diagnostic performance of PCA3 to detect prostate cancer in men with increased prostate specific antigen: a prospective study of 1,962 cases. J Urol. 2012 Nov;188(5):1726-31. doi: 10.1016/j.juro.2012.07.023. Epub 2012 Sep 19. PMID: 22998901. |
| 1. Ferro M, Bruzzese D, Perdonà S, Mazzarella C, Marino A, Sorrentino A, Di Carlo A, Autorino R, Di Lorenzo G, Buonerba C, Altieri V, Mariano A, Macchia V, Terracciano D. Predicting prostate biopsy outcome: prostate health index (phi) and prostate cancer antigen 3 (PCA3) are useful biomarkers. Clin Chim Acta. 2012 Aug 16;413(15-16):1274-8. doi: 10.1016/j.cca.2012.04.017. Epub 2012 Apr 20. PMID: 22542564. |
| 1. Klatte T, Waldert M, de Martino M, Schatzl G, Mannhalter C, Remzi M. Age-specific PCA3 score reference values for diagnosis of prostate cancer. World J Urol. 2012 Jun;30(3):405-10. doi: 10.1007/s00345-011-0749-1. Epub 2011 Aug 30. PMID: 21877172. |
| 1. de la Taille A, Irani J, Graefen M, Chun F, de Reijke T, Kil P, Gontero P, Mottaz A, Haese A. Clinical evaluation of the PCA3 assay in guiding initial biopsy decisions. J Urol. 2011 Jun;185(6):2119-25. doi: 10.1016/j.juro.2011.01.075. Epub 2011 Apr 15. PMID: 21496856. |
| 1. Perdonà S, Cavadas V, Di Lorenzo G, Damiano R, Chiappetta G, Del Prete P, Franco R, Azzarito G, Scala S, Arra C, De Sio M, Autorino R. Prostate cancer detection in the "grey area" of prostate-specific antigen below 10 ng/ml: head-to-head comparison of the updated PCPT calculator and Chun's nomogram, two risk estimators incorporating prostate cancer antigen 3. Eur Urol. 2011 Jan;59(1):81-7. doi: 10.1016/j.eururo.2010.09.036. Epub 2010 Oct 14. PMID: 20947244. |
| 1. Henderson J, Ghani KR, Cook J, Fahey M, Schalken J, Thilagarajah R. The role of PCA3 testing in patients with a raised prostate-specific antigen level after Greenlight photoselective vaporization of the prostate. J Endourol. 2010 Nov;24(11):1821-4. doi: 10.1089/end.2010.0196. Epub 2010 Oct 21. PMID: 20964483. |
| 1. Rigau M, Morote J, Mir MC, Ballesteros C, Ortega I, Sanchez A, Colás E, Garcia M, Ruiz A, Abal M, Planas J, Reventós J, Doll A. PSGR and PCA3 as biomarkers for the detection of prostate cancer in urine. Prostate. 2010 Dec 1;70(16):1760-7. doi: 10.1002/pros.21211. PMID: 20672322. |
| 1. Galasso F, Giannella R, Bruni P, Giulivo R, Barbini VR, Disanto V, Leonardi R, Pansadoro V, Sepe G. PCA3: a new tool to diagnose prostate cancer (PCa) and a guidance in biopsy decisions. Preliminary report of the UrOP study. Arch Ital Urol Androl. 2010 Mar;82(1):5-9. PMID: 20593708. |
| 1. Le BV, Griffin CR, Loeb S, Carvalhal GF, Kan D, Baumann NA, Catalona WJ. [-2]Proenzyme prostate specific antigen is more accurate than total and free prostate specific antigen in differentiating prostate cancer from benign disease in a prospective prostate cancer screening study. J Urol. 2010 Apr;183(4):1355-9. doi: 10.1016/j.juro.2009.12.056. Epub 2010 Feb 19. PMID: 20171670; PMCID: PMC3537165. |
| 1. Rice KR, Chen Y, Ali A, Whitman EJ, Blase A, Ibrahim M, Elsamanoudi S, Brassell S, Furusato B, Stingle N, Sesterhenn IA, Petrovics G, Miick S, Rittenhouse H, Groskopf J, McLeod DG, Srivastava S. Evaluation of the ETS-related gene mRNA in urine for the detection of prostate cancer. Clin Cancer Res. 2010 Mar 1;16(5):1572-6. doi: 10.1158/1078-0432.CCR-09-2191. Epub 2010 Feb 16. PMID: 20160063. |
| 1. Yaseen SJ, Balindy SS, Mbchb, Facs, Fibms. Evaluation of prostatic health index (PHI) and -2proPSA in the diagnosis of prostatic cáncer. Pak J Med Health Sci. 2020 Oct-Dec; 14(4): 1410-1413. |
| 1. Stanton WN, Crawford ED, Arangua PB, La Rosa FG, van Bokhoven A, Lucia MS, Poage WL, Partin A, Maroni P, Werahera PN. Assessment of high-grade prostate cancer risk using prostate cancer biomarkers. Can J Urol. 2020 Feb;27(1):10080-10085. PMID: 32065862. |
| 1. Akdogan N, Aridogan IA, Izol V, Deger M, Gokalp F, Bayazit Y, Tansug MZ. Use of the prostate health index in the detection of prostate cancer at all PSA levels (use of prostate health index in prostate cancer). Int J Clin Pract. 2021 Apr;75(4):e13922. doi: 10.1111/ijcp.13922. Epub 2020 Dec 20. PMID: 33300224. |
| 1. Huang Y, Gu X, Wang Y, Hou J, Qi X. Prostate health index is useful for prostate cancer detecting in Chinese people. Translational Cancer Research. 2019 Jun 1;8(3):836-9. |
| 1. Sriplakich S, Lojanapiwat B, Chongruksut W, Phuriyaphan S, Kitirattakarn P, Jun-Ou J, Amantakul A. Prospective performance of the Prostate Health Index in prostate cancer detection in the first prostate biopsy of men with a total prostatic specific antigen of 4-10 ng/ml and negative digital rectal examination. Prostate Int. 2018 Dec;6(4):136-139. doi: 10.1016/j.prnil.2018.02.002. Epub 2018 Feb 15. PMID: 30505815; PMCID: PMC6251939. |
| 1. Nordström T, Adolfsson J, Grönberg H, Eklund M. Effects of increasing the PSA cutoff to perform additional biomarker tests before prostate biopsy. BMC Urol. 2017 Oct 3;17(1):92. doi: 10.1186/s12894-017-0281-8. PMID: 28974201; PMCID: PMC5627473. |
| 1. Puche-Sanz I, Alvarez-Cubero MJ, Pascual-Geler M, Rodríguez-Martínez A, Delgado-Rodríguez M, García-Puche JL, Expósito J, Robles-Fernández I, Entrala-Bernal C, Lorente JA, Cózar-Olmo JM, Serrano MJ. A comprehensive study of circulating tumour cells at the moment of prostate cancer diagnosis: biological and clinical implications of EGFR, AR and SNPs. Oncotarget. 2017 Jul 31;8(41):70472-70480. doi: 10.18632/oncotarget.19718. PMID: 29050295; PMCID: PMC5642570. |
| 1. Osredkar J, Kumer K, Fabjan T, Hlebič G, Podnar B, Lenart G, Smrkolj T. The performance of [-2] proPSA and prostate health index tumor markers in prostate cancer diagnosis. Journal of Laboratory Medicine. 2016 Dec 1;40(6):419-24. |
| 1. Tomlins SA, Aubin SM, Siddiqui J, Lonigro RJ, Sefton-Miller L, Miick S, Williamsen S, Hodge P, Meinke J, Blase A, Penabella Y, Day JR, Varambally R, Han B, Wood D, Wang L, Sanda MG, Rubin MA, Rhodes DR, Hollenbeck B, Sakamoto K, Silberstein JL, Fradet Y, Amberson JB, Meyers S, Palanisamy N, Rittenhouse H, Wei JT, Groskopf J, Chinnaiyan AM. Urine TMPRSS2:ERG fusion transcript stratifies prostate cancer risk in men with elevated serum PSA. Sci Transl Med. 2011 Aug 3;3(94):94ra72. doi: 10.1126/scitranslmed.3001970. PMID: 21813756; PMCID: PMC3245713. |
| 1. Essawi ML, El-Azim SA, Morsy AA, Hassan HA. Glutathione S-transferase Pi-1 gene methylation in early detection of prostate cancer in Egyptian patients. Minerva Biotecnologica. 2010 Jun 1;22(2):47. |

Supplementary Table 3.

Quality Assessment of the included studies. QUADAS-2 tool

| **Study** | **Patient Selection** | | | | | **Index Test** | | | | **Reference Standard** | | | | **Flow and Timing** | | | | |
| --- | --- | --- | --- | --- | --- | --- | --- | --- | --- | --- | --- | --- | --- | --- | --- | --- | --- | --- |
|  | A-2 | A-3 | A-4 | RoB | AP | A-2 | A-3 | RoB | AP | A-2 | A-3 | RoB | AP | A-3 | A-4 | A-5 | A-6 | RoB |
| Abrate, 2015 [1] | ? | N | Y | ☹ | ☺ | ? | N | ☹ | ☺ | Y | Y | ☺ | ☺ | Y | Y | Y | Y | ☺ |
| Babajide, 2021 [2] | ? | N | Y | ☹ | ? | ? | N | ☹ | ☺ | Y | ? | ? | ☺ | Y | N | Y | Y | ☹ |
| Barisiene, 2020 [3] | ? | Y | Y | ? | ☺ | ? | Y | ? | ☺ | Y | Y | ☺ | ☺ | Y | Y | Y | Y | ☺ |
| Bertok, 2020 [4] | ? | Y | Y | ? | ☺ | ? | N | ☹ | ☺ | Y | Y | ☺ | ☺ | Y | Y | Y | Y | ☺ |
| Boegemann, 2016 [5] | ? | Y | N | ☹ | ☺ | ? | N | ☹ | ☺ | Y | ? | ? | ☺ | Y | Y | Y | N | ☹ |
| BuYetto, 2020 [6] | Y | Y | Y | ☺ | ☺ | Y | ? | ? | ☺ | Y | ? | ? | ☺ | ? | Y | Y | Y | ? |
| Cao, 2018 [7] | N | Y | Y | ☹ | ☺ | ? | N | ☹ | ? | Y | ? | ? | ☺ | ? | Y | Y | N | ☹ |
| Catalona, 2011 [8] | Y | N | ? | ☹ | ☺ | Y | N | ☹ | ☺ | Y | Y | ☺ | ☺ | Y | Y | Y | N | ☹ |
| Chiu, 2016 [9] | Y | Y | Y | ☺ | ? | ? | N | ☹ | ☺ | Y | ? | ? | ☺ | Y | Y | Y | Y | ☺ |
| Chiu, 2016b [10] | Y | Y | Y | ☺ | ? | ? | Y | ? | ☺ | Y | Y | ☺ | ☺ | Y | Y | Y | Y | ☺ |
| Chiu, 2019^1^ [11] | ? | Y | Y | ? | ☺ | ? | N | ☹ | ☺ | Y | ? | ? | ☺ | Y | Y | Y | Y | ☺ |
| Chiu, 2019^2^ [11] | ? | Y | Y | ? | ? | Y | N | ☹ | ☺ | Y | Y | ☺ | ☺ | Y | Y | Y | N | ☹ |
| Choi, 2020 [12] | ? | Y | Y | ? | ☺ | ? | Y | ? | ☺ | Y | Y | ☺ | ☺ | Y | Y | Y | Y | ☺ |
| de la Calle, 2015^3^ [13] | ? | Y | Y | ? | ☺ | Y | N | ☹ | ☺ | Y | Y | ☺ | ☺ | Y | Y | Y | Y | ☺ |
| de la Calle, 2015^4^ [13] | ? | Y | Y | ? | ☺ | Y | N | ☹ | ☺ | Y | Y | ☺ | ☺ | Y | Y | Y | Y | ☺ |
| Druskin, 2018 [14] | ? | Y | Y | ? | ☺ | ? | Y | ? | ☺ | Y | Y | ☺ | ☺ | Y | Y | Y | Y | ☺ |
| Falagario, 2021 [15] | ? | Y | Y | ? | ☺ | ? | N | ☹ | ☺ | Y | ? | ? | ☺ | Y | Y | Y | Y | ☺ |
| Fan, 2019 [16] | Y | Y | Y | ☺ | ? | ? | N | ☹ | ☺ | Y | ? | ? | ☺ | Y | Y | Y | Y | ☺ |
| Filella, 2014^5^ [17] | ? | Y | Y | ? | ☺ | ? | N | ☹ | ☺ | Y | ? | ? | ☺ | Y | Y | Y | Y | ☺ |
| Filella, 2014^6^ [17] | ? | Y | Y | ? | ☺ | ? | N | ☹ | ☺ | Y | ? | ? | ☺ | Y | Y | Y | Y | ☺ |
| Filella, 2014^7^ [17] | ? | Y | Y | ? | ☺ | ? | N | ☹ | ☺ | Y | ? | ? | ☺ | Y | Y | Y | Y | ☺ |
| Foj, 2020 [18] | ? | Y | Y | ? | ☺ | ? | N | ☹ | ☺ | Y | ? | ? | ☺ | Y | Y | Y | Y | ☺ |
| Foley, 2016 [19] | ? | Y | Y | ? | ☺ | Y | N | ☹ | ☺ | Y | ? | ? | ☺ | Y | Y | Y | ? | ? |
| Furuya, 2017 [20] | Y | Y | Y | ☺ | ? | ? | N | ☹ | ☺ | Y | Y | ☺ | ☺ | Y | Y | Y | Y | ☺ |
| Guazzoni. 2011 [21] | Y | Y | Y | ☺ | ☺ | Y | N | ☹ | ☺ | Y | Y | ☺ | ☺ | Y | Y | Y | Y | ☺ |
| Haese, 2019 [22] | Y | Y | Y | ☺ | ☺ | Y | N | ☹ | ☺ | Y | ? | ? | ☺ | Y | Y | Y | Y | ☺ |
| Hansen, 2013 [23] | ? | Y | Y | ? | ☺ | ? | Y | ? | ☺ | Y | ? | ? | ☺ | ? | Y | Y | N | ☹ |
| Hsieh, 2020 [24] | ? | Y | N | ☹ | ? | Y | Y | ☺ | ☺ | Y | Y | ☺ | ☺ | Y | Y | Y | N | ☹ |
| Kim, 2020 [25] | Y | Y | N | ☹ | ☺ | ? | ? | ☹ | ☺ | Y | ? | ? | ☺ | ? | Y | Y | N | ☹ |
| Kotova, 2020 [26] | N | Y | N | ☹ | ? | Y | N | ☹ | ☺ | Y | ? | ? | ☺ | ? | Y | Y | N | ☹ |
| Lazzeri, 2013 [27] | Y | Y | Y | ☺ | ☺ | ? | N | ☹ | ☺ | Y | Y | ☺ | ☺ | Y | Y | Y | Y | ☺ |
| Lazzeri, 2016 [28] | N | N | Y | ☹ | ☺ | ? | N | ☹ | ☺ | Y | ? | ? | ☺ | Y | Y | Y | Y | ☺ |
| Leyten, 2015 [29] | N | ? | Y | ☹ | ☺ | Y | N | ☹ | ☺ | Y | Y | ☺ | ☺ | ? | Y | Y | Y | ? |
| Loeb, 2015 [30] | ? | Y | Y | ? | ☺ | Y | N | ☹ | ☺ | Y | Y | ☺ | ☺ | Y | Y | Y | Y | ☺ |
| Loeb, 2017 [31] | ? | Y | N | ☹ | ☺ | ? | N | ☹ | ☺ | Y | Y | ☺ | ☺ | Y | Y | Y | N | ☹ |
| McKiernan, 2016 [32] | ? | Y | Y | ? | ? | Y | Y | ☺ | ☺ | Y | Y | ☺ | ☺ | ? | Y | Y | ? | ☹ |
| Mearini, 2014 [33] | Y | Y | Y | ☺ | ☺ | ? | N | ☹ | ☺ | Y | Y | ☺ | ☺ | Y | Y | Y | Y | ☺ |
| Morote, 2016 [34] | Y | Y | ? | ? | ☺ | ? | N | ☹ | ☺ | Y | ? | ? | ☺ | Y | Y | Y | Y | ☺ |
| Mortezavi, 2020 [35] | ? | Y | N | ☹ | ☺ | Y | N | ☹ | ☺ | Y | Y | ☺ | ☺ | Y | ? | ? | Y | ☹ |
| Na, 2014 [36] | Y | Y | ? | ? | ☺ | ? | N | ☹ | ☺ | Y | ? | ? | ☺ | Y | Y | Y | ? | ? |
| Na, 2017 [37] | Y | Y | ? | ? | ? | ? | N | ☹ | ☺ | Y | ? | ? | ☺ | Y | Y | Y | ? | ? |
| Nordström, 2015 [38] | N | N | Y | ☹ | ☺ | Y | N | ☹ | ☺ | Y | Y | ☺ | ☺ | Y | Y | Y | Y | ☺ |
| Nordström, 2021 [39] | ? | Y | Y | ? | ☺ | ? | Y | ? | ☺ | Y | Y | ☺ | ☺ | ? | Y | Y | Y | ? |
| Nygard, 2016 [40] | Y | Y | N | ☹ | ☺ | ? | Y | ? | ☺ | Y | ? | ? | ☺ | Y | Y | Y | N | ☹ |
| O´Malley, 2017 [41] | ? | Y | Y | ? | ☺ | Y | N | ☹ | ☺ | Y | Y | ☺ | ☺ | Y | Y | Y | N | ☹ |
| Park, 2018 [42] | Y | Y | Y | ☺ | ☺ | Y | N | ☹ | ☺ | Y | Y | ☺ | ☺ | Y | Y | Y | N | ☹ |
| Punnen, 2018 [43] | ? | Y | Y | ? | ☺ | Y | N | ☹ | ☺ | Y | Y | ☺ | ☺ | Y | Y | Y | N | ☹ |
| Roumiguié, 2020 [44] | ? | Y | Y | ? | ☺ | Y | Y | ☺ | ☺ | Y | ? | ? | ☺ | Y | Y | Y | Y | ☺ |
| Ruffion, 2013 [45] | Y | Y | ? | ? | ☺ | ? | N | ☹ | ☺ | Y | ? | ? | ☺ | Y | Y | Y | N | ☹ |
| Ruffion, 2014 [46] | Y | Y | Y | ☺ | ☺ | Y | N | ☹ | ☺ | Y | Y | ☺ | ☺ | Y | Y | Y | Y | ☺ |
| Sanchis-Bonet, 2018 [47] | ? | Y | Y | ? | ☺ | Y | N | ☹ | ☺ | Y | Y | ☺ | ☺ | Y | Y | Y | Y | ☺ |
| Sanda, 2017^8^ [48] | ? | Y | Y | ? | ☺ | Y | N | ☹ | ☺ | Y | ? | ? | ☺ | Y | Y | Y | Y | ☺ |
| Sanda, 2017^4^ [48] | ? | Y | Y | ? | ? | Y | Y | ☺ | ☺ | Y | ? | ? | ☺ | Y | Y | Y | Y | ☺ |
| Schulze, 2020 [49] | Y | Y | Y | ☺ | ☺ | ? | N | ☹ | ☺ | Y | ? | ? | ☺ | Y | Y | Y | Y | ☺ |
| Seisen, 2015 [50] | Y | Y | Y | ☺ | ☺ | Y | Y | ☺ | ☺ | Y | Y | ☺ | ☺ | Y | Y | Y | N | ☹ |
| Shore, 2019 [51] | N | N | Y | ☹ | ☺ | Y | N | ☹ | ☺ | Y | ? | ? | ☺ | N | N | Y | Y | ☹ |
| Steuber, 2021 [52] | Y | Y | Y | ☺ | ☺ | Y | N | ☹ | ☺ | Y | Y | ☺ | ☺ | Y | Y | Y | Y | ☺ |
| Tan, 2017 [53] | Y | Y | Y | ☺ | ☺ | ? | N | ☹ | ☺ | Y | Y | ☺ | ☺ | Y | Y | Y | Y | ☺ |
| Tomlins, 2016 [54] | ? | Y | N | ☹ | ? | ? | Y | ? | ☺ | Y | ? | ? | ☺ | Y | Y | Y | Y | ☺ |
| Tosoian, 2017 [55] | ? | Y | Y | ? | ☺ | ? | N | ☹ | ☺ | Y | Y | ☺ | ☺ | Y | Y | Y | Y | ☺ |
| Tosoian, 2017b [56] | ? | Y | Y | ? | ☺ | ? | N | ☹ | ☺ | Y | ? | ? | ☺ | Y | Y | Y | Y | ☺ |
| Tosoian, 2021^8^ [57] | ? | Y | N | ☹ | ☺ | ? | N | ☹ | ☺ | Y | ? | ? | ☺ | Y | Y | Y | Y | ☺ |
| Tosoian, 2021^4^ [57] | ? | Y | N | ☹ | ☺ | ? | Y | ? | ☺ | Y | ? | ? | ☺ | Y | Y | Y | Y | ☺ |
| Van Neste, 2016^8^ [58] | Y | Y | Y | ☺ | ☺ | Y | N | ☹ | ☺ | Y | Y | ☺ | ☺ | Y | Y | Y | Y | ☺ |
| Van Neste, 2016^4^ [58] | Y | Y | Y | ☺ | ☺ | Y | N | ☹ | ☺ | Y | Y | ☺ | ☺ | Y | Y | Y | Y | ☺ |
| Wang, 2017 [59] | ? | Y | ? | ☹ | ? | ? | N | ☹ | ☺ | Y | ? | ? | ☺ | Y | Y | Y | N | ☹ |
| Wei, 2014 [60] | Y | Y | N | ☹ | ☺ | Y | N | ☹ | ☺ | Y | Y | ☺ | ☺ | Y | Y | Y | N | ☹ |
| Woo, 2020^8^ [61] | ? | Y | Y | ? | ☺ | ? | N | ☹ | ☺ | Y | Y | ☺ | ☺ | ? | Y | Y | ? | ☹ |
| Woo, 2020^4^ [61] | ? | Y | Y | ? | ☺ | ? | Y | ? | ☺ | Y | Y | ☺ | ☺ | ? | Y | Y | ? | ☹ |
| Wu, 2019^8^[62] | ? | Y | Y | ☺ | ☺ | ? | N | ☹ | ☺ | Y | Y | ☺ | ☺ | Y | Y | Y | Y | ☺ |
| Wu, 2019^4^ [62] | ? | Y | Y | ☺ | ☺ | ? | N | ☹ | ☺ | Y | Y | ☺ | ☺ | Y | Y | Y | Y | ☺ |
| WyYock, 2020 [63] | Y | Y | ? | ? | ? | Y | Y | ☺ | ☺ | Y | ? | ? | ☺ | Y | N | Y | N | ☹ |
| Yu, 2016 [64] | Y | Y | Y | ☺ | ? | ? | N | ☹ | ☺ | Y | ? | ? | ☺ | Y | Y | Y | Y | ☺ |
| Zappala, 2017 [65] | Y | Y | Y | ☺ | ☺ | Y | Y | ☺ | ☺ | Y | Y | ☺ | ☺ | Y | Y | Y | Y | ☺ |

Note: AP: Aplicability concers, RoB: risk of bias; Y: yes; N: no; ?: unclear; ☺: low; ☹: high; ^1^: European cohort; ^2^: Asian cohort; ^3^: Primary cohort; ^4^: Validation cohort; ^5^: Retrospective cohort; ^6^: Prospective cohort; ^7^: Ambispective cohort; ^8^: Training cohort

Supplementary Table 4.

*Evidence profile for Prostate Health Index*

| \| Sensitivity \| 0.96 (95% CI: 0.92 to 0.98) \| \| --- \| --- \| \| Specificity \| 0.23 (95% CI: 0.15 to 0.32) \| |  | \| Prevalences \| 7.5% \| 12% \| 23% \| \| --- \| --- \| --- \| --- \| |  |
| --- | --- | --- | --- | --- | --- | --- | --- | --- | --- | --- | --- |

| Outcome | N of studies (N of patients) | Study design | Factors that may decrease certainty of evidence | | | | | Effect per 10.000 patients tested | | | Test accuracy CoE |
| --- | --- | --- | --- | --- | --- | --- | --- | --- | --- | --- | --- |
|  |  |  | Risk of bias | Indirectness | Inconsistency | Imprecision | Publication bias | Pre-test probability of 7.5% | Pre-test probability of 12% | Pre-test probability of 23% |  |
| **True positives** (patients with clinically significant prostate cancer) | 4 studies [11,24,25,31] 581 patients | cross-sectional (cohort type accuracy study) | serious^a^ | not serious | serious^b^ | not serious | none | 720  (690 to 735) | 1,152  (1,104 to 1,176) | 2,208  (2,116 to 2,254) | ⨁⨁◯◯ Low |
| **False negatives** (patients incorrectly classified as not having clinically significant prostate cancer) |  |  |  |  |  |  |  | 30  (15 to 60) | 48  (24 to 96) | 92  (46 to 184) |  |
| **True negatives** (patients without clinically significant prostate cancer) | 4 studies [11,24,25,31] 2401 patients | cross-sectional (cohort type accuracy study) | serious^a^ | not serious | Serious^b^ | not serious | none | 2,128  (1,388 to 2960) | 2,024  (1,320 to 2,816) | 1,771  (1,155 to 2,464) | ⨁⨁◯◯ Low |
| **False positives** (patients incorrectly classified as having clinically significant prostate cancer) |  |  |  |  |  |  |  | 7,122  (6,290 to 7,862) | 6,776  (5,984 to 7,480) | 5,929  (5,236 to 6,545) |  |

a. A high risk of bias owing to the absence of prespecified cut-off values or not including all participants in the analysis

b. Inconsistency was defined by an I^2^≥ 50%, P-value <0.10, unexplained by subgroup analysis.

Note. CoE: Class of Evidence.

Supplementary Table 5.

*Evidence profile for* SelectMDx

| \| Sensitivity \| 0.84 (95% CI: 0.71 to 0.92) \| \| --- \| --- \| \| Specificity \| 0.49 (95% CI: 0.26 to 0.72) \| |  | \| Prevalences \| 7.5% \| 12% \| 23% \| \| --- \| --- \| --- \| --- \| |  |
| --- | --- | --- | --- | --- | --- | --- | --- | --- | --- | --- | --- |

| Outcome | N of studies  (N of patients) | Study design | Factors that may decrease certainty of evidence | | | | | Effect per 10.000 patients tested | | | Test accuracy CoE |
| --- | --- | --- | --- | --- | --- | --- | --- | --- | --- | --- | --- |
|  |  |  | Risk of bias | Indirectness | Inconsistency | Imprecision | Publication bias | Pre-test probability of 7.5% | Pre-test probability of 12% | Pre-test probability of 23% |  |
| **True positives** (patients with clinically significant prostate cancer) | 5 studies [6,22,44,51,63] 332 patients | cross-sectional (cohort type accuracy study) | serious^a^ | not serious | serious^b^ | not serious | none | 630  (533 to 690) | 1,008  (852 to 1,104) | 1,932  (1,633 to 2,116) | ⨁⨁◯◯ Low |
| **False negatives** (patients incorrectly classified as not having clinically significant prostate cancer) |  |  |  |  |  |  |  | 120  (60 to 217) | 192  (96 to 348) | 368  (184 to 667) |  |
| **True negatives** (patients without clinically significant prostate cancer) | 5 studies [6,22,44,51,63] 910 patients | cross-sectional (cohort type accuracy study) | serious^a^ | not serious | serious^b^ | not serious | none | 4,533  (2405 to 6660) | 4,312  (2288 to 6336) | 3,773  (2,002 to 5,544) | ⨁⨁◯◯ Low |
| **False positives** (patients incorrectly classified as having clinically significant prostate cancer) |  |  |  |  |  |  |  | 4,717  (2,590 to 6,845) | 4,488  (2,464 to 6,512) | 3927  (2,156 to 5,698) |  |

a. A high risk of bias owing to the absence of prespecified cut-off values or not including all participants in the analysis

b. Inconsistency was defined by an I^2^≥ 50%, P-value <0.10, unexplained by subgroup analysis.

Note. CoE: Class of Evidence.

Supplementary Table 6.

Results on diagnostic accuracy of the included studies.

| **Study** | **Cut-off** | **TP** | **FP** | **TN** | **FN** | **S**  **(%)** | **E**  **(%)** | **PPV**  **(%)** | **VPN**  **(%)** | **AUC** |
| --- | --- | --- | --- | --- | --- | --- | --- | --- | --- | --- |
| **URINE TEST** | | | | | | | | | | |
| **Progensa® PCA3** | | | | | | | | | | |
| Cao, 2018 [7] | 30 | NR | NR | NR | NR | NR | NR | NR | NR | 0.72 |
| Hansen, 2013 [23] | 15 | 137 | NR | NR | 1 | 99.3 | NR | NR | 99 | 0.73 |
|  | 20 | 136 | NR | NR | 2 | 98.5 | NR | NR | 99 |  |
|  | 25 | 136 | NR | NR | 2 | 98.5 | NR | NR | 99 |  |
|  | 30 | 135 | NR | NR | 3 | 98 | NR | NR | 99 |  |
|  | 35 | 130 | NR | NR | 8 | 94 | NR | NR | 97 |  |
|  | 40 | 127 | NR | NR | 11 | 92 | NR | NR | 96 |  |
| Kotova, 2020 [26] | 78.61 | 19 | 94 | 1 | 39 | 33 | 0.768 | 16.78 | 1.86 | 0.63 |
| Nygard, 2016 [40] | 35^1^ | 14 | 46 | 61 | 3 | 82 | 57 | 23 | 95 | 0.78 |
|  | 35^2^ | 35 | 26 | 50 | 14 | 71 | 66 | 58 | 78 | 0.83 |
| O´Malley, 2017 [41] | NR^3^ | NR | NR | NR | NR | NR | NR | NR | NR | 0.77 |
|  | NR^4^ | NR | NR | NR | NR | NR | NR | NR | NR | 0.66 |
| Ruffion, 2014 [46] | 5 | 124 | 428 | 42 | 1 | 99 | 9 | 22.44 | 97 | NR |
|  | 10 | 123 | 357 | 113 | 3 | 98 | 24 | 25.54 | 98 | NR |
|  | 15 | 116 | 296 | 174 | 9 | 93 | 37 | 28.19 | 94 | NR |
|  | 20 | 111 | 230 | 240 | 14 | 89 | 51 | 32.57 | 93 | NR |
| Sanda, 2017 [48] | 6.3^5^ | 149 | NR | NR | 7 | 95.5 | NR | 29.2 | 91.3 | NR |
|  | 7^6^ | 143 | NR | NR | 5 | 96.6 | NR | 29.8 | 93.8 | NR |
| Seisen, 2015 [50] | 35 | 24 | 41 | 58 | 15 | 61.5 | 58.6 | 36.9 | 79.5 | 59.4 |
| Van Neste, 2016 [58] | NR | NR | NR | NR | NR | NR | NR | NR | NR | 0.80 |
| Wang, 2017 [59] | NR | NR | NR | NR | NR | NR | NR | NR | NR | 0.71 |
| Wei, 2014 [60] | NR^5^ | NR | NR | NR | NR | NR | NR | NR | NR | 0.78 |
|  | NR^6^ | NR | NR | NR | NR | NR | NR | NR | NR | 0.79 |
| Woo, 2020 [61] | NR^5^ | NR | NR | NR | NR | NR | NR | NR | NR | 0.79 |
|  | NR^6^ | NR | NR | NR | NR | NR | NR | NR | NR | 0.62 |
| **SelectMDx** | | | | | | | | | | |
| Busetto , 2020 [6] | NR | 7 | 12 | 33 | 0 | 100 | 73.3 | 36.8 | 100 | NR |
| Haese, 2019 [22] | NR | 135 | 265 | 298 | 17 | 89 | 53 | 34 | 95 | 0.80 |
| Roumiguié, 2020 [44] | NR | 20 | 60 | 33 | 4 | 84.4 | 35.3 | 25.19 | 89.76 | 0.67 |
| Shore, 2019 [89] | NR | 19 | 52 | 7 | 2 | 90.48 | 11.86 | NR | NR | NR |
| Wysock , 2020 [63] | NR | 12 | 7 | 21 | 10 | 54.5 | 75 | 63.14 | 67.72 | 0.67 |
| **TMPRSS2:ERG** | | | | | | | | | | |
| O'Malley,2017 [41] | NR^5^ | NR | NR | NR | NR | NR | NR | NR | NR | 0.73 |
|  | NR^6^ | NR | NR | NR | NR | NR | NR | NR | NR | 0.64 |
| Tomlins, 2016 [54] | NR | NR | NR | NR | NR | NR | NR | NR | NR | 0.75 |
| **MyProstateScore** | | | | | | | | | | |
| Tomlins, 2016 [54] | NR | NR | NR | NR | NR | NR | NR | NR | NR | 0.77 |
| Tosoian, 2021 [57] | ≤10^5^ | 187 | 513 | 272 | 5 | 97.4 | 34.6 | 26.7 | 98.2 | NR |
|  | >10^5^ | 186 | 524 | 261 | 6 | 96.7 | 33.3 | 26.1 | 97.7 | NR |
|  | ≤10^6^ | 141 | 287 | 115 | 5 | 96.6 | 28.6 | 32.9 | 95.8 | NR |
|  | >10^6^ | 139 | 285 | 117 | 7 | 95.5 | 29 | 30.8 | 95.2 | NR |
| Woo, 2020 [61] | NR^5^ | NR | NR | NR | NR | NR | NR | NR | NR | 0.80 |
|  | NR^6^ | NR | NR | NR | NR | NR | NR | NR | NR | 0.65 |
| ExoDx Prostate IntelliScore | | | | | | | | | | |
| McKiernan, 2016 [32] | 15.6^5^ | 76 | 128 | 49 | 2 | 97.44 | 27.68 | 37.25 | 96.08 | 0.78 |
|  | 15.62^6^ | 136 | 245 | 126 | 12 | 91.89 | 33.97 | 35.7 | 91.30 | 0.73 |
| **BLOOD TEST** | | | | | | | | | | |
| **PHI** | | | | | | | | | | |
| Abrate, 2015 [1] | 35.6 | NR | NR | NR | NR | NR | NR | NR | NR | 0.89 |
| Babajide, 2021 [2] | 27 | 52 | 218 | 23 | 0 | 100 | 9.4 | 19.23 | 100 | 0.63 |
|  | 28 | 52 | 216 | 25 | 0 | 100 | 10.4 | 19.41 | 100 |  |
|  | 28.6 | 51 | 216 | 25 | 1 | 98.1 | 10.4 | 19.11 | 96.21 |  |
|  | 29 | 50 | 216 | 25 | 2 | 96.2 | 10.4 | 18.81 | 92.69 |  |
|  | 30 | 49 | 205 | 36 | 3 | 94.2 | 15.1 | 19.32 | 92.35 |  |
|  | 31 | 48 | 195 | 46 | 4 | 92.3 | 18.9 | 19.72 | 91.92 |  |
|  | 32 | 48 | 193 | 48 | 4 | 92.3 | 19.8 | 19.89 | 92.26 |  |
|  | 33 | 47 | 189 | 52 | 5 | 90.4 | 21.7 | 19.94 | 91.29 |  |
|  | 34 | 47 | 187 | 54 | 5 | 90.4 | 22.6 | 20.13 | 91.60 |  |
|  | 35 | 47 | 178 | 63 | 5 | 90.4 | 26.2 | 20.90 | 92.67 |  |
| Barisiene, 2020 [3] | 44.71 | 30 | 47 | 123 | 10 | 75 | 72.4 | 39.0 | 92.5 | 0.77 |
|  | 33.2 | 36 | 111 | 59 | 4 | 90 | 34.7 | 4.5 | 93.7 |  |
|  | 44.71 | 56 | 24 | 105 | 25 | 69.1 | 81.4 | 70 | 80.8 |  |
|  | 31.92 | 73 | 83 | 46 | 8 | 90 | 35.7 | 46.8 | 85.2 |  |
| Bertok, 2020 [4] | NA | NR | NR | NR | NR | NR | NR | NR | NR | 0.57 |
| Boegemann, 2016 [5] | NA | NR | NR | NR | NR | NR | NR | NR | NR | 0.72 |
| Catalona, 2011 [8] | 21.3 | 132 | 633 | 120 | 7 | 95 | 16 | 17.27 | 94.55 | 0.72 |
| Chiu, 2019 [11] | 25 | 114 | 349 | 39 | 1 | 99 | 10 | 24.59 | 97.12 | NR |
|  | 35 | 108 | 244 | 144 | 7 | 94 | 37 | 30.66 | 95.41 |  |
|  | 45 | 90 | 159 | 229 | 25 | 78 | 59 | 36.06 | 90.05 |  |
|  | 25 | 63 | 664 | 374 | 3 | 96 | 36 | 8.71 | 99.30 |  |
|  | 35 | 54 | 270 | 768 | 12 | 82 | 74 | 16.70 | 98.48 |  |
|  | 55 | 18 | 42 | 996 | 48 | 27 | 96 | 30.03 | 95.39 |  |
|  | 32 | 109 | 279 | 109 | 6 | 95 | 28 | 28.11 | 94.97 |  |
|  | 40 | 104 | 202 | 186 | 12 | 90 | 48 | 33.91 | 94.18 |  |
|  | 55 | 61 | 109 | 279 | 54 | 53 | 72 | 35.94 | 83.79 |  |
|  | 30 | 59 | 426 | 612 | 7 | 89 | 59 | 12.13 | 98.83 |  |
|  | 45 | 36 | 83 | 955 | 30 | 55 | 92 | 30.42 | 96.98 |  |
| Chiu, 2016 [9] | 35 | NR | NR | NR | NR | NR | NR | NR | NR | 0.84 |
| Chiu, 2016 [10] | 35 | NR | NR | NR | NR | NR | NR | NR | NR | 0.85 |
| Choi, 2020 [12] | PHI ≥27.0 + PSA ≥2.5 | 3 | 75 | 11 | 25 | 10 | 13 | 3.61 | 30.73 | NR |
|  | PHI ≥36.0 + PSA ≥2.5 | 25 | 61 | 25 | 3 | 89 | 29 | 28.98 | 89.01 |  |
|  | PHI ≥55.0 + PSA ≥2.5 | 12 | 24 | 62 | 16 | 43 | 72 | 33.33 | 79.51 |  |
|  | PHI ≥36.0 + PSA ≥4 | 17 | 43 | 43 | 11 | 61 | 50 | 28.43 | 79.75 |  |
|  | PHI ≥36.0 + PSAD ≥0.18 | 14 | 21 | 65 | 14 | 50 | 76 | 40.42 | 82.36 |  |
|  | PHI ≥36.0 +PSAD ≥0.18 + %free PSA ≤13 | 12 | 18 | 68 | 16 | 43 | 79 | 40.00 | 80.98 |  |
|  | PHI ≥40.7 + PSAD ≥0.1 + %free PSA =2.87 | NR | NR | NR | NR | NR | NR | NR | NR | 0.68 |
| de la Calle, 2015 [13] | 34.3^5^ | 91 | 42 | 77 | 23 | 80 | 64.8 | 68.53 | 77.18 | 0.82 |
|  | 27.5^5^ | 103 | 65 | 54 | 11 | 90 | 45.2 | 61.14 | 82.51 |  |
|  | 24.4^5^ | 108 | 76 | 43 | 6 | 95 | 36 | 58.71 | 88.26 |  |
|  | 24^6^ | 112 | 191 | 82 | 10 | 92 | 30 | 37.00 | 89.35 | 0.78 |
| Fan, 2019 [16] | 36.96 | 47 | 122 | 133 | 5 | 90 | 52.1 | 27.70 | 96.23 |  |
|  | 27 | 52 | 216 | 39 | 0 | 100 | 15.3 | 19.40 | 100 |  |
|  | 36 | 48 | 139 | 116 | 4 | 92.3 | 45.5 | 25.67 | 96.66 |  |
|  | 55 | 22 | 19 | 236 | 30 | 42.3 | 92.5 | 53.49 | 88.72 |  |
| Filella, 2014 [17] | NA | NR | NR | NR | NR | NR | NR | NR | NR | 0.87 |
| Foj, 2020 [18] | NA | NR | NR | NR | NR | NR | NR | NR | NR | 0.77 |
| Foley, 2016 [19] | NA | NR | NR | NR | NR | NR | NR | NR | NR | 0.78 |
| Furuya, 2017 [20] | 30.7 | 18 | 19 | 10 | 3 | 85.7 | 34.5 | 48.6 | 76.9 | 0.79 |
| Guazzo, 2011 [21] | NA | NR | NR | NR | NR | NR | NR | NR | NR | 0.72 |
| Hsieh, 2020 [24] | 25 | 23 | 60 | 18 | 1 | 95.8 | 23.1 | 27.7 | 94.7 | 0.74 |
|  | 30 | 22 | 44 | 34 | 2 | 91.7 | 43.6 | 33.3 | 94.4 |  |
|  | 35 | 19 | 38 | 40 | 5 | 79.2 | 51.3 | 33.3 | 88.9 |  |
|  | 40 | 17 | 34 | 44 | 7 | 70.8 | 56.4 | 33.3 | 33.3 |  |
| Kim, 2020 [25] | 20 | 255 | 258 | 29 | 3 | 99 | 10 | 49.72 | 90 | NR |
|  | 25 | 248 | 215 | 72 | 10 | 96 | 25 | 53.50 | 87 |  |
|  | 30 | 237 | 172 | 115 | 21 | 92 | 40 | 57.95 | 85 |  |
|  | 35 | 224 | 129 | 158 | 34 | 87 | 55 | 63.48 | 83 |  |
|  | 20 | 176 | 339 | 30 | 0 | 100 | 8 | 34.14 | 100 |  |
|  | 25 | 174 | 288 | 81 | 2 | 99 | 22 | 37.71 | 99 |  |
|  | 30 | 167 | 240 | 129 | 9 | 95 | 35 | 41.08 | 94 |  |
|  | 35 | 164 | 188 | 181 | 12 | 93 | 49 | 46.52 | 93 |  |
| Lazzeri, 2016 [28] | NA | NR | NR | NR | NR | NR | NR | NR | NR | 0.84 |
| Lazzeri, 2013 [27] | 27.6 | NR | NR | NR | NR | NR | NR | NR | NR | 0.65 |
| Loeb, 2017 [31] | 15 | 118 | 601 | 9 | 0 | 99.7 | 1.5 | 16.37 | 98.1 |  |
|  | 16 | 117 | 595 | 15 | 1 | 99.2 | 2.5 | 16.44 | 96.8 |  |
|  | 17 | 116 | 588 | 22 | 2 | 98.3 | 3.6 | 16.48 | 95.7 |  |
|  | 18 | 115 | 575 | 35 | 3 | 97.5 | 5.7 | 16.67 | 95.9 |  |
|  | 19 | 115 | 566 | 44 | 3 | 97.5 | 7.2 | 16.89 | 96.7 |  |
|  | 20 | 115 | 548 | 62 | 3 | 97.5 | 10.2 | 17.36 | 97.6 |  |
|  | 21 | 114 | 538 | 72 | 4 | 96.6 | 11.8 | 17.48 | 97.3 |  |
|  | 22 | 112 | 511 | 99 | 6 | 94.9 | 16.2 | 17.97 | 97.1 |  |
|  | 23 | 111 | 488 | 122 | 7 | 94.1 | 20 | 18.54 | 97.2 |  |
|  | 24 | 110 | 468 | 142 | 8 | 93.2 | 23.3 | 19.03 | 97.3 |  |
|  | 25 | 107 | 454 | 156 | 11 | 90.7 | 25.6 | 19.08 | 96.6 |  |
|  | 26 | 106 | 435 | 175 | 12 | 89.8 | 28.7 | 19.59 | 96.7 |  |
|  | 27 | 105 | 408 | 202 | 13 | 89 | 33.1 | 20.47 | 96.9 |  |
|  | 28 | 103 | 387 | 223 | 15 | 87.3 | 36.6 | 21.03 | 96.8 |  |
|  | 29 | 98 | 368 | 242 | 20 | 83.1 | 39.7 | 21.05 | 96 |  |
|  | 30 | 95 | 348 | 262 | 23 | 80.5 | 43 | 21.46 | 95.8 |  |
|  | 31 | 92 | 325 | 285 | 26 | 78 | 46.7 | 22.06 | 95.6 |  |
|  | 32 | 89 | 305 | 305 | 29 | 75.4 | 50 | 22.58 | 95.5 |  |
|  | 33 | 89 | 291 | 319 | 29 | 75.4 | 52.3 | 23.42 | 95.7 |  |
|  | 34 | 87 | 269 | 341 | 31 | 73.7 | 55.9 | 24.43 | 95.7 |  |
|  | 35 | 81 | 249 | 361 | 37 | 68.6 | 59.2 | 24.54 | 95.1 |  |
| Loeb, 2015 [30] | 33.8^7^ | 128 | 271 | 227 | 32 | 80 | 45.5 | 32.05 | 87.63 | 0.71 |
|  | 31^7^ | 136 | 313 | 185 | 24 | 85 | 37.2 | 30.31 | 88.53 |  |
|  | 28. 6^7^ | 144 | 348 | 150 | 16 | 90 | 30.1 | 29.26 | 90.36 |  |
|  | 24.1^7^ | 152 | 417 | 81 | 8 | 95 | 16.3 | 26.72 | 91.03 |  |
|  | 34.4^8^ | 87 | 294 | 255 | 22 | 80 | 46.4 | 22.86 | 92.12 | 0.70 |
|  | 31.9^8^ | 93 | 335 | 214 | 16 | 85 | 38.9 | 21.64 | 92.89 |  |
|  | 28.9^8^ | 98 | 386 | 163 | 11 | 90 | 29.7 | 20.27 | 93.73 |  |
|  | 28.1^8^ | 104 | 399 | 150 | 5 | 95 | 27.4 | 20.62 | 96.50 |  |
| MeariNR, 2014 [33] | NA | NR | NR | NR | NR | NR | NR | NR | NR | 0.6 |
| Morote, 2016 [34] | 17.83 | 43 | 104 | 34 | 2 | 95 | 24.4 | 29.5 | 94 | 0.79 |
|  | 27.99 | 45 | 129 | 9 | 0 | 100 | 6.5 | 25.9 | 100 |  |
| Na, 2017 [37] | 31 | 439 | 421 | 629 | 49 | 90 | 59.9 | 51.05 | 92.80 | 0.91 |
|  | 18 | 487 | 909 | 141 | 1 | 99.8 | 13.43 | 34.89 | 99.31 |  |
|  | 24 | 484 | 749 | 301 | 4 | 99.18 | 28.67 | 39.25 | 98.69 |  |
|  | 28 | 482 | 648 | 402 | 6 | 98.77 | 38.29 | 42.66 | 98.53 |  |
|  | 32 | 476 | 547 | 503 | 12 | 97.54 | 47.9 | 46.53 | 97.67 |  |
|  | 35 | 470 | 469 | 581 | 18 | 96.31 | 55.33 | 50.05 | 96.99 |  |
|  | 18 o PSA= 10 ng/ml | 487 | 909 | 141 | 1 | 99.8 | 13.43 | 34.89 | 99.31 |  |
|  | 24 o PSA= 10 ng/ml | 488 | 818 | 232 | 0 | 100 | 22.1 | 37.37 | 100 |  |
|  | 28 o PSA= 10 ng/ml | 488 | 741 | 309 | 0 | 100 | 29.43 | 39.71 | 100 |  |
|  | 32 o PSA=10 ng/ml | 486 | 671 | 379 | 2 | 99.59 | 36.1 | 42.01 | 99.47 |  |
|  | 35 o PSA= 10 ng/ml | 482 | 626 | 424 | 6 | 98.77 | 40.38 | 43.50 | 98.60 |  |
| Na, 2014 [36] | NA | NR | NR | NR | NR | NR | NR | NR | NR | 0.85 |
| Nordström, 2015 [38] | NA | NR | NR | NR | NR | NR | NR | NR | NR | 0.71 |
| Park, 2018 [42] | NA | NR | NR | NR | NR | NR | NR | NR | NR | 0.97 |
| Sanchis-Bonet, 2018 [47] | NA | NR | NR | NR | NR | NR | NR | NR | NR | 0.66 |
| Schulze, 2020 [49] | 40 | NR | NR | NR | NR | NR | NR | NR | NR | 0.76 |
| Seisen, 2015 [50] | 40 | 26 | 26 | 73 | 13 | 66.7 | 73.7 | 50 | 84.9 | 71.7 |
| Tan, 2017 [53] | 26.75 | 17 | 274 | 336 | 2 | 90 | 55.1 | 5.88 | 99.44 | NR |
| Tosoian, 2017 [56] | NA | NR | NR | NR | NR | NR | NR | NR | NR | 0.76 |
| Tosoian, 2017 [55] | NA | NR | NR | NR | NR | NR | NR | NR | NR | 0.77 |
| Wu, 2019 [62] | NA | NR | NR | NR | NR | NR | NR | NR | NR | 0.89 |
|  | NA | NR | NR | NR | NR | NR | NR | NR | NR | 0.92 |
| Yu, 2016 [64] | 38.59 | NR | NR | NR | NR | NR | NR | NR | NR | 0.86 |
| **PHI Density** | | | | | | | | | | |
| Babajide, 2021 [2] | NA | NR | NR | NR | NR | NR | NR | NR | NR | 0.74 |
| Barisiene, 2020 [3] | 0.63 | 30 | 57 | 113 | 10 | 75 | 66.5 | 34.5 | 91.9 | 0.75 |
|  | 0.63 | 58 | 28 | 101 | 23 | 71.6 | 78.3 | 67.4 | 81.5 | 0.75 |
| Druskin, 2018 [14] | 0.44^9^ | 84 | 97 | 53 | 7 | 92.3 | 35.3 | 46.4 | 88.3 | 0.78 |
|  | 0.44^8^ | 66 | 115 | 55 | 5 | 93 | 32.4 | 36.5 | 91.7 | 0.78 |
|  | 0.44^10^ | 37 | 144 | 59 | 1 | 97.4 | 29.1 | 20.4 | 98.3 | 0.78 |
| Mearini, 2014 [33] | NA | NR | NR | NR | NR | NR | NR | NR | NR | 0.59 |
| Schulze, 2020 [49] | ≥0.9 | NR | NR | NR | NR | NR | NR | NR | NR | 0.764 |
| Tosoian, 2017 [56] | 0.43 | 34 | 51 | 32 | 1 | 97.9 | 38 | 39.97 | 96.4 | 0.84 |
| **4Kscore® test** | | | | | | | | | | |
| Falagario, 2021 [15] | NA | NR | NR | NR | NR | NR | NR | NR | NR | 0.87 |
| Punnen, 2018 [43] | NA | NR | NR | NR | NR | NR | NR | NR | NR | 0.72 |
| Nordström, 2015 [38] | NA | NR | NR | NR | NR | NR | NR | NR | NR | 0.72 |
| Wysock, 2020 [63] | 7.5 | 21 | 19 | 9 | 1 | 95.5 | 32.1 | 52.50 | 90.08 | 0.83 |
|  | 12 | 20 | 13 | 15 | 2 | 90.1 | 53.5 | 60.36 | 87.31 | 0.83 |
| Zappala, 2017 [65] | 7.5 | NR | NR | NR | NR | NR | NR | NR | NR | 0.82 |
|  | 7.5 | NR | NR | NR | NR | NR | NR | NR | NR | 0.82 |
| **Stockholm3 test** | | | | | | | | | | |
| Nordström, 2021 [39] | NR | NR | NR | NR | NR | NR | NR | NR | NR | 0.77 |
| Mortezavi, 2020 [35] | Predetermined | NR | NR | NR | NR | NR | NR | NR | NR | 0.86 |
| **Proclarix® Test** | | | | | | | | | | |
| Steuber, 2021 [52] | 10 | 62 | 229 | 65 | 6 | 91 | 22 | NR | NR | NR |

Note: AUC: Area under the curve; E: Especificity; FN: False negative; FP: False positive; NPV: negative predictive value; NA: not applicable; NR: Not Reported; PHI: Prostate Health Index; PPV: positive predictive value; PSA: Prostate-specific antigen; PSAD: Prostate-specific antigen density; S: sensitivity; TN: true negative; TP: true positive

1 High risk according to the European Association of Urology; 2 Moderate and high risk according to the European Association of Urology; 3 Afroamericans; 4 No afroamericans; 5 Training Cohort; 6 Validation Cohort; 7 High risk according to the Epstein´s criteria; 8 Gleason ≥7 or ISUP Grade Group ≥ 2; ISUP Grade Group = 1 in >2 cores or >50% of any one core or ISUP Grade Group ≥ 2; 10 ISUP Grade Group ≥ 3

Supplementary Table 7.

Summary of meta-analysis results

| **Biomarker**  ***Cut-off***  Origin | **K** | **N** | **Sensitivity [IC95%]** | **I^2^** | **Specificity [IC95%]** | **I^2^** | **LR+** | **LR-** | **AUC** | **P-value cov** | **Bias**  **[P-value]** |
| --- | --- | --- | --- | --- | --- | --- | --- | --- | --- | --- | --- |
| **Prostate Health Index (PHI)** | | | | | | | | | | | |
| ***15-20*** | 4 | 2994 | 0.99 [0.97, 1.00] | 76.26 | 0.14 [0.09, 0.19] | 87.03 | 1.10 [1.10, 1.20] | 0.09 [0.03, 0.25] | 0.53 [0.49, 0.57] | NA | 0.20 |
| Asians | 1 | 1556 | 1.00 [0.99, 1.00] |  | 0.13 [0.04, 0.22] |  |  |  |  | 0.05 |  |
| Europeans / Caucasians | 3 | 1428 | 0.98 [0.96, 1.00] |  | 0.13 [0.08, 0.19] |  |  |  |  |  |  |
| ***20-25*** | 7 | 6698 | 0.96 [0.94, 0.98] | 73.26 | 0.24 [0.18, 0.30] | 95.57 | 1.30 [1.20, 1.30] | 0.16 [0.10, 0.27] | 0.71 [0.67, 0.75] | NA | 0.07 |
| Asians | 3 | 2744 | 0.99 [0.97, 1.00] |  | 0.30 [0.20, 0.39] |  |  |  |  | 0.01 |  |
| Europeans / Caucasians | 4 | 3954 | 0.95 [0.93, 0.97] |  | 0.21 [0.16, 0.26] |  |  |  |  |  |  |
| ***25-30*** | 9 | 6321 | 0.95 [0.89, 0.98] | 85.72 | 0.33 [0.23, 0.45] | 98.27 | 1.40 [1.20, 1.60] | 0.16 [0.09, 0.28] | 0.76 [0.72, 0.79] | NA | 0.06 |
| Afro-Americans | 1 | 293 | 0.94 [0.83, 1.00] |  | 0.15 [0.05, 0.34] |  |  |  |  | <0.01 |  |
| Asians | 5 | 3680 | 0.98 [0.94, 1.00] |  | 0.41 [0.25, 0.58] |  |  |  |  |  |  |
| Europeans / Caucasians | 4 | 2348 | 0.93 [0.87, 1.00] |  | 0.30 [0.15, 0.44] |  |  |  |  |  |  |
| ***30-35*** | 9 | 5964 | 0.87 [0.81, 0.91] | 88.94 | 0.49 [0.41, 0.58] | 97.53 | 1.70 [1.50, 2.00] | 0.26 [0.18, 0.38] | 0.76 [0.72, 0.80] | NA | 0.02 |
| Afro-Americans | 1 | 293 | 0.91 [0.78, 1.00] |  | 0.26 [0.11, 0.41] |  |  |  |  | <0.01 |  |
| Asians | 4 | 2794 | 0.91 [0.84, 0.97] |  | 0.58 [0.46, 0.70] |  |  |  |  |  |  |
| Europeans / Caucasians | 5 | 2877 | 0.85 [0.79, 0.91] |  | 0.49 [0.41, 0.57] |  |  |  |  |  |  |
| ***35-40*** | 5 | 1164 | 0.79 [0.66, 0.88] | 83.13 | 0.56 [0.48, 0.64] | 81.78 | 1.80 [1.50, 2.10] | 0.37 [0.23, 0.59] | 0.69 [0.64, 0.72] | NA | 0.14 |
| Asians | 3 | 523 | 0.77 [0.62, 0.92] |  | 0.54 [0.43, 0.64] |  |  |  |  | 0.43 |  |
| Europeans / Caucasians | 2 | 641 | 0.81 [0.66, 0.96] |  | 0.60 [0.48, 0.72] |  |  |  |  |  |  |
| ***25*** | 4 | 2982 | 0.96 [0.92, 0.98] | 65.14 | 0.23 [0.15, 0.32] | 97.00 | 1.20 [1.10, 1.40] | 0.18 [0.10, 0.31] | 0.74 [0.70, 0.77] | NA | 0.89 |
| Asians | 2 | 1206 | 0.97 [0.92, 1.00] |  | 0.31 [0.17, 0.44] |  |  |  |  | 0.17 |  |
| Europeans / Caucasians | 3 | 1776 | 0.97 [0.93, 1.00] |  | 0.19 [0.11, 0.26] |  |  |  |  |  |  |
| ***30*** | 4 | 2773 | 0.90 [0.84, 0.94] | 69.39 | 0.39 [0.26, 0.54] | 98.18 | 1.50 [1.20, 1.80] | 0.26 [0.17, 0.41] | 0.82 [0.79, 0.85] | NA | 0.99 |
| Afro-Americans | 1 | 293 | 0.94 [0.87, 1.00] |  | 0.15 [0.10, 0.20] |  |  |  |  | <0.001 |  |
| Asians | 2 | 1206 | 0.91 [0.84, 0.99] |  | 0.57 [0.52, 0.62] |  |  |  |  |  |  |
| Europeans / Caucasians | 2 | 1274 | 0.87 [0.80, 0.95] |  | 0.42 [0.38, 0.45] |  |  |  |  |  |  |
| ***35*** | 6 | 4813 | 0.88 [0.80, 0.93] | 93.05 | 0.51 [0.40, 0.63] | 98.19 | 1.80 [1.5, 2.30] | 0.23 [0.14, 0.38] | 0.77 [0.73, 0.81] | NA | 0.05 |
| Afro-Americans | 1 | 293 | 0.91 [0.76, 1.00] |  | 0.26 [0.13, 0.39] |  |  |  |  | <0.001 |  |
| Asians | 3 | 2744 | 0.91 [0.84, 0.99] |  | 0.64 [0.53, 0.74] |  |  |  |  |  |  |
| Europeans / Caucasians | 3 | 1776 | 0.86 [0.75, 0.97] |  | 0.50 [0.39, 0.61] |  |  |  |  |  |  |
| ***55*** | 3 | 2028 | 0.42 [0.32, 0.53] | 73.61 | 0.87 [0.72, 0.95] | 98.28 | 3.30 [1.70, 6.10] | 0.66 [0.59, 0.75] | 0.60 [0.56, 0.64] | NA | 0.97 |
| **SelectMDx** | 5 | 1957 | 0.84 [0.71, 0.92] | 79.74 | 0.49 [0.26, 0.72] | 93.88 | 1.60 [1.10, 2.50] | 0.32 [0.18, 0.57] | 0.79 [0.75, 0.82] | NA | 0.52 |

Note. ACU: area under the ROC curve; Bias: publication bias; E: Specificity; FN: false negatives; FP: False positives; K: number of studies; LR+: positive likelihood of English positive likelihood ratio; LR-: negative likelihood ratio of English; N: number of patients; NR: not reported; S: sensitivity

**References**

[1] Abrate A, Lazzeri M, Lughezzani G, Buffi N, Bini V, Haese A, et al. Clinical performance of the Prostate Health Index (PHI) for the prediction of prostate cancer in obese men: Data from the PROMEtheuS project, a multicentre European prospective study. BJU Int 2015;115:537–45. https://doi.org/10.1111/bju.12907.

[2] Babajide R, Carbunaru S, Nettey OS, Watson KS, Holloway-Beth A, McDowell T, et al. Performance of Prostate Health Index in Biopsy Naïve Black Men. J Urol 2021;205:718–24. https://doi.org/10.1097/JU.0000000000001453.

[3] Barisiene M, Bakavicius A, Stanciute D, Jurkeviciene J, Zelvys A, Ulys A, et al. Prostate Health Index and Prostate Health Index Density as Diagnostic Tools for Improved Prostate Cancer Detection. Biomed Res Int 2020;2020. https://doi.org/10.1155/2020/9872146.

[4] Bertok T, Jane E, Bertokova A, Lorencova L, Zvara P, Smolkova B, et al. Validating fpsa glycoprofile as a prostate cancer biomarker to avoid unnecessary biopsies and re-biopsies. Cancers (Basel) 2020;12:1–10. https://doi.org/10.3390/cancers12102988.

[5] Boegemann M, Stephan C, Cammann H, Vincendeau S, Houlgatte A, Jung K, et al. The percentage of prostate-specific antigen (PSA) isoform [-2]proPSA and the Prostate Health Index improve the diagnostic accuracy for clinically relevant prostate cancer at initial and repeat biopsy compared with total PSA and percentage free PSA in men. BJU Int 2016;117:72–9. https://doi.org/10.1111/bju.13139.

[6] Busetto GM, Del Giudice F, Maggi M, De Marco F, Porreca A, Sperduti I, et al. Prospective assessment of two-gene urinary test with multiparametric magnetic resonance imaging of the prostate for men undergoing primary prostate biopsy. World J Urol 2020;39:1869–77. https://doi.org/10.1007/s00345-020-03359-w.

[7] Cao L, Lee CH, Ning J, Handy BC, Wagar EA, Meng QH. Combination of prostate cancer antigen 3 and prostate-specific antigen improves diagnostic accuracy in men at risk of prostate cancer. Arch Pathol Lab Med 2018;142:1106–12. https://doi.org/10.5858/arpa.2017-0185-OA.

[8] Catalona WJ, Partin AW, Sanda MG, Wei JT, Klee GG, Bangma C, et al. A Multi-Center Study of [−2]Pro-Prostate-Specific Antigen (PSA) in Combination with PSA and Free PSA for Prostate Cancer Detection in the 2.0 to 10.0 ng/mL PSA Range William. J Urol 2011;23:1–7. https://doi.org/10.1016/j.juro.2010.12.032.A.

[9] Chiu PKF, Teoh JYC, Lee WM, Yee CH, Chan ESY, Hou SM, et al. Extended use of prostate health index and percentage of [-2]pro-prostate-specific antigen in Chinese men with prostate specific antigen 10–20 ng/mL and normal digital rectal examination. Investig Clin Urol 2016;57:336–42. https://doi.org/10.4111/icu.2016.57.5.336.

[10] Chiu PKF, Roobol MJ, Teoh JY, Lee WM, Yip SY, Hou SM, et al. Prostate health index (PHI) and prostate-specific antigen (PSA) predictive models for prostate cancer in the Chinese population and the role of digital rectal examination-estimated prostate volume. Int Urol Nephrol 2016;48:1631–7. https://doi.org/10.1007/s11255-016-1350-8.

[11] Chiu PKF, Ng CF, Semjonow A, Zhu Y, Vincendeau S, Houlgatte A, et al. A Multicentre Evaluation of the Role of the Prostate Health Index (PHI) in Regions with Differing Prevalence of Prostate Cancer: Adjustment of PHI Reference Ranges is Needed for European and Asian Settings(Figure presented.). Eur Urol 2019;75:558–61. https://doi.org/10.1016/j.eururo.2018.10.047.

[12] Choi J, Kang M, Sung HH, Jeon HG, Jeong BC, Seo S Il, et al. Correlation between gleason score distribution and prostate health index in patients with prostate-specific antigen values of 2.5–10 ng/ml. Investig Clin Urol 2020;61:582–7. https://doi.org/10.4111/icu.20200084.

[13] De La Calle C, Patil D, Wei JT, Scherr DS, Sokoll L, Chan DW, et al. Multicenter evaluation of the prostate health index to detect aggressive prostate cancer in biopsy Naïve men. J Urol 2015;194:65–72. https://doi.org/10.1016/j.juro.2015.01.091.

[14] Druskin SC, Tosoian JJ, Young A, Collica S, Srivastava A, Ghabili K, et al. Combining Prostate Health Index density, magnetic resonance imaging and prior negative biopsy status to improve the detection of clinically significant prostate cancer. BJU Int 2018;121:619–26. https://doi.org/10.1111/bju.14098.

[15] Falagario UG, Lantz A, Jambor I, Martini A, Ratnani P, Wagaskar V, et al. Using biomarkers in patients with positive multiparametric magnetic resonance imaging: 4Kscore predicts the presence of cancer outside the index lesion. Int J Urol 2021;28:47–52. https://doi.org/10.1111/iju.14385.

[16] Fan Y-HF, Po-Hsun P, Tzu-Ping L, Tzu-Hao H, Tzu-Chun W, I-Shen H, et al. Prostate Health Index outperforms other PSA derivatives in predicting a positive biopsy in men with tPSA <10 ng/mL: Largest prospective cohort in Taiwan. J Chinese Med Assoc 2019;82:772–7. https://doi.org/10.1097/JCMA.0000000000000160.

[17] Filella X, Foj L, Augé JM, Molina R, Alcover J. Clinical utility of %p2PSA and prostate health index in the detection of prostate cancer. Clin Chem Lab Med 2014;52:1347–55. https://doi.org/10.1515/cclm-2014-0027.

[18] Foj L, Filella X. Development and internal validation of a novel PHI-nomogram to identify aggressive prostate cancer. Clin Chim Acta 2020;501:174–8. https://doi.org/10.1016/j.cca.2019.10.039.

[19] Foley RW, Gorman L, Sharifi N, Murphy K, Moore H, Tuzova A V., et al. Improving multivariable prostate cancer risk assessment using the Prostate Health Index. BJU Int 2016;117:409–17. https://doi.org/10.1111/bju.13143.

[20] Furuya K, Kawahara T, Narahara M, Tokita T, Fukui S, Imano M, et al. Measurement of serum isoform [–2]proPSA derivatives shows superior accuracy to magnetic resonance imaging in the diagnosis of prostate cancer in patients with a total prostate-specific antigen level of 2–10 ng/ml. Scand J Urol 2017;51. https://doi.org/10.1080/21681805.2017.1298155.

[21] Guazzoni G, Nava L, Lazzeri M, Scattoni V, Lughezzani G, MacCagnano C, et al. Prostate-specific antigen (PSA) isoform p2PSA significantly improves the prediction of prostate cancer at initial extended prostate biopsies in patients with total PSA between 2.0 and 10 ng/ml: Results of a prospective study in a clinical setting. Eur Urol 2011;60:214–22. https://doi.org/10.1016/j.eururo.2011.03.052.

[22] Haese A, Trooskens G, Steyaert S, Hessels D, Brawer M, Vlaeminck-Guillem V, et al. Multicenter Optimization and Validation of a 2-Gene mRNA Urine Test for Detection of Clinically Significant Prostate Cancer before Initial Prostate Biopsy. J Urol 2019;202:256–62. https://doi.org/10.1097/JU.0000000000000293.

[23] Hansen J, Auprich M, Ahyai SA, De La Taille A, Van Poppel H, Marberger M, et al. Initial prostate biopsy: Development and internal validation of a biopsy-specific nomogram based on the prostate cancer antigen 3 assay. Eur Urol 2013;63:201–9. https://doi.org/10.1016/j.eururo.2012.07.030.

[24] Hsieh PF, Li WJ, Lin WC, Chang H, Chang CH, Huang CP, et al. Combining prostate health index and multiparametric magnetic resonance imaging in the diagnosis of clinically significant prostate cancer in an Asian population. World J Urol 2020;38:1207–14. https://doi.org/10.1007/s00345-019-02889-2.

[25] Kim L, Boxall N, George A, Burling K, Acher P, Aning J, et al. Clinical utility and cost modelling of the phi test to triage referrals into image-based diagnostic services for suspected prostate cancer: The PRIM (Phi to RefIne Mri) study. BMC Med 2020;18:1–9. https://doi.org/10.1186/s12916-020-01548-3.

[26] Kotova ES, Savochkina YA, Doludin Y V., Vasilyev AO, Prilepskay EA, Potoldykova N V., et al. Identification of clinically significant prostate cancer by combined PCA3 and AMACR mRNA detection in urine samples. Res Reports Urol 2020;12:403–13. https://doi.org/10.2147/RRU.S262310.

[27] Lazzeri M, Haese A, De La Taille A, Palou Redorta J, McNicholas T, Lughezzani G, et al. Serum isoform [-2]proPSA derivatives significantly improve prediction of prostate cancer at initial biopsy in a total PSA range of 2-10 ng/ml: A multicentric european study. Eur Urol 2013;63:986–94. https://doi.org/10.1016/j.eururo.2013.01.011.

[28] Lazzeri M, Lughezzani G, Haese A, McNicholas T, de la Taille A, Buffi NM, et al. Clinical performance of prostate health index in men with tPSA>10 ng/ml: Results from a multicentric European study. Urol Oncol Semin Orig Investig 2016;34:415.e13-415.e19. https://doi.org/10.1016/j.urolonc.2016.04.003.

[29] Leyten GHJM, Hessels D, Smit FP, Jannink SA, De Jong H, Melchers WJG, et al. Identification of a candidate gene panel for the early diagnosis of prostate cancer. Clin Cancer Res 2015;21:3061–70. https://doi.org/10.1158/1078-0432.CCR-14-3334.

[30] Loeb S, Sanda MG, Broyles DL, Shin SS, Bangma CH, Wei JT, et al. The prostate health index selectively identifies clinically significant prostate cancer. J Urol 2015;193:1163–9. https://doi.org/10.1016/j.juro.2014.10.121.

[31] Loeb S, Shin SS, Broyles DL, Wei JT, Sanda M, Klee G, et al. Prostate Health Index improves multivariable risk prediction of aggressive prostate cancer. BJU Int 2017;120:61–8. https://doi.org/10.1111/bju.13676.

[32] McKiernan J, Donovan MJ, O’Neill V, Bentink S, Noerholm M, Belzer S, et al. A novel urine exosome gene expression assay to predict high-grade prostate cancer at initial biopsy. JAMA Oncol 2016;2:882–9. https://doi.org/10.1001/jamaoncol.2016.0097.

[33] Mearini L, Ferri C, Lazzeri M, Bini V, Nunzi E, Fiorini D, et al. Evaluation of prostate-specific antigen isoform p2PSA and its derivates, %p2PSA, prostate health index and prostate dimension-adjusted related index in the detection of prostate cancer at first biopsy: An exploratory, prospective study. Urol Int 2014;93:135–45. https://doi.org/10.1159/000356240.

[34] Morote J, Celma A, Planas J, Placer J, Ferrer R, de Torres I, et al. Eficacia del índice de salud prostática para identificar cánceres de próstata agresivos. Una validación institucional. Actas Urol Esp 2016;40:378–85. https://doi.org/10.1016/j.acuro.2016.01.004.

[35] Mortezavi A, Palsdottir T, Eklund M, Chellappa V, Murugan SK, Saba K, et al. Head-to-head Comparison of Conventional, and Image- and Biomarker-based Prostate Cancer Risk Calculators. Eur Urol Focus 2020:1–8. https://doi.org/10.1016/j.euf.2020.05.002.

[36] Na R, Ye D, Liu F, Chen H, Qi J, Wu Y, et al. Performance of serum prostate-specific antigen isoform [-2]proPSA (p2PSA) and the prostate health index (PHI) in a Chinese hospital-based biopsy population. Prostate 2014;74:1569–75. https://doi.org/10.1002/pros.22876.

[37] Na R, Ye D, Qi J, Liu F, Helfand BT, Brendler CB, et al. Prostate health index significantly reduced unnecessary prostate biopsies in patients with PSA 2-10 ng/mL and PSA >10 ng/mL: Results from a Multicenter Study in China. Prostate 2017;77:1221–9. https://doi.org/10.1002/pros.23382.

[38] Nordström T, Vickers A, Assel M, Lilja H, Grönberg H, Eklund M. Comparison between the four-kallikrein panel and prostate health index for predicting prostate cancer. Eur Urol 2015;68:139–46. https://doi.org/10.1016/j.eururo.2014.08.010.

[39] Nordström T, Engel JC, Bergman M, Egevad L, Aly M, Eklund M, et al. Identifying Prostate Cancer Among Men with Lower Urinary Tract Symptoms. Eur Urol Open Sci 2021;24:11–6. https://doi.org/10.1016/j.euros.2020.12.004.

[40] Nygård Y, Haukaas SA, Halvorsen OJ, Gravdal K, Frugård J, Akslen LA, et al. A positive Real-Time Elastography (RTE) combined with a Prostate Cancer Gene 3 (PCA3) score above 35 convey a high probability of intermediate- or high-risk prostate cancer in patient admitted for primary prostate biopsy. BMC Urol 2016;16:1–8. https://doi.org/10.1186/s12894-016-0159-1.

[41] O’Malley PG, Nguyen DP, Al Hussein Al Awamlh B, Wu G, Thompson IM, Sanda M, et al. Racial Variation in the Utility of Urinary Biomarkers PCA3 and T2ERG in a Large Multicenter Study. J Urol 2017;198:42–9. https://doi.org/10.1016/j.juro.2017.01.058.

[42] Park H, Lee SW, Song G, Kang TW, Jung JH, Chung HC, et al. Diagnostic performance of %[-2]proPSA and prostate health index for prostate cancer: Prospective, multi-institutional study. J Korean Med Sci 2018;33:1–10. https://doi.org/10.3346/jkms.2018.33.e94.

[43] Punnen S, Freedland SJ, Polascik TJ, Loeb S, Risk MC, Savage S, et al. A Multi-Institutional Prospective Trial Confirms Noninvasive Blood Test Maintains Predictive Value in African American Men. J Urol 2018;199:1459–63. https://doi.org/10.1016/j.juro.2017.11.113.

[44] Roumiguié M, Ploussard G, Nogueira L, Bruguière E, Meyrignac O, Lesourd M, et al. Independent evaluation of the respective predictive values for high-grade prostate cancer of clinical information and RNA biomarkers after upfront MRI and image-guided biopsies. Cancers (Basel) 2020;12:1–12. https://doi.org/10.3390/cancers12020285.

[45] Ruffion A, Devonec M, Champetier D, Decaussin-Petrucci M, Rodriguez-Lafrasse C, Paparel P, et al. PCA3 and PCA3-based nomograms improve diagnostic accuracy in patients undergoing first prostate biopsy. Int J Mol Sci 2013;14:17767–80. https://doi.org/10.3390/ijms140917767.

[46] Ruffion A, Perrin P, Devonec M, Champetier D, Decaussin M, Paparel P, et al. Additional value of PCA3 density to predict initial prostate biopsy outcome. World J Urol 2014;32:917–23. https://doi.org/10.1007/s00345-014-1251-3.

[47] Sanchís-Bonet A, Barrionuevo-González M, Bajo-Chueca AM, Pulido-Fonseca L, Ortega-Polledo LE, Tamayo-Ruiz JC, et al. Validation of the prostate health index in a predictive model of prostate cancer. Actas Urológicas Españolas (English Ed 2018;42:25–32. https://doi.org/10.1016/j.acuroe.2017.11.006.

[48] Sanda MG, Feng Z, Howard DH, Tomlins SA, Sokoll LJ, Chan DW, et al. Association between combined TMPRSS2:ERG and PCA3 RNA urinary testing and detection of aggressive prostate cancer. JAMA Oncol 2017;3:1085–93. https://doi.org/10.1001/jamaoncol.2017.0177.

[49] Schulze A, Christoph F, Sachs M, Schroeder J, Stephan C, Schostak M, et al. Use of the Prostate Health Index and Density in 3 Outpatient Centers to Avoid Unnecessary Prostate Biopsies. Urol Int 2020;104:181–6. https://doi.org/10.1159/000506262.

[50] Seisen T, Rouprêt M, Brault D, Léon P, Cancel-Tassin G, Compérat E, et al. Accuracy of the prostate health index versus the urinary prostate cancer antigen 3 score to predict overall and significant prostate cancer at initial biopsy. Prostate 2015;75:103–11. https://doi.org/10.1002/pros.22898.

[51] Shore N, Hafron J, Langford T, Stein M, Dehart J, Brawer M, et al. Urinary molecular biomarker test impacts prostate biopsy decision making in clinical practice. Urol Pract 2019;6:256–60. https://doi.org/10.1016/j.urpr.2018.09.002.

[52] Steuber T, Heidegger I, Kafka M, Roeder MA, Chun F, Preisser F, et al. PROPOSe: A Real-life Prospective Study of Proclarix, a Novel Blood-based Test to Support Challenging Biopsy Decision-making in Prostate Cancer. Eur Urol Oncol 2021:3–9. https://doi.org/10.1016/j.euo.2020.12.003.

[53] Tan LG, Tan YK, Tai BC, Tan KM, Gauhar V, Tiong HY, et al. Prospective validation of %p2PSA and the Prostate Health Index, in prostate cancer detection in initial prostate biopsies of Asian men, with total PSA 4-10 ng ml-1. Asian J Androl 2017;19:286–90. https://doi.org/10.4103/1008-682X.168687.

[54] Tomlins SA, Day JR, Lonigro RJ, Hovelson DH, Siddiqui J, Kunju LP, et al. Urine TMPRSS2:ERG Plus PCA3 for Individualized Prostate Cancer Risk Assessment. Eur Urol 2016;70:45–53. https://doi.org/10.1016/j.eururo.2015.04.039.

[55] Tosoian JJ, Druskin SC, Andreas D, Mullane P, Chappidi M, Joo S, et al. Use of the prostate health index for detection of prostate cancer: Results from a large academic practice. Prostate Cancer Prostatic Dis 2017;20. https://doi.org/10.1038/pcan.2016.72.

[56] Tosoian JJ, Druskin SC, Andreas D, Mullane P, Chappidi M, Joo S, et al. Prostate Health Index density improves detection of clinically significant prostate cancer. BJU Int 2017;120:793–8. https://doi.org/10.1111/bju.13762.

[57] Tosoian JJ, Trock BJ, Morgan TM, Salami SS, Tomlins SA, Spratt DE, et al. Use of the MyProstateScore Test to Rule Out Clinically Significant Cancer: Validation of a Straightforward Clinical Testing Approach. J Urol 2021;205:732–9. https://doi.org/10.1097/JU.0000000000001430.

[58] Van Neste L, Hendriks RJ, Dijkstra S, Trooskens G, Cornel EB, Jannink SA, et al. Detection of High-grade Prostate Cancer Using a Urinary Molecular Biomarker–Based Risk Score. Eur Urol 2016;70:740–8. https://doi.org/10.1016/j.eururo.2016.04.012.

[59] Wang FB, Chen R, Ren SC, Shi XL, Zhu YS, Zhang W, et al. Prostate cancer antigen 3 moderately improves diagnostic accuracy in Chinese patients undergoing first prostate biopsy. Asian J Androl 2017;19:238–43. https://doi.org/10.4103/1008-682X.167715.

[60] Wei JT, Feng Z, Partin AW, Brown E, Thompson I, Sokoll L, et al. Can urinary PCA3 Supplement PSA in the early detection of prostate cancer? J Clin Oncol 2014;32:4066–72. https://doi.org/10.1200/JCO.2013.52.8505.

[61] Woo J, Santasusagna S, Banks J, Pastor-Lopez S, Yadav K, Carceles-Cordon M, et al. Urine Extracellular Vesicle GATA2 mRNA Discriminates Biopsy Result in Men with Suspicion of Prostate Cancer. J Urol 2020;204:691–700. https://doi.org/10.1097/JU.0000000000001066.

[62] Wu YS, Fu XJ, Na R, Ye DW, Qi J, Lin XL, et al. Phi-based risk calculators performed better in the prediction of prostate cancer in the Chinese population. Asian J Androl 2019;21. https://doi.org/10.4103/aja.aja_125_18.

[63] Wysock JS, Becher E, Persily J, Loeb S, Lepor H. Concordance and Performance of 4Kscore and SelectMDx for Informing Decision to Perform Prostate Biopsy and Detection of Prostate Cancer. Urology 2020;141:119–24. https://doi.org/10.1016/j.urology.2020.02.032.

[64] Yu GP, Na R, Ye DW, Qi J, Liu F, Chen HT, et al. Performance of the Prostate Health Index in predicting prostate biopsy outcomes among men with a negative digital rectal examination and transrectal ultrasonography. Asian J Androl 2016;18:633–8. https://doi.org/10.4103/1008-682X.172823.

[65] Zappala SM, Dong Y, Linder V, Reeve M, Sjoberg DD, Mathur V, et al. The 4Kscore blood test accurately identifies men with aggressive prostate cancer prior to prostate biopsy with or without DRE information. Int J Clin Pract 2017;71:1–8. https://doi.org/10.1111/ijcp.12943.
